# Controlling the fluorescence quantum yields of benzothiazole-difluoroborates by optimal substitution†

**DOI:** 10.1039/d2sc05044g

**Published:** 2022-10-24

**Authors:** Patryk Rybczyński, Manon H. E. Bousquet, Anna Kaczmarek-Kędziera, Beata Jędrzejewska, Denis Jacquemin, Borys Ośmiałowski

**Affiliations:** Faculty of Chemistry, Nicolaus Copernicus University in Toruń Gagarina Street 7 87-100 Toruń Poland borys.osmialowski@umk.pl; Nantes Université, CNRS, CEISAM UMR 6230 F-44000 Nantes France Denis.Jacquemin@univ-nantes.fr; Bydgoszcz University of Science and Technology, Faculty of Chemical Technology and Engineering Seminaryjna 3 85-326 Bydgoszcz Poland; Institut Universitaire de France (IUF) Paris FR-75005 France

## Abstract

Precise tuning of the fluorescence quantum yield, vital for countless applications of fluorophores, remains exceptionally challenging due to numerous factors affecting energy dissipation phenomena often leading to its counterintuitive behavior. In contrast to the absorption and emission wavelength which can be precisely shifted to the desired range by simple structural changes, no general strategy exists for controllable modification of the fluorescence quantum yield. The rigidification of the molecular skeleton is known to usually enhance the emission and can be practically realized *via* the limiting molecular vibrations by aggregation. However, the subtle balance between the abundant possible radiative and non-radiative decay pathways makes the final picture exceptionally sophisticated. In the present study, a series of nine fluorophores obtained by peripheral substitution with two relatively mild electron donating and electron withdrawing groups are reported. The obtained fluorescence quantum yields range from dark to ultra-bright and the extreme values are obtained for the isomeric molecules. These severe changes in emission efficiency have been shown to arise from the complex relationship between the Franck–Condon excited state and conical intersection position. The experimental findings are rationalized by the advanced quantum chemical calculations delivering good correlation between the measured emission parameters and theoretical radiative and internal conversion rate constants. Therefore, the described substituent exchange provides a method to rigorously adjust the properties of molecular probes structurally similar to thioflavin T.

## Abstract 2

A full palette of FQY (form *ca.* 0 to 98%) was covered by exchanging two groups in a series of nine compounds. The darkest (OMe/CF_3_) and brightest (CF_3_/OMe) are isomers. All experimental data are supported by TD-DFT calculations.

## Graphical abstract

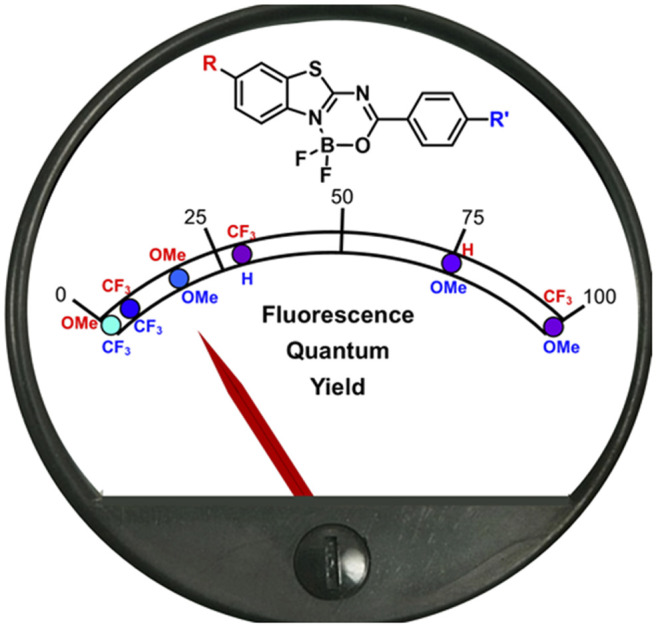

## 1 Introduction

The fine-tuning of light emission of organic dyes is a subject of intensive research due to the rapid development of myriads of fluorescence- and phosphorescence-based technologies. Efforts are focused on optimizing the synthetic methods, improving the purification processes, and, of course, performing structural modifications allowing tailoring the dyes to meet the market demands. Starting from a known emissive core, such an optimization can be achieved through various structural changes including the introduction of side substituent(s), the elongation of the π-conjugation path, atom exchange in the emitter, doubling the acceptor core, *etc.*^1–4^ Among the available fluorescent cores, the BODIPY family, that contains a BF_2_ group providing both improved rigidity and electron-withdrawing character, is clearly one of the most popular platforms.^5,6^ This stems from the high fluorescence quantum yield of the parent BODIPY molecule (close to 100%), and the many synthetic approaches that have been developed for modifying BODIPYs. It is therefore not surprising that BODIPY derivatives are now used in countless areas such as chemosensing,^7,8^ bioimaging,^9,10^ photodynamic therapy,^11,12^ and redox batteries.^13^ For BODIPYs, like for other dyes, it is generally rather straightforward to predict the impact of chemical modifications on the absorption and emission wavelengths. For instance, longer π-conjugation paths typically redshift the fluorescence,^14,15^ while the same may be realized for absorption and emission peaks by introducing an intramolecular charge-transfer (ICT) state caused by an electron acceptor (A) and electron donor (D) attached at two extremities of a dye. In contrast, intuitively predicting the variations of the fluorescence quantum yield (*ϕ*_f_) remains very challenging. For instance, it is known that more rigid molecules exhibit higher *ϕ*_f_ and that redshifting the emission, for instance through a D–A strategy, typically yields a smaller *ϕ*_f_ (the so-called “energy-gap law”), yet these rules-of-thumb remain qualitative, often fail, and are typically not very helpful for practical design. This is because *ϕ*_f_ depends on all non-radiative pathways and their balance,^16^ so that subtle chemical changes might open (or close) some specific routes or, more often, tilt the radiative/non-radiative balance in an unexpected direction. In this framework, it is interesting to stress that first-principles calculations face exactly the same challenges as chemical intuition: whilst emission energies and band shapes can typically be predicted with sufficient reliability for dye design,^17^ accurate *ab initio* predictions of *ϕ*_f_ are in their infancy.^18–26^ Likewise, one might wish to turn towards machine learning approaches, but yet again, they suffer from the same bias: reliable predictions can be obtained for wavelengths, much less so for *ϕ*_f_.^27,28^

Among the ICT dyes, thioflavin T (TT) attracted attention as a dye used for investigating amyloid β (Aβ) aggregation^29–31^ but its usage is not limited to amyloids.^32^ TT is fluorescent but with a low quantum yield in water,^33^ while its brightness significantly improves after aggregation with amyloids, with a *ϕ*_f_ reaching *ca.* 45%.^34^ The aggregation induced emission (AIE) mechanism^35,36^ responsible for this behavior is related to the restriction of molecular motions that inhibit the emission in solution.^37^ Indeed, the non-radiative processes in TT are driven not only by solute–solvent interactions but also by the rotation around a single bond present in its structure,^38,39^ and this rotation is hampered in the aggregated state (see below). From the structural point of view the common feature of many molecules used in studies of Aβ and its aggregation is the presence of a benzothiazole core.^40–42^ This applies to TT that contains a positively charged benzothiazole moiety coupled to a strong electron donating group, namely the *N*,*N*-dimethylamino moiety. In several other series of benzothiazole-based fluorophores a specific twisting mode controls (and often limits) the *ϕ*_f_.^43^ In TT, and in many other compounds, the introduction of the strong electron-donating NMe_2_ moiety advantageously maximizes the ICT and redshifts the emission bands, but unfortunately also reduces *ϕ*_f_ in most solvents,^33^ in part due to the enhanced molecular motions. In contrast, weaker donors might not deliver the desired absorption and emission in the red part of the spectrum. In the same vein, it is also known that most compounds containing the strong electron-accepting NO_2_ group fluoresce weakly,^44^ besides few exceptions.^45^ Therefore, the elimination of the strongest D/A groups from the fluorophore together with the use of multiple substitutions could *a priori* be a smart strategy to bathochromically shift the emission while maintaining a relatively large *ϕ*_f_. Yet again, such “deduction” remains of qualitative nature only.

**Fig. 1:**
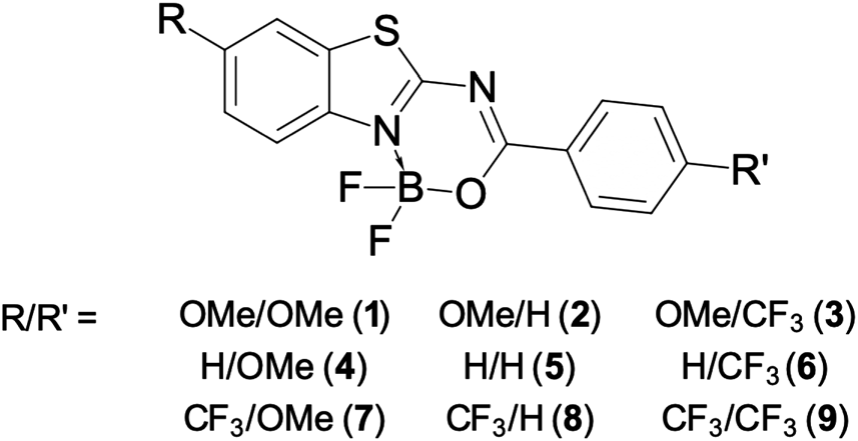
Structure of the nine compounds investigated herein.

The current investigation tackles the impact of double substitution (Fig. 1) on a structural core greatly inspired by TT. The BF_2_ group was introduced to rigidify the skeleton, delivering a core structure combining the advantages of both the BODIPY core and TT. Still, one “twistable” single bond is present in these molecules, which might allow significant relaxation in the excited-state.

Our global target is to understand how the photophysical properties can be controlled by substitution with a rather mild donor (OMe, which is much weaker than NMe_2_) and an inductive acceptor (CF_3_, which is also much less potent than NO_2_; CF_3_ has typically a trifling impact on absorption and emission wavelengths). In more detail, our main goals are: (i) to determine to what extent extremely strong donors or acceptors are mandatory for tuning the photophysical properties of dyes; (ii) to check if the substitution pattern in isomeric molecules significantly impacts their emissive properties; and (iii) to test the molecules topologically similar to TT for their use in various environments. The OMe and CF_3_ groups are introduced systematically in the two substitution points (R and R′ in Fig. 1), namely the benzothiazole fragment (R) and the *para* position of the phenyl ring (R′). The simple exchange of the selected groups between R and R′ in Fig. 1 provides a set of nine compounds exhibiting, as we detail below, non-intuitive patterns in terms of photophysical properties.

## 2 Results and discussion

### 2.1 Experimental

**Fig. 2:**
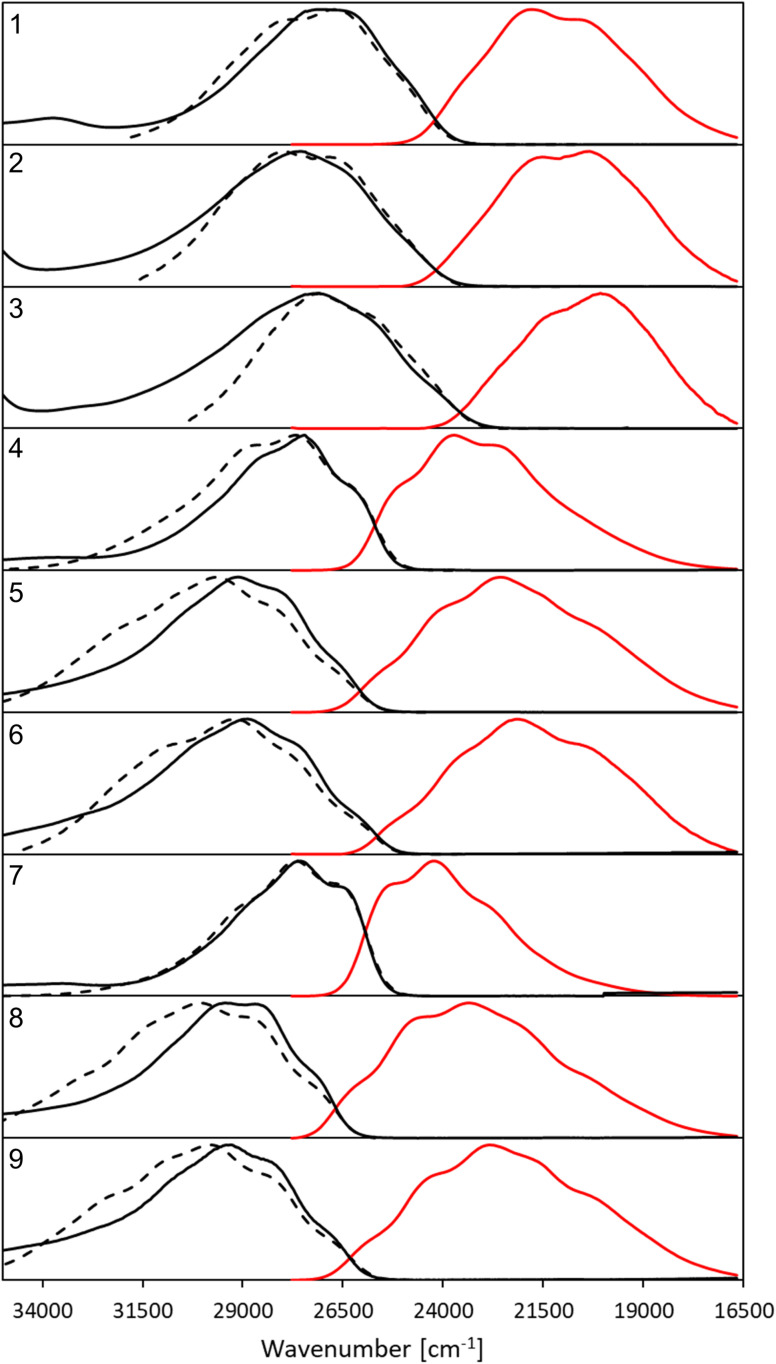
Normalized, corrected absorption (solid black line), fluorescence (solid red line) and mirror-image of fluorescence spectra (dashed black line) shifted to match the 0–0 point in CHCl_3_.

The synthesis of all derivatives is described in the Experimental details section. The key photophysical properties of all dyes measured in chloroform are collected in Table 1. The corresponding absorption (black, solid), emission (red) and the mirrored emission spectra (black, dashed) are shown in Fig. 2.

**Table 1:** Photophysical properties of dyes 1–9 in CHCl_3_: longest wavelength of maximal absorption (nm), molar absorption coefficient (M^−1^ cm^−1^), emission wavelength (nm), Stokes shift (cm^−1^), fluorescence quantum yield (%), full width at half maximum of the absorption and emission bands (cm^−1^), and dye topology. Radiative and non-radiative rates are provided in the ESI

Compound (R/R′)	*λ* ^abs^_max_	*ε*	*λ* ^em^_max_	*Δ*_SS_	*ϕ*_f_	FWHM_abs_	FWHM_em_	Topology
1 (OMe/OMe)	368.0	76 900	454.5	5172	13.1	4246	4519	DAD
2 (OMe/H)	361.0	28 700	458.5	5891	0.9	4691	4586	DA–
3 (OMe/CF_3_)	367.0	26 000	491.5	6902	0.4	5046	4229	DAA
4 (H/OMe)	364.0	46 700	419.5	3635	75.6	3722	4253	–AD
5 (H/H)	343.0	32 100	439.5	6401	6.9	4269	5245	–A–
6 (H/CF_3_)	345.0	27 100	447.0	6614	0.4	4602	5130	–AA
7 (CF_3_/OMe)	362.5	63 500	412.0	3314	98.0	3465	3622	AAD
8 (CF_3_/H)	339.5	43 700	406.0	4825	28.0	4159	4972	AA–
9 (CF_3_/CF_3_)	341.0	30 300	434.5	6311	4.7	4361	5347	AAA

All compounds exhibit a main absorption band between 340 and 370 nm. It is noteworthy that the H-to-CF_3_ substitution has always a very mild impact on both the absorption position and the band shape, whereas the use of the donating OMe group induces noticeable changes, as it tends to bathochromically move the absorption by *ca.* 20–30 nm (Fig. 2). Interestingly, dye 1, that has a DAD quadrupolar topology, presents the most redshifted absorption band, the traditional push–pull strategy represented by compounds 3 and 7 being slightly less effective. This confirms that the BF_2_-substituted benzothiazole is a strongly electron-withdrawing moiety. Notably the intensity of the absorption, as given by *ε*, can be maximized by plugging a methoxy group on the phenyl side (at R′) irrespective of the group substituting the benzothiazole. Nevertheless the *ε* always remains in the 30–80 × 10^3^ M^−1^ cm^−1^ regime, values that are typical of conjugated organic dyes. The substitution of OMe by H and then by CF_3_ in the sub-series (1–3, 4–6, 7–9, see Table 1) always delivers broader absorption bands.

Let us now turn to emission. While, in general, *λ*^em^_max_ values are more variable across the series than their absorption counterparts, the changes in fluorescence spectra remain rather mild for the nine compounds studied herein (Fig. 2). As for the absorption, the influence of the CF_3_ group is weak, whereas OMe has a more clearly detectable (bathochromic) effect, especially when added to the benzothiazole side (R). The presence of the methoxy also partially washes out the vibronic progression. It is 3 that delivers the largest *λ*^em^_max_. Nevertheless, one should be cautious in interpreting directly the *λ*^em^_max_ listed in Table 1. Indeed, as can be seen in Fig. 2, the relative heights of the various vibronic peaks may differ from one compound to another, indicating that the *λ*^em^_max_, related to the most intense vibronic transition, could significantly change, while the actual energy shift between the electronic states (the bands) is mild. In any case, Fig. 2 indicates that the emission and absorption bands are close to mirror-shape hinting that the geometry of the emissive state should not (too) strongly differ from its absorption counterpart. We note that this mirror-shape trend is especially close to perfection for 7 that also displays a notably smaller Stokes shift than the other derivatives. On the other hand, the largest value of the Stokes shift is noted for 3, the only molecule that shows a broader absorption spectrum than the emission. For all dyes, and for both absorption and emission, the full width at half maximum (FWHM) values are in the 3500–5500 cm^−1^ range, which is quite typical of such systems.

Until now, we have not unravelled any specific trends departing from “chemical expectations”, as one could clinically summarize the above findings by stating that all nine dyes share similar absorption and emission features but for slight/significant bathochromic/hyperchromic shifts induced by the methoxy donor. The picture is however much more blurred when investigating the fluorescence quantum yields listed in Table 1. Indeed *ϕ*_f_ is almost 100% for one of the push–pull dyes, 7, but close to zero for 3, presenting inverted R/R′ groups. From the data of Table 1, one can deduce that adding a methoxy substituent at R′ is beneficial in terms of brightness, irrespective of the group at R, whereas, in contrast, CF_3_ group at R position increases the *ϕ*_f_. It is however not easy to interpret such a trend. A first explanation could be that the methoxy group favours a more quinoidal form on the side phenyl group in which the single bond separating the phenyl and benzothiazole moieties gains a double character, what, in turn decreases deleterious excited-state motions. Such changes of bonding pattern would however need to be quite drastic to explain the extreme changes of *ϕ*_f_ noted in the series. In such a scenario, one would also expect the FWHMs, which are roughly related to structural differences between the ground and excited states to significantly change, an unseen outcome. Indeed, the linear determination coefficient (*R*^2^) between *ϕ*_f_ and FWHM_em_ is 0.50 only. Likewise, the “energy-gap law” is not very effective here, since the *R*^2^ obtained by a *ϕ*_f_ to *λ*^em^_max_ relationship attains 0.44. The only experimental parameter nicely correlating with *ϕ*_f_ seems to be the Stokes shift (*R*^2^ = 0.88), but, as stated above, one should consider the *λ*^em^_max_ with caution.

**Fig. 3:**
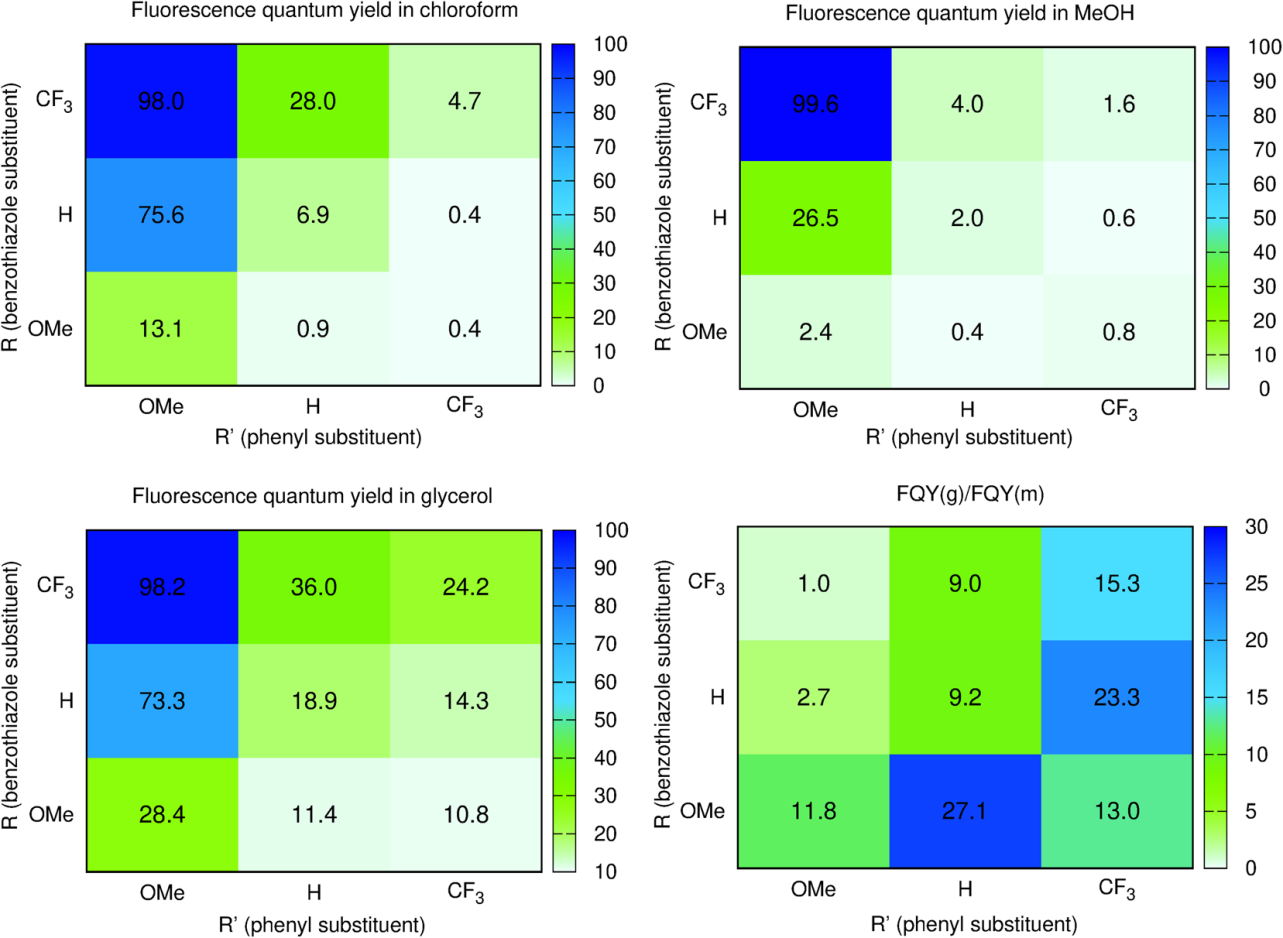
Heatmaps of the fluorescence quantum yields (%) measured in CHCl_3_ (top left), methanol (top right), glycerol (bottom left). The bottom right figure provides the ratio between the glycerol and methanol *ϕ*_f_.

To obtain more insights into the relationship between the *ϕ*_f_ and molecular motions, changing the viscosity of the solution is a rather straightforward experimental approach. We therefore report in Fig. 3 and Table 2 a comparison between the *ϕ*_f_ measured in methanol and glycerol. We have used mixtures of methanol and glycerol to obtain media of controllable viscosity. This pair of solvents was selected as both are able to form hydrogen bonds due to their OH groups, allowing a smooth evolution of the solvents' physicochemical signatures. It is easy to notice that an increased viscosity positively influences the emission properties. Indeed as can be seen in Fig. S2†*ϕ*_f_ increases with the growing viscosity, giving good linear correlation coefficients for the doubly logarithmic plot of *ϕ*_f_ = *f*(*η*) except for 7, which exhibits practically a constant (close to 100%) fluorescence quantum yield irrespective of the medium. The observed relationships are in agreement with the ones of known viscosity-sensitive probes, in which the slope of the linear function is defined as a response factor of the probe.^46^ We therefore underline that, for the current series, the largest slopes are logically obtained for the weakest emitters in non-viscous solvents, *i.e.*, 2 and 6. In contrast, as stated above, 7 enjoys a fluorescence dependent neither upon the modification of the hydrogen bonding nature of the solvent (CHCl_3_ to MeOH) nor upon the medium viscosity (MeOH *vs.* glycerol). This clearly suggests that several non-radiative mechanisms are at play in the series.

**Table 2:** Key emissive properties of 1–9 in MeOH (m), glycerol (g) and response factor (last column, slope), see the caption of Table 1 and text for more details

Compound (R/R′)	*ϕ*_f_ (m)	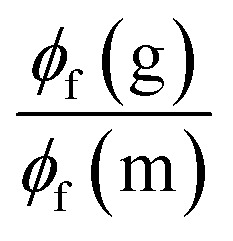	FWHM_em_ (m)	FWHM_em_ (g)	Log(*ϕ*_f_) = *f*(log(*η*)) (slope)
1 (OMe/OMe)	2.4	11.8	5320	5022	0.350
2 (OMe/H)	0.4	27.1	5732	5202	0.441
3 (OMe/CF_3_)	0.8	13.0	5394	5149	0.376
4 (H/OMe)	26.5	2.7	5093	4570	0.148
5 (H/H)	2.0	9.2	6735	5502	0.350
6 (H/CF_3_)	0.6	23.3	6391	5559	0.463
7 (CF_3_/OMe)	99.8	1.0	3585	3445	0.000
8 (CF_3_/H)	4.0	9.0	6303	5364	0.306
9 (CF_3_/CF_3_)	1.6	15.3	6631	5533	0.392

From the points of view of the photophysical properties of the dyes, we note that *ϕ*_f_ is very similar in chloroform (non-viscous, weakly hydrogen-bonding solvent) and glycerol (very viscous and strong hydrogen bonding solvent) for 4, 7, and 8. In contrast, a very low *ϕ*_f_ is recorded for 2, 3, and 6 in both chloroform and methanol but a reasonable increase is noticed in glycerol for these systems. The structural features for those compounds clearly differ from those of 4, 7, and 8, with a donor on the benzothiazole and an electron acceptor on the phenyl or both. This means that reversing the substitution of the acceptor and donor allows going from normal compounds to structures behaving like molecular rotors sensitive to viscosity. Finally, the remaining 1, 5, and 9 are fluorescent in chloroform, very weakly emissive in methanol and their emission is much higher in glycerol. They are simultaneously sensitive to the hydrogen bonding/polar environment and viscosity (see Fig. S1†).

### 2.2 Theory

**Fig. 4:**
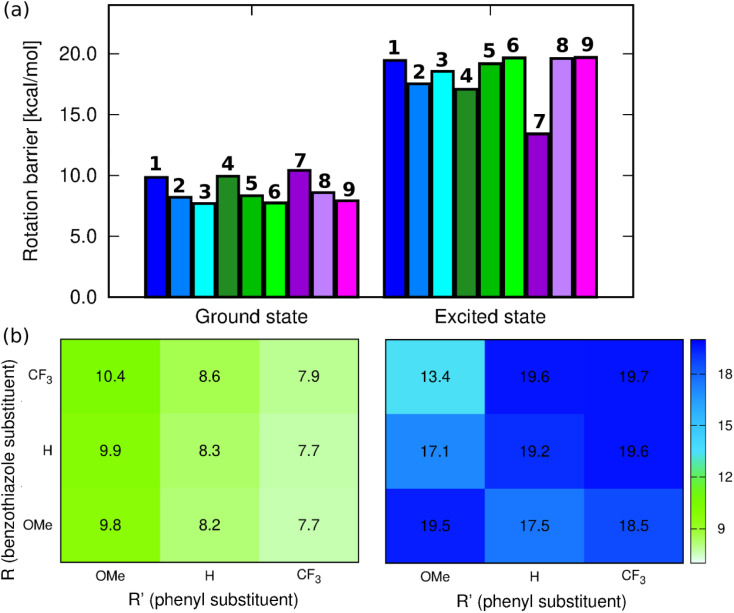
(a) Barriers for rotation around the C15–C17 bond computed at the PCM(CHCl_3_)-(TD-)MN15/6-311++G(d,p)) level for the ground and excited state geometries, (b) corresponding heatmaps.

We have used *ab initio* calculations to probe the excited state (ES) features of all nine compounds. We have decided to consider CHCl_3_ as the medium for these simulations as the theoretical protocol relies on a continuum model for the environmental effects (see Experimental details section for technical specification). The careful inspection of the geometry of the investigated systems in the two electronic states confirms that the excitation promotes the *p*-quinoid form of the terminal phenyl ring. This can be deduced from the respective shortenings and elongations of the alternate C–C bonds to form the =C(–CH=CH–)_2_C= moiety. These changes are similar to those found previously in NMe_2_-substituted benzothiazole derivatives.^47^ Since one of the envisaged deactivation pathways involves the rotation of the phenyl ring, (TD-)DFT calculations have been performed to quantify the related rotation barrier. Full DFT geometry optimizations of the transition state for the rotation around the C–C bond (C15–C17 in Fig. S52†) provide a ground-state picture consistent with chemical intuition. Indeed, as can be seen in Fig. 4, increasing the electron-donating character of the substituent at R′ causes increase of the rotation barrier by *ca.* 2 kcal mol^−1^, irrespective of the group at R, which fits with the quinoidal picture leading to a strengthening of the inter-ring bond upon addition of a mesomeric donor at R′. We also note that increasing the electron-accepting character of the substituent at the benzothiazole moiety induces a slight increase of the barrier, but the effect (*ca.* 0.5 kcal mol^−1^) is insignificant for a DFT calculation. The largest ground-state barrier, 10.4 kcal mol^−1^, is observed for 7, the lowest one for 3, 7.68 kcal mol^−1^. If such a pattern seems to fit the observed *ϕ*_f_ ranking (larger barriers, larger emission yields), one has to remember that these values are valid for the ground electronic state, *i.e.*, they are not representative of the structures of the emissive species.

In contrast, the ES picture does not follow a clear systematic order nor correlates with the experimental *ϕ*_f_ (see Fig. 4). Interestingly, the ES rotation barriers are significantly higher than in the ground state, 8-out-of-9 exceeding 17 kcal mol^−1^, 7 being the only compound presenting a rather low ES barrier (13.4 kcal mol^−1^). If this smaller change of bond strength between the ground and excited states for 7 is consistent with the trends in the measured Stokes shift and almost perfect mirror symmetry of absorption and emission spectra, it is clearly not intuitive that the dye showing, by far, the lowest excited-state rotation barrier also displays the largest *ϕ*_f_. We note that the increase of barriers after photon absorption is consistent with chemical intuition (π–π* excitation decreasing the bond length alternation) and also with the Wiberg bond indexes being larger by 0.1 to 0.2 in the excited than in the ground state, a statement holding for all molecules (see Table S4†).

Let us now turn towards the charge transfer properties, *i.e.*, the transferred charge, *q*_CT_, the distance between the barycenters of density gain and depletion, *d*_CT_, and the variation of the dipole moment between the states, Δ*μ*. Table 3 reports these parameters together with density difference plots. The gain of density is always localized on the fluoroborate ring, whereas the loss of density varies from one compound to another, the role of the OMe group being clear. This is confirmed by the positions of the barycenters (see the ESI†): R_+_ remains almost unaffected by the variation of the substituents whereas R_−_ depends on the considered dye. Among the analyzed fluorophores, the strongest photoinduced charge transfer appears for 3 which undergoes an 8.7 D dipole increase in the ES, corresponding to a displacement of 0.65*e* over 3.2 Å, quite large values in Le Bahers' model.^49^ There is a weak positive correlation between *d*_CT_ and *λ*^em^_max_ with an *R*^2^ of 0.61. Nevertheless, as for the barriers above, the CT parameters do not provide reasonable insights into the emission yields. Indeed, the *R*^2^ between the measured *ϕ*_f_ and *d*_CT_, *q*_CT_, and Δ*μ* are all tiny: 0.12, 0.03, and 0.04, respectively. In other words, the magnitude of the charge-transfer does not control the fluorescence efficiency.

**Table 3:** Electron density difference (EDD, 0.002 au contour) plots and charge-transfer parameters^48^ determined at the PCM(CHCl_3_)-TD-MN15/6-311++G(d,p) level on the ground state optimized geometry. Blue (red) surface marks the electron density depletion (gain) upon excitation. *d*_CT_ is given in Å, *q*_CT_ in a.u., Δ*μ* = *μ*_ES_ − *μ*_GS_ and dipole moments in D. The dipole moments of the optimized geometry of the excited state can be found in the ESI

Compound (R/R′)	EDD	*d*_CT_	*q*_CT_	*μ*_GS_	*μ*_ES_	Δ*μ*
1 (OMe/OMe)	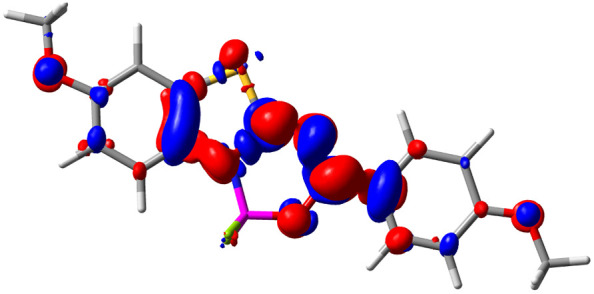	0.880	0.575	4.881	5.411	0.530
2 (OMe/H)	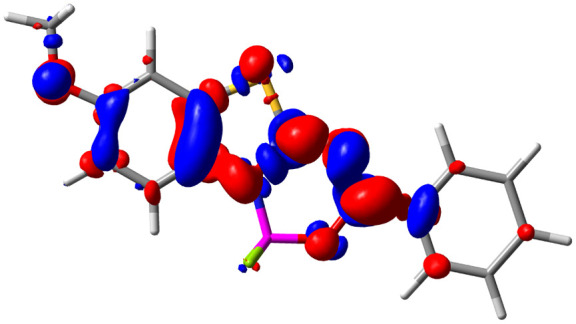	2.547	0.606	6.605	11.036	4.432
3 (OMe/CF_3_)	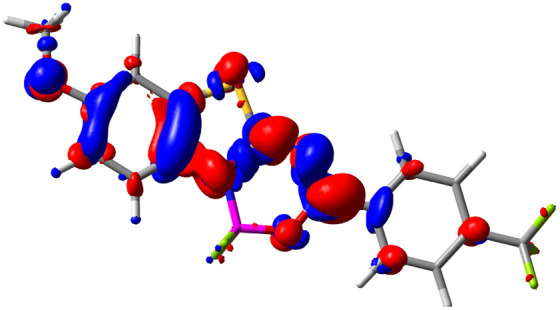	3.271	0.651	8.376	17.181	8.806
4 (H/OMe)	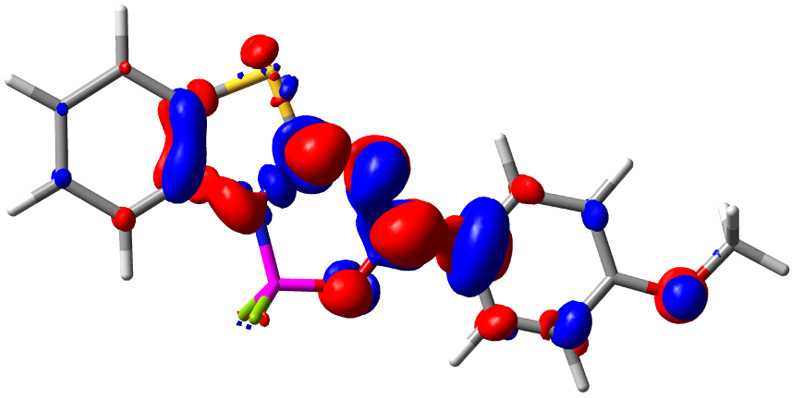	0.904	0.545	6.729	7.816	1.088
5 (H/H)	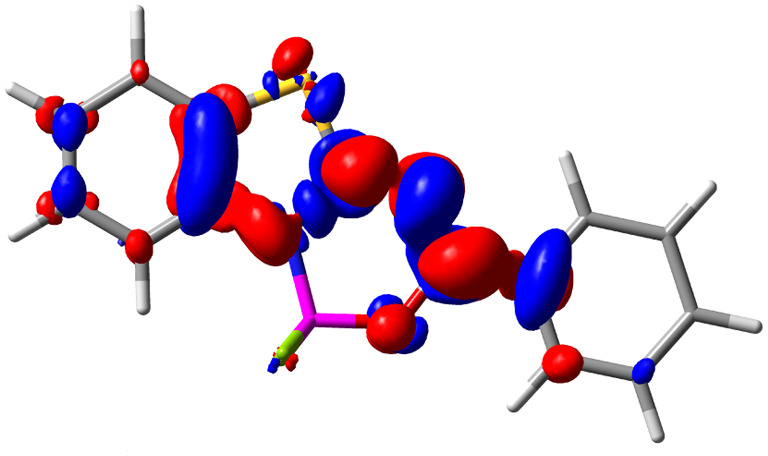	1.147	0.514	4.887	6.147	1.259
6 (H/CF_3_)	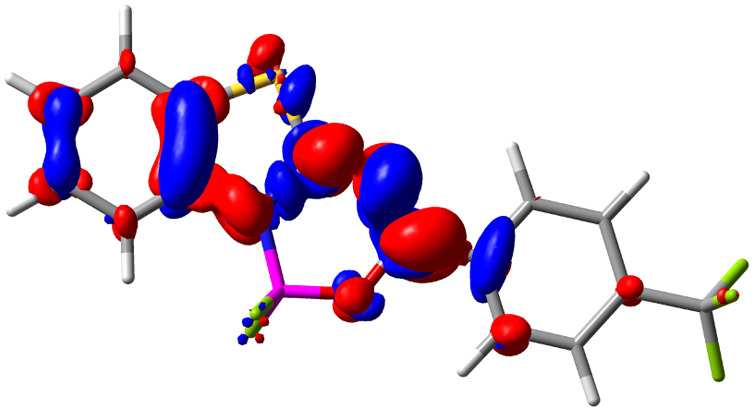	2.119	0.540	6.644	11.427	4.783
7 (CF_3_/OMe)	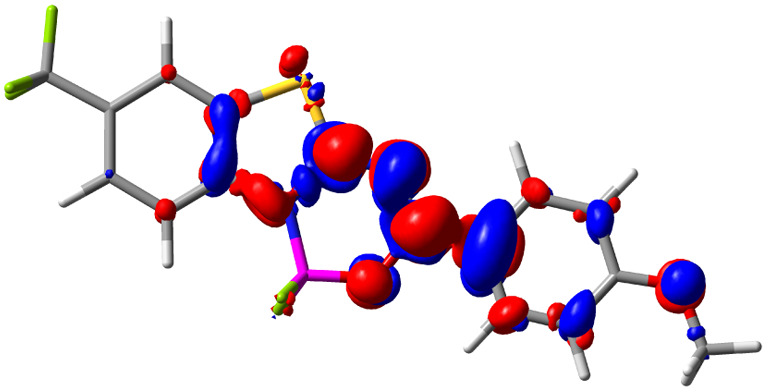	1.658	0.551	6.427	10.609	4.181
8 (CF_3_/H)	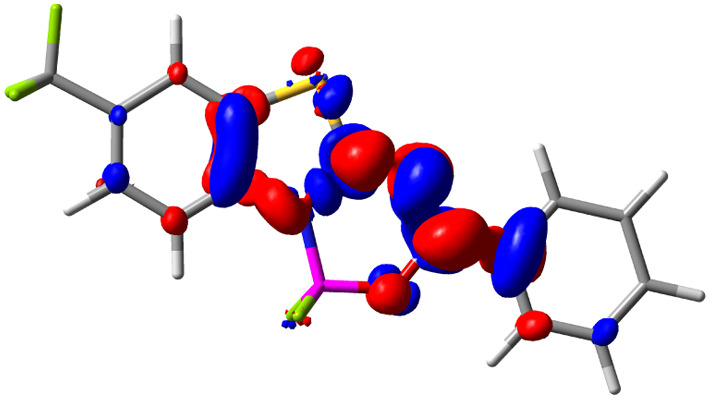	0.412	0.495	5.736	5.501	−0.235
9 (CF_3_/CF_3_)	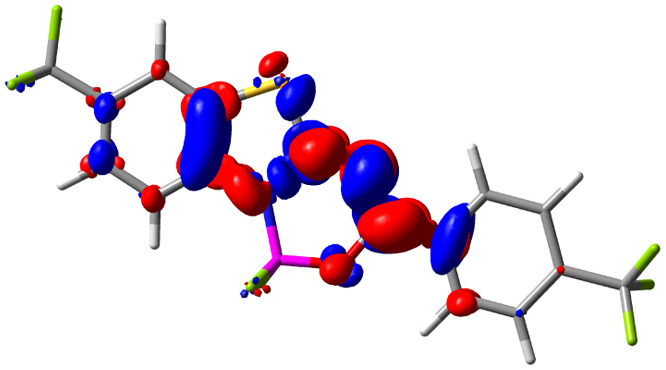	1.479	0.503	4.056	6.091	2.036

Given the above rather unfruitful attempts to rationalize the measured *ϕ*_f_, we decided to search for the most accessible pathways allowing to directly go back from the excited to the ground electronic state through non-radiative decay. First, given the presence of a sulfur atom, we evaluated the possibility of intersystem crossing (ISC), by evaluating the singlet–triplet gap and the spin–orbit coupling (SOC) at the relaxed excited state geometry. The results are listed in Table 4. Only one triplet is below S_1_, and as can be seen in that table the gaps are typically quite large (at least 0.24 eV) while the SOC matrix elements are truly trifling (<0.05 cm^−1^). This strongly hints that ISC is not a major pathway for the considered systems.

**Table 4:** Singlet–triplet gap computed on the relaxed S_1_ geometry (eV), with the corresponding SOC (cm^−1^). Energy difference between the most accessible MECP and the FC point calculated at the PCM(CHCl_3_)-SF-TD-MN15/6-311+G(d,p) level (eV). Radiative and non-radiative rates as obtained by theory (10^8^ s^−1^). Theoretical fluorescence quantum yields (%)

Compound (R/R′)	Δ*E*^S_1_–T_1_^	SOC	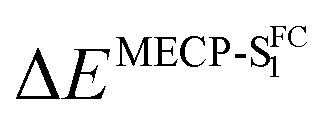	*k*_r_	*k*_ic_	*k*_MECP_	*ϕ* ^no-MECP^_f_ a	*ϕ* ^MECP^_f_ b
1 (OMe/OMe)	0.46	0.04	0.06	3.09	0.83	5060	79	0.0
2 (OMe/H)	0.66	0.03	−0.14	2.18	7.87	∞	22	0.0
3 (OMe/CF_3_)	0.73	0.04	−0.27	2.02	2.16	∞	48	0.0
4 (H/OMe)	0.28	0.03	0.47	4.76	0.36	0.00	93	93
5 (H/H)	0.44	0.03	0.12	3.21	0.57	682	85	0.1
6 (H/CF_3_)	0.52	0.03	−0.07	2.70	1.50	∞	64	0.0
7 (CF_3_/OMe)	0.24	0.04	0.86	5.61	0.26	0.00	96	96
8 (CF_3_/H)	0.36	c	0.56	3.92	0.28	0.00	93	93
9 (CF_3_/CF_3_)	0.44	0.04	0.10	2.78	2.14	1250	56	0.0

a Computed considering only the radiative and internal conversion rates.

b Considering the radiative, internal and MECP pathways.

c SOC calculation did not converge.

**Fig. 5:**
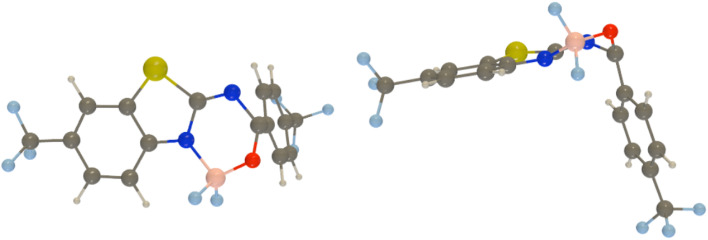
Two views of the MECP between the ground and the lowest singlet excited states as obtained at the PCM(CHCl_3_)-SF-TD-MN15/6-311+G(d,p) level of theory for 9. Similar MECPs are found for all dyes.

Next, we used spin-flip (SF) TD-DFT to locate the most accessible minimum energy crossing point (MECP) between the ground and excited states for all dyes. Indeed, several previous studies have demonstrated a direct relationship between the measured emission yield and the relative energy of the lowest MECP.^18,21,50,51^ Using the procedure described in the Experimental section, we found that this most accessible MECP corresponds, in all cases, to a strongly deformed geometry with an almost 90° kink between the two parts of the molecule (see Fig. 5). The relative SF-TD-DFT energies of this MECP and the S_1_ FC point are given in Table 4. Despite several attempts, we could not locate a proper transition state corresponding to the path to this MECP, likely indicating that the path is smooth and that these relative energies are indicators of the accessibility of this MECP. Interestingly, we note that the three dyes having an MECP more stable than the FC point are 2, 3, and 6, that is, the three experimentally “dark” compounds, *i.e.*, compounds showing *ϕ*_f_ smaller than 1% (see Table 1). This is consistent with a downhill path to the MECP. Likewise, the three compounds for which reaching the MECP implies an energetic penalty larger than 0.3 eV, namely, 4, 8, and 7 are the only three showing rather large (>25%) *ϕ*_f_ in the experiment. We note that this is the first clear correlation between theoretical estimates and experimental *ϕ*_f_ found in this work. It is worth noting that the *R*^2^ = 0.81 for the correlation between these energies and the measured *ϕ*_f_ (in glycerol); a very similar value (*R*^2^ = 0.78) is obtained in chloroform which was used in measurements and calculations. The slightly higher value for glycerol may be due to the higher rigidity of the molecules in that solvent, a feature of well-defined geometry as in calculations limiting additional dynamic effects associated with energy dissipation.

**Fig. 6:**
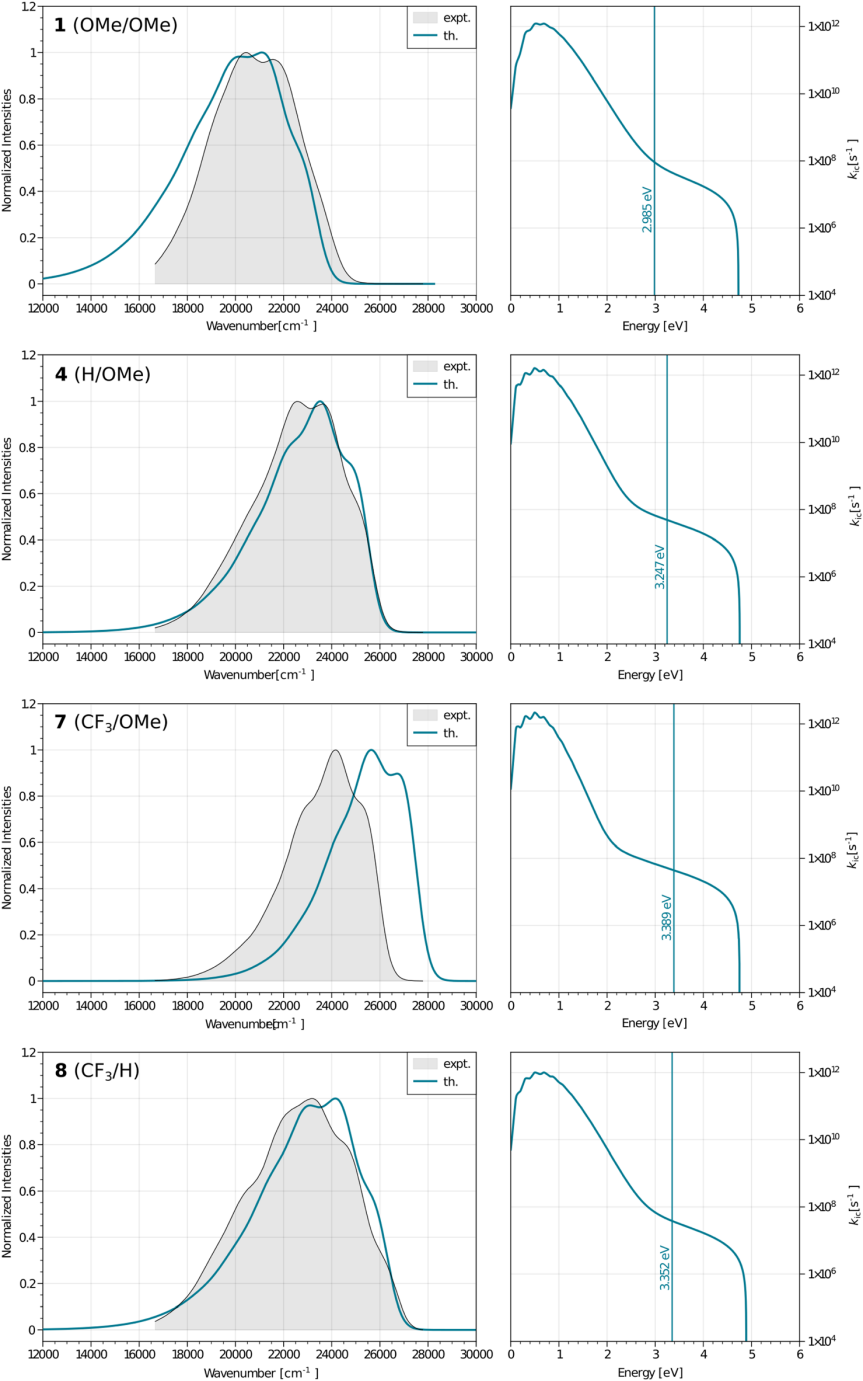
Left: computed (colored lines) and measured (black lines and grey background) fluorescence spectra for the four brightest dyes. Right: plots showing the dependence between the internal conversion rates and the energies for the same four systems, the vertical line gives the computed adiabatic energy.

To go further, we have used Peng and Shuai's TVCF formalism to model both the radiative and internal conversion rates of all dyes on an equal footing (again see the Experimental section for details).^52,53^ Such calculations involve the calculations of vibronic couplings on the basis of harmonic vibrational frequencies, as well as the selection of models for the coupling between the potential energy surfaces, the coordinates, *etc.* After some tests, we have chosen the most refined model (so-called adiabatic Hessian), in a time-dependent framework and selected Cartesian coordinates – the computed rates are listed in Table 4. In Fig. 6, one can find comparisons between the theoretical and experimental emission lineshapes for the four brightest derivatives and the agreement gives confidence that theory correctly describes the vibronic couplings for the present class of derivatives. The *ab initio* radiative rates are in the 10^8^ s^−1^ range, which fits the experimental trends (see the ESI†), the largest value being computed for 7, 5.61 × 10^8^ s^−1^, a result perfectly consistent with the measurement, 5.93 × 10^8^ s^−1^. The *R*^2^ between experiment and theory for the *k*_r_ attains a very satisfying 0.87 value. We note nevertheless that the absolute magnitudes of the theoretical radiative rates significantly overestimate their experimental counterparts for the poorly emissive dyes, but for these almost dark compounds the experimental error bar becomes (much) larger, so it is not so straightforward to pinpoint if theory or experiment is faulty in that case. The internal conversion rates, *k*_ic_, measure the non-radiative deactivation through non-adiabatic couplings between the excited and ground states. The computed values are in the 2 × 10^7^ to 8 × 10^8^ s^−1^ range (see Table 4), *i.e.*, cover a quite broad panel of values, yet these *k*_ic_ have in general no direct experimental counterparts. In addition, we wish to stress the inherent limits of theory in evaluation of this rate, as it is rather common that *k*_ic_ strongly depends on the selected broadening, in contrast to *k*_r_.^22^ In the ESI Tables S6 and S7,† we provide comparisons between IC rates computed with different vibronic models, and one notices that the IC rate varies significantly depending on the selected model, but those changes do not impact the relatively poor correlation with experimental data.

By computing the *ϕ*_f_ on the basis of radiative and internal conversion rates only, one can estimate the emission yield if no other non-radiative processes take place, meaning, in the present case, that the MECPs would always be inaccessible. In this way, too large *ϕ*_f_ values are obtained but one notices that a value of 96%, consistent with the measurements, is determined for 7 which also presents the least accessible MECP (see above). Finally, it is possible to compute the non-radiative rate constant related to the MECP simply using1
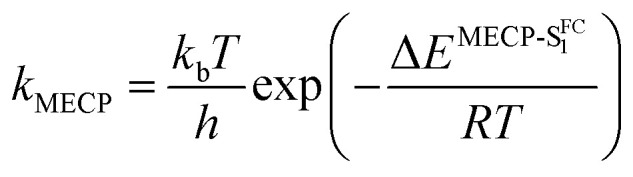
in an approach equivalent to the one used previously for BODIPYs.^21^ As can be seen in Table 4 such a more complete approach would split the nine dyes into two groups: (highly) emissive and non-fluorescent, and these groups are close to the experimental ones. In fact, taking the experimental *ϕ*_f_ as the reference, the errors are smaller than 0.1, but for three compounds, namely: 1 which is incorrectly predicted to be totally dark whereas the experimental yield is 13%, 4 for which theory overshoots the *ϕ*_f_ (93 *versus* 76%), and 8 which is the worst failure to our views, as theory grossly overestimates the yield (93 *versus* 28%). These deviations are likely significant revealing the inherent limitations of the theoretical model (both electronic structure and vibronic calculations). Alternatively, one could indeed compute *k*_MECP_ using as reference the energy of the relaxed S_1_ geometry instead of the FC point. This has been investigated and the results are displayed in Fig. S60 in the ESI† showing that the correlation with the experimental quantum yields is not improved in this case. For compound 1, computing the MECP rate from the FC point leads to *k*_MECP_ = 5.06 × 10^11^ s^−1^ and hence a negligible fluorescence yield, whereas from the relaxed S_1_ one obtains *k*_MECP_ = 3.24 × 10^5^ s^−1^ and a very large fluorescence yield. Obviously, the experimental truth stands in between, and this illustrates the limits of static approaches: one can estimate high and low limit values for the MECP rate, whereas a dynamical approach would likely be needed to obtain a more balanced description.

## 3 Conclusions and outlook

The series of benzothiazoles considered here shows that the fluorescence quantum yield may be tuned systematically and strongly by exchanging two (relatively mild) groups located on the periphery of the structures only. From the intuitive point of view this seems to be realised by modifying the height of the rotation barrier around the only single bond in the structure, as a viscous environment is beneficial for the brightness of the synthesized dyes, indicating the presence of significant detrimental motions. However, theoretical calculations indicate that these motions correspond to the kinking of the structure around the single bond, rather than a rotation. Indeed, the difference of excited state energy between the FC point and the minimum energy crossing point between ground and excited states strongly correlates with experimental fluorescence quantum yield. This correlation is even higher in viscous environments. We conclude that simple substituent exchange by no means induces an intuitive effect though such an approach delivers significant changes in the emission. The current series undoubtedly shows that substituents being not particularly potent ones in light of their electronic effects may be applied to cover the full range of emission quantum yields. This feature may be further adapted in the design of rotor-based/conical intersection-tuned emitters capable of sensing the viscosity and aggregation, and thus in molecular probes for a variety of needs including biochemistry-oriented ones.

## 4 Experimental details

### 4.1 Synthesis and structure confirmation

All substrates for the synthesis (amines and benzoyl chlorides) and solvents were obtained from commercial sources. The studied series of compounds was obtained by a two-step synthesis. The amide: to a solution of 2-aminobenzothiazole (1 eq.) and triethylamine (2 eq.) in dry THF (20 ml, −78 °C, under an inert gas) the respective benzoyl chloride (1 eq.) was added dropwise (in dry THF). The mixture was allowed to warm to room temperature and stirred overnight. The solvent was evaporated and the residue extracted using DCM/NaHCO_3_ water solution. After evaporation of the organic layer the compound was purified by column chromatography (eluent hexane/ethyl acetate). In the second step, the appropriate amide was complexed with BF_3_·OEt_2_. The etherate was added dropwise to a mixture of amide (1 eq.) and diisopropylethylamine (3 eq.) in DCM (20 ml, room temperature). The reaction mixture was stirred overnight and then the DCM was evaporated. The compound was purified by column chromatography (SiO_2_, eluent DCM).

The structure of the synthesized compounds was confirmed by NMR and MS techniques. All of the NMR spectra were recorded at 400 MHz or 700 MHz on a Bruker spectrometer at room temperature in CDCl_3_. HRMS data were obtained with a Waters spectrometer in the EI mode. The melting points were measured using a Stuart SMP50 Digital Melting Point Instrument.

#### 4.1.1 Compound 1

((*Z*)-[(Difluoroboryloxy)(*p*-methoxyphenyl)methylene](6-methoxy-1,3-benzothiazol-2-yl)amine, OMe/OMe) yield 56.7%. Mp 215–217 °C, green powder ^1^H NMR (700 MHz, from TMS, CDCl_3_): *δ* (ppm) 8.36 (d, 2H, *J* = 9.03 Hz), 7.95 (d, 1H, *J* = 9.03 Hz), 7.26 (d, 1H, *J* = 2.52 Hz), 7.18 (dd, 1H, *J* = 9.10, 2.52 Hz), 7.00 (d, 2H, *J* = 8.96 Hz), 3.93 (s, 3H), 3.91 (s, 3H). ^11^B (128 MHz, from BF_3_·Et_2_O, CDCl_3_): *δ* (ppm) 0.576 (t). ^13^C (100 MHz, from TMS, DMSO-d_6_) *δ* (ppm): 172.3, 167.4, 164.8, 134.1, 132.7, 128.4, 123.3, 119.2, 116.8, 114.1, 105.3, 56.0, 55.6. ^19^F (376 MHz, from CFCl_3_, DMSO-d_6_): *δ* (ppm) −137.6. EI HRMS (*m*/*z*) [M]^+^ 362.0703 cal. 362.0708.

#### 4.1.2 Compound 2

((*Z*)-[(Difluoroboryloxy)phenylmethylene](6-methoxy-1,3-benzothiazol-2-yl)amine, OMe/H) yield 63.4%. Mp 201–202 °C, green powder, ^1^H NMR (400 MHz, from TMS, CDCl_3_): *δ* (ppm) 8.40 (d, 2H, *J* = 8.50 Hz), 7.66 (t, 1H, *J* = 7.56 Hz), 7.53 (dd, 2H, *J* = 7.82 Hz), 7.28 (d, 1H, *J* = 2.65 Hz), 7.20 (dd, 1H, *J* = 9.04, 2.16 Hz), 3.92 (s, 3H). ^11^B (128 MHz, from BF_3_·Et_2_O, CDCl_3_): *δ* (ppm) 1.04 (t). ^13^C (100 MHz, from TMS, CDCl_3_) *δ* (ppm): 172.2, 167.7, 158.6, 134.2, 134.0, 131.1, 130.9, 128.6, 119.4, 117.1, 105.2, 56.0. ^19^F (376 MHz, from CFCl_3_, DMSO-d_6_): *δ* (ppm) −137.0. EI HRMS (*m*/*z*) [M]^+^ 332.0605 cal. 332.0602.

#### 4.1.3 Compound 3

((*Z*)-(Difluoroboryloxy)[*p*-(trifluoromethyl)phenyl]methylene(6-methoxy-1,3-benzothiazol-2-yl)amine, OMe/CF_3_) yield 80.2%. Mp 251–252 °C, green powder ^1^H NMR (400 MHz, from TMS, CDCl_3_): *δ* (ppm) 8.51 (d, 2H, *J* = 8.08 Hz), 8.01 (d, 1H, *J* = 9.08 Hz), 7.80 (d, 2H, *J* = 8.20 Hz), 7.31 (d, 1H, *J* = 2.36 Hz), 7.24 (dd, 1H, *J* = 9.12, 2.52 Hz), 3.94 (s, 3H). ^11^B (128 MHz, from BF_3_·Et_2_O, CDCl_3_): *δ* (ppm) 0.64 (t). ^13^C (100 MHz, from TMS, CDCl_3_) *δ* (ppm): 171.7, 166.1, 158.8, 135.3, 135.1, 134.5, 133.9, 130.5, 129.1, 125.6, 124.4, 122.8, 119.7, 117.5, 105.1, 56.0. ^19^F (376 MHz, from CFCl_3_, DMSO-d_6_): *δ* (ppm) −64.1, −136.6. EI HRMS (*m*/*z*) [M]^+^ 400.0478 cal. 400.0476.

#### 4.1.4 Compound 4

((*Z*)-[(Difluoroboryloxy)(*p*-methoxyphenyl)methylene]-1,3-benzothiazol-2-ylamine, H/OMe) yield 82.7%. Mp 215–216 °C, green powder ^1^H NMR (400 MHz, from TMS, CDCl_3_): *δ* (ppm) 8.39 (d, 2H, *J* = 9.02 Hz), 8.06 (d, 1H, *J* = 8.08 Hz), 7.81 (d, 1H, *J* = 8.36 Hz), 7.61 (m, 1H), 7.49 (m, 1H), 7.02 (d, 2H, *J* = 9.02 Hz) 3.94 (s, 3H). ^11^B (128 MHz, from BF_3_·Et_2_O, CDCl_3_): *δ* (ppm) 1.03 (t). ^13^C (100 MHz, from TMS, CDCl_3_) *δ* (ppm): 174.0, 168.1, 165.0, 140.1, 132.9, 131.7, 128.2, 126.2, 123.1, 122.1, 118.4, 114.1, 55.6. ^19^F (376 MHz, from CFCl_3_, CDCl_3_): *δ* (ppm) −137.4. EI HRMS (*m*/*z*) [M]^+^ 332.0601 cal. 332.0602.

#### 4.1.5 Compound 5

((*Z*)-[(Difluoroboryloxy)phenylmethylene]-1,3-benzothiazol-2-ylamine, H/H) yield 66.8%. Mp 197–198 °C, white powder ^1^H NMR (400 MHz, from TMS, CDCl_3_): *δ* (ppm) 8.42 (dd, 2H, *J* = 8.55 Hz), 8.09 (d, 1H, *J* = 9.08 Hz), 7.83 (d, 1H, *J* = 8.68 Hz), 7.67 (m, 1H), 7.63 (m, 1H), 7.54 (m, 2H) 7.51 (m, 1H). ^11^B (128 MHz, from BF_3_·Et_2_O, CDCl_3_): *δ* (ppm) 1.10 (t). ^13^C (100 MHz, from TMS, CDCl_3_) *δ* (ppm): 174.0, 168.5, 140.1, 134.5, 131.0, 128.7, 128.4, 127.2, 126.6, 122.2, 118.7. ^19^F (376 MHz, from CFCl_3_, CDCl_3_): *δ* (ppm) −136.7. EI HRMS (*m*/*z*) [M]^+^ 302.0501 cal. 302.0497.

#### 4.1.6 Compound 6

((*Z*)-(Difluoroboryloxy)[*p*-(trifluoromethyl)phenyl]methylene-1,3-benzothiazol-2-ylamine, H/CF_3_) yield 90.1%. Mp 176–177 °C, white powder ^1^H NMR (400 MHz, from TMS, CDCl_3_): *δ* (ppm) 8.52 (d, 2H, *J* = 8.15 Hz), 8.12 (d, 1H, *J* = 8.17 Hz), 7.86 (d, 2H, *J* = 8.20 Hz), 7.80 (d, 2H, *J* = 2.36 Hz), 7.67 (dd, 1H, *J* = 7.58 Hz), 7.67 (dd, 1H, *J* = 7.32 Hz). ^11^B (128 MHz, from BF_3_·Et_2_O, CDCl_3_): *δ* (ppm) 1.10 (t). ^13^C (100 MHz, from TMS, CDCl_3_) *δ* (ppm): 166.8, 139.9, 135.6, 135.1, 134.3, 130.6, 128.6, 125.6, 125.5, 125.3, 122.2, 121.7, 118.8. ^19^F (376 MHz, from CFCl_3_, DMSO-d_6_): *δ* (ppm) −64.1, −136.6. EI HRMS (*m*/*z*) [M]^+^ 370.0375 cal. 370.0371.

#### 4.1.7 Compound 7

((*Z*)-[(Difluoroboryloxy)(*p*-methoxyphenyl)methylene][6-(trifluoromethyl)-1,3-benzothiazol-2-yl]amine, CF_3_/OMe) yield 53.3%. Mp 288–289 °C, green powder ^1^H NMR (700 MHz, from TMS, CDCl_3_): *δ* (ppm) 8.38 (d, 2H, *J* = 8.89 Hz), 8.12 (d, 1H, *J* = 8.61 Hz), 8.08 (s, 1H), 7.83 (dd, 1H, *J* = 8.70, 1.42), 7.00 (d, 1H, *J* = 9.10 Hz), 3.93 (s, 3H). ^11^B (128 MHz, from BF_3_·Et_2_O, CDCl_3_): *δ* (ppm) 0.66 (t). ^13^C (100 MHz, from TMS, CDCl_3_) *δ* (ppm): 175.6, 169.2, 165.2, 142.5, 133.3, 128.7, 127.2, 125.3, 122.8, 119.7, 118.7, 114.3, 55.65. ^19^F (376 MHz, from CFCl_3_, DMSO-d_6_): *δ* (ppm) −62.85. EI HRMS (*m*/*z*) [M]^+^ 400.0478 cal. 400.0476.

#### 4.1.8 Compound 8

((*Z*)-[(Difluoroboryloxy)phenylmethylene][6-(trifluoromethyl)-1,3-benzothiazol-2-yl]amine, CF_3_/H) yield 77.9%. Mp 249–250 °C, white powder ^1^H NMR (400 MHz, from TMS, CDCl_3_): *δ* (ppm) 8.44 (d, 2H, *J* = 8.08 Hz), 8.19 (d, 1H, *J* = 9.08 Hz), 8.14 (m, 1H), 7.88 (dd, 1H, *J* = 8.78, 1.31 Hz), 7.71 (m, 1H), 7.57 (m, 2H), 3.94 (s, 3H). ^11^B (128 MHz, from BF_3_·Et_2_O, CDCl_3_): *δ* (ppm) 0.73 (t). ^13^C (100 MHz, from TMS, CDCl_3_) *δ* (ppm): 175.8, 169.5, 142.3, 135.1, 130.8, 130.6, 129.1, 128.8, 128.7, 127.4, 125.6, 125.3, 121.7, 119.9, 119.0, 118.1. ^19^F (376 MHz, from CFCl_3_, DMSO-d_6_): *δ* (ppm) −62.9, −136.6. EI HRMS (*m*/*z*) [M]^+^ 370.0375 cal. 370.0371.

#### 4.1.9 Compound 9

((*Z*)-(Difluoroboryloxy)[*p*-(trifluoromethyl)phenyl]methylene[6-(trifluoromethyl)-1,3-benzothiazol-2-yl]amine, CF_3_/CF_3_) yield 85.0%. Mp 237–238 °C, white powder ^1^H NMR (400 MHz, from TMS, CDCl_3_): *δ* (ppm) 8.53 (d, 2H, *J* = 8.23 Hz), 8.20 (d, 1H, *J* = 8.83 Hz), 7.80 (d, 1H, *J* = 8.20 Hz), 7.31 (m, 1H), 7.24 (dd, 1H, *J* = 8.77, 1.19 Hz), 7.80 (d, 2H, *J* = 8.49 Hz). ^11^B (128 MHz, from BF_3_·Et_2_O, CDCl_3_): *δ* (ppm) 0.65 (t). ^13^C (100 MHz, from TMS, CDCl_3_) *δ* (ppm): 175.6, 168.0, 142.2, 136.1, 134.0, 131.0, 129.1, 127.7, 125.8, 124.8, 122.1, 120.0, 119.3. ^19^F (376 MHz, from CFCl_3_): *δ* (ppm) −64.2, −62.9, −135.8. EI HRMS (*m*/*z*) [M]^+^ 438.0239 cal. 438.0244.

### 4.2 Optical measurements

UV-vis absorption spectra of *ca.* 10^−5^ M solutions of dyes were recorded in quartz cells (1 cm) using a Shimadzu UV-1900 spectrometer. Fluorescence spectra of *ca.* 10^−6^ M solutions of complexes were recorded in quartz cells (1 cm) using an FS5 fluorimeter (Edinburgh Instruments). Fluorescence quantum yield measurements were carried out on the same equipment with the use of an integrating sphere. Time-correlated single-photon counting measurements were performed with an Edinburgh Analytical Instruments F920P spectrometer. Samples were excited at 373 nm using a laser diode. The emission intensity was recorded at fluorescence maximum wavelength. A solution of colloidal silica was used to obtain the instrument response function (IRF). Fluorescence lifetimes were calculated using the FAST software.

### 4.3 Calculations

Geometry optimizations of the ground and excited state have been performed within the (TD-)MN15 (ref. 54) approach known to deliver especially accurate energies for compounds of the family investigated here^55^ using either the 6-311++G(d,p) or 6-311+G(d,p) basis sets, the former being applied for CT parameters and rotational barrier, the (more compact) latter for (the more expensive) vibronic calculations. The solvent was modelled through the well-known Polarizable Continuum Mode (PCM),^56^ selecting chloroform. The character of the stationary points on the potential energy surface has been confirmed with the harmonic vibrational analysis. The rotation barriers have been estimated by the optimization of the geometry of the transition state for the phenyl ring rotation around the C15–C17 bond (for atom numbering see Fig. S52†). All these calculations have been carried out with Gaussian16.^57^ The SOC matrix elements were determined at the M06-2X/def2-TZVP level^58^ using ORCA.5.0.1 (ref. 59) with CHCl_3_ as the solvent as modelled with the SMD solvation model. The RIJCOSX method was used to accelerate the calculation, and DefGrid3 and TightSCF settings were applied, whereas TDA was turned off. The S–T gaps were evaluated using SCS-CC2/aug-cc-pVTZ (in the gas phase)^60,61^ because this method performs especially well for these gaps.^62^ These SCS-CC2 calculations were performed with Turbomole 7.3.^63^ Given that ISC typically occurs after geometry relaxation in organic dyes (free of heavy elements), these calculations have been performed on the ES minima. For the MECP search, we used the Q-Chem 5.4 code,^64^ applying the same level of theory as above, that is PCM-MN15/6-311+G(d,p), using the spin-flip approach. We considered four roots, applied the branching plane approach to locate the MECP, and searched for the MECP between the two lowest singlet states, defined with a spin-contamination threshold of 1.2. Default Q-Chem parameters were otherwise applied. In practice the MECP search was first performed with the non-substituted 5, exploring a large number of possible deformations as the starting point (kinked structures twisting various moieties, hydrogen atoms moved out of plane, elongated bonds, *etc.*). It is indeed our experience that starting from the FC geometry does not necessarily yield the lowest MECP, especially for quasiplanar systems. This allowed the determination of the most accessible MECP. Starting from there, the MECPs of all other compounds were searched for. The energy difference between the MECP and the excited-state minimum was determined through PCM-SF-TD-MN15/6-311+G(d,p) single point calculations performed on the optimal excited-state structure found by conventional TD-DFT (see above) and on the SF-TD-DFT optimized MECP. All the vibronic calculations have been carried out using FCClasses 3 (v3-0.1-216-g29ba701).^65^ Both the adiabatic and vertical Hessian PES approximations (AH and VH, respectively) have been originally used along with the Time-Dependent (TD)^66^ and Time-Independent (TI)^67,68^ formalisms (see also below). Considering the large oscillator strength values (*f* > 0.5, typically *ca.* 0.8–1.2), the Herzberg–Teller effects were neglected. We used geometries, gradients, and Hessians computed at the PCM-MN15/6-311+G(d,p) level, and further corrected the energies and transition dipole moment using gas-phase CC2/aug-cc-pVTZ results. These CC2 calculations were performed in an effort to improve the accuracy and were made with Turbomole. Regarding the pre-screening parameters of FCClasses we used *C*^max^_1_ = 30, *C*^max^_2_ = 25 for the maximum number of integrals in both *C*_1_ and *C*_2_ classes and we set the threshold to select the thermally excited initial states to 0.8. The number of maximum integrals was set to 10^6^ for the routine TI calculation. All the emission spectra were convoluted with a Gaussian broadening of 450 cm^−1^ to ensure matching with experimental data. The Internal Conversion (IC) spectra use a 10 cm^−1^ Lorentzian broadening, a typical choice.^69^ In order to select the appropriate vibronic model we carried out some tests on 4, H/OMe (the corresponding spectra shown in the ESI†). Since a better convergence of the TI spectra was obtained within the AH approximation, as well as the expected shape of the *k*_IC_*versus* energy plot, we pursued the work using TD AH|FC formalism, which we report in the body of the text.

## Data availability

Additional computational data are available upon request to DJ while experimental ones from BO.

## Author contributions

PR: investigation, formal analysis, validation, visualization, writing – original draft preparation; MHEB: investigation, formal analysis, validation, visualization, writing – original draft preparation; AKK: investigation, visualization, writing – original draft preparation; BJ: investigation, formal analysis, writing – review & editing; DJ: conceptualization, methodology, resources, writing – original draft preparation, supervision; BO: conceptualization, project administration, methodology, resources, writing – original draft preparation, supervision.

## Conflicts of interest

There are no conflicts to declare.

## Supplementary Material

SC-013-D2SC05044G-s001
