# Supplementary material for: Controlling the fluorescence quantum yields of benzothiazole-difluoroborates by optimal substitution
Source: Chem Sci. 2022 Oct 24;13(45):13347–60. doi: 10.1039/d2sc05044g (PMC9682896; doi:10.1039/d2sc05044g)
Supplement: SC-013-D2SC05044G-s001 [file SC-013-D2SC05044G-s001.pdf]

Electronic Supplementary Material (ESI) for Chemical Science.
This journal is © The Royal Society of Chemistry 2022

Supporting Information: Controlling the quantum yields of benzothiazole-difluoroborates by optimal substitution

Patryk Rybczyński,† Manon H. E. Bousquet,‡ Anna Kaczmarek-Kędziera,† Beata Jędrzejewska,¶ Denis Jacquemin,*,‡,§ and Borys Ośmiałowski*,†

†*Faculty of Chemistry, Nicolaus Copernicus University in Toruń, Gagarina Street 7, 87-100 Toruń, Poland*

‡*Universite de Nantes, CNRS, CEISAM UMR 6230, F-44000 Nantes, France*

¶*Bydgoszcz University of Science and Technology, Faculty of Chemical Technology and Engineering, Seminaryjna 3, 85-326 Bydgoszcz, Poland*

§*Institut Universitaire de France, F-75005 Paris, France*

E-mail: Denis.Jacquemin@univ-nantes.fr; borys.osmialowski@umk.pl

October 18, 2022

Additional experimental data.

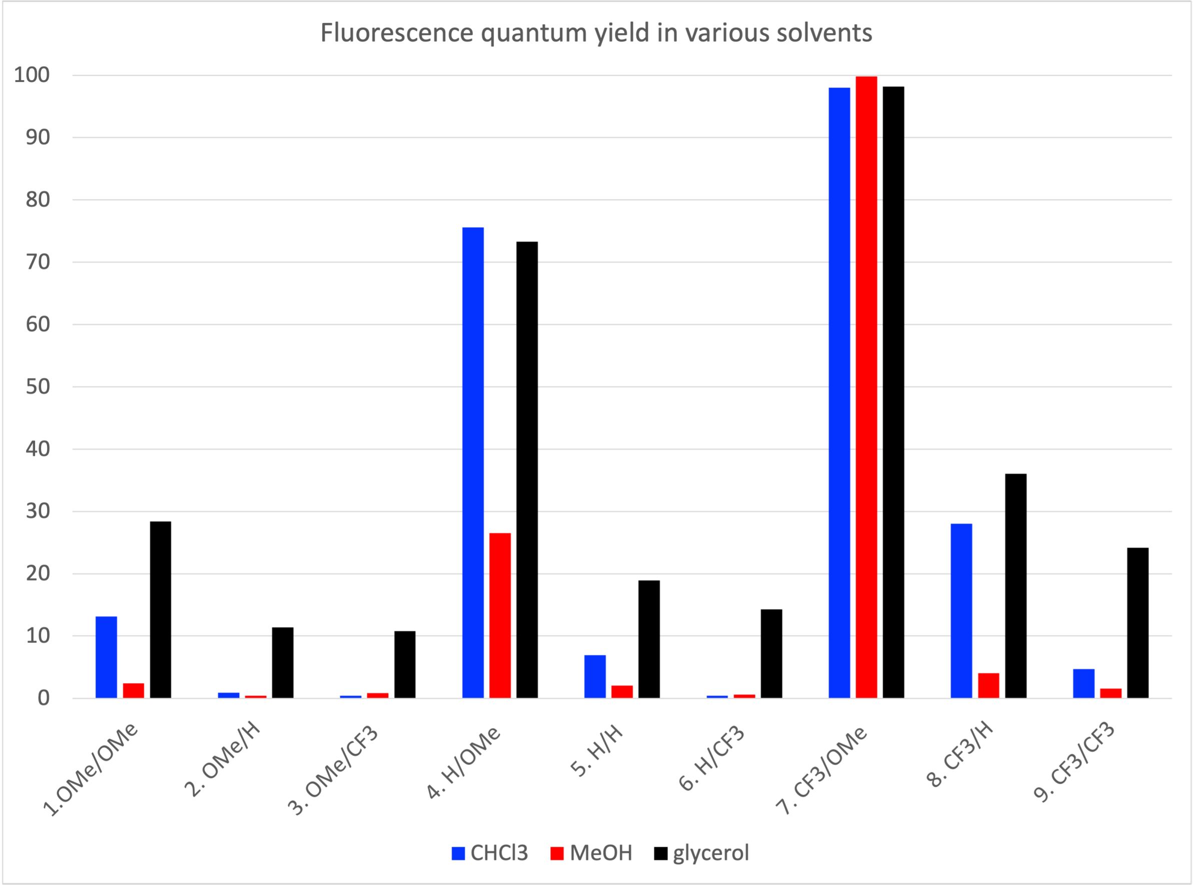

Figure S1: Fluorescence quantum yield (in %) for all dyes in various media.

Table S1: Fluorescence quantum yield (%), lifetime of fluorescence (ns), radiative and non-radiative rate constants ($10^9 s^{-1}$) measured for all dyes in three solvents.

	Chloroform				Methanol				Glycerol			
Compound (R/R')	ϕ_f	τ	k_r	k_{nr}	ϕ_f	τ	k_r	k_{nr}	ϕ_f	τ	k_r	k_{nr}
**1** (OMe/OMe)	13.1	0.46	0.28	1.89	2.4	0.14	0.18	7.23	28.4	1.15	0.25	0.62
**2** (OMe/H)	0.9	0.13	0.07	7.87	0.4	0.12	0.04	8.45	11.4	1.08	0.11	0.82
**3** (OMe/CF_3)	0.4	0.10	0.04	10.48	0.8	n.d.	n.d.	n.d.	10.8	0.84	0.13	1.07
**4** (H/OMe)	75.6	1.71	0.44	0.14	26.5	0.68	0.39	1.08	73.3	1.58	0.46	0.17
**5** (H/H)	6.9	0.21	0.34	4.54	n.d.	n.d.	n.d.	n.d.	18.9	0.81	0.23	1.00
**6** (H/CF_3)	0.4	0.13	0.03	7.49	0.6	n.d.	n.d.	n.d.	14.3	0.78	0.18	1.10
**7** (CF_3/OMe)	98.8	1.65	0.59	0.01	99.6	1.69	0.59	0.01	98.2	1.54	0.64	0.01
**8** (CF_3/H)	28.4	1.02	0.28	0.71	4.0	n.d.	n.d.	n.d.	36.0	1.31	0.28	0.49
**9** (CF_3/CF_3)	4.7	0.23	0.21	4.18	1.6	n.d.	n.d.	n.d.	24.2	1.10	0.22	0.69

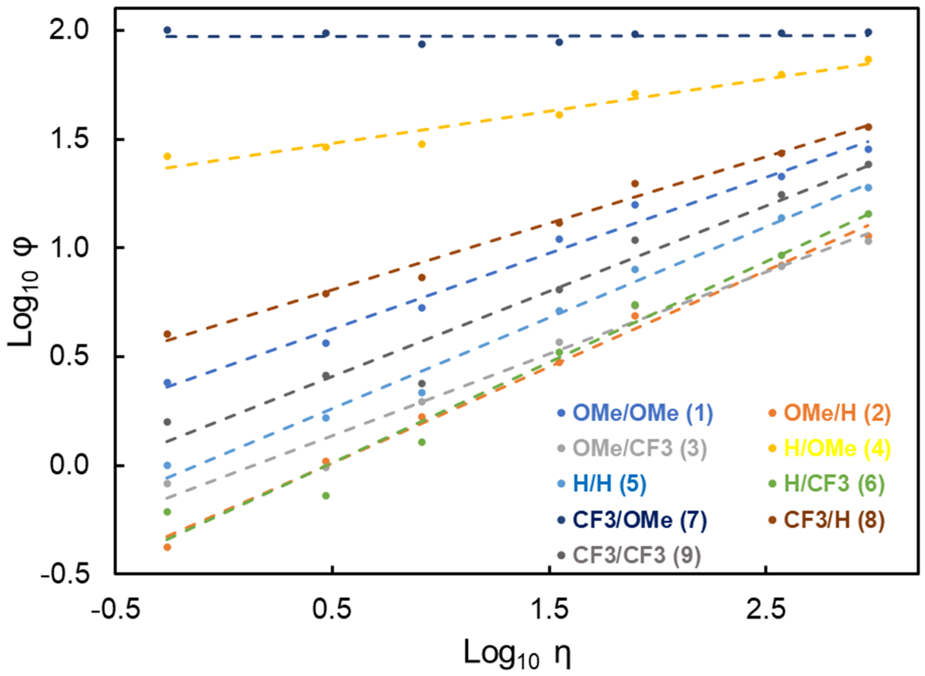

Figure S2: Linear fit for the logarithms of fluorescence quantum yield and solvent viscosity for all compounds

Table S2: Linear correlation parameters for linear fit on Fig. S2

Compound	1	2	3	4	5	6	7	8	9
a	2.81	2.25	2.59	6.44	2.23	2.10	0.11	3.22	2.44
b	-1.24	0.48	0.16	-8.99	0.025	0.50	1.22	-2.09	-0.46
R^2	0.983	0.994	0.973	0.952	0.988	0.971	0.000	0.987	0.959

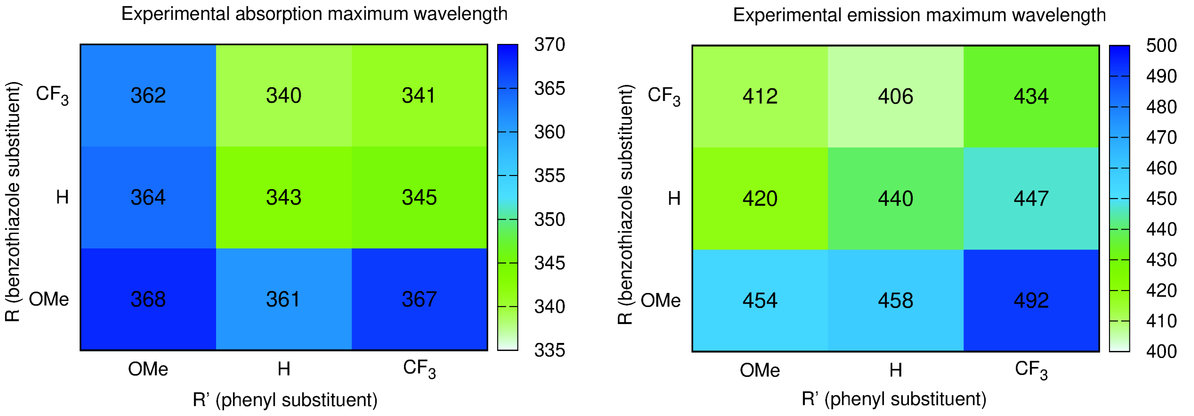

Figure S3: Experimental absorption and emission wavelengths (nm) measured in chloroform.

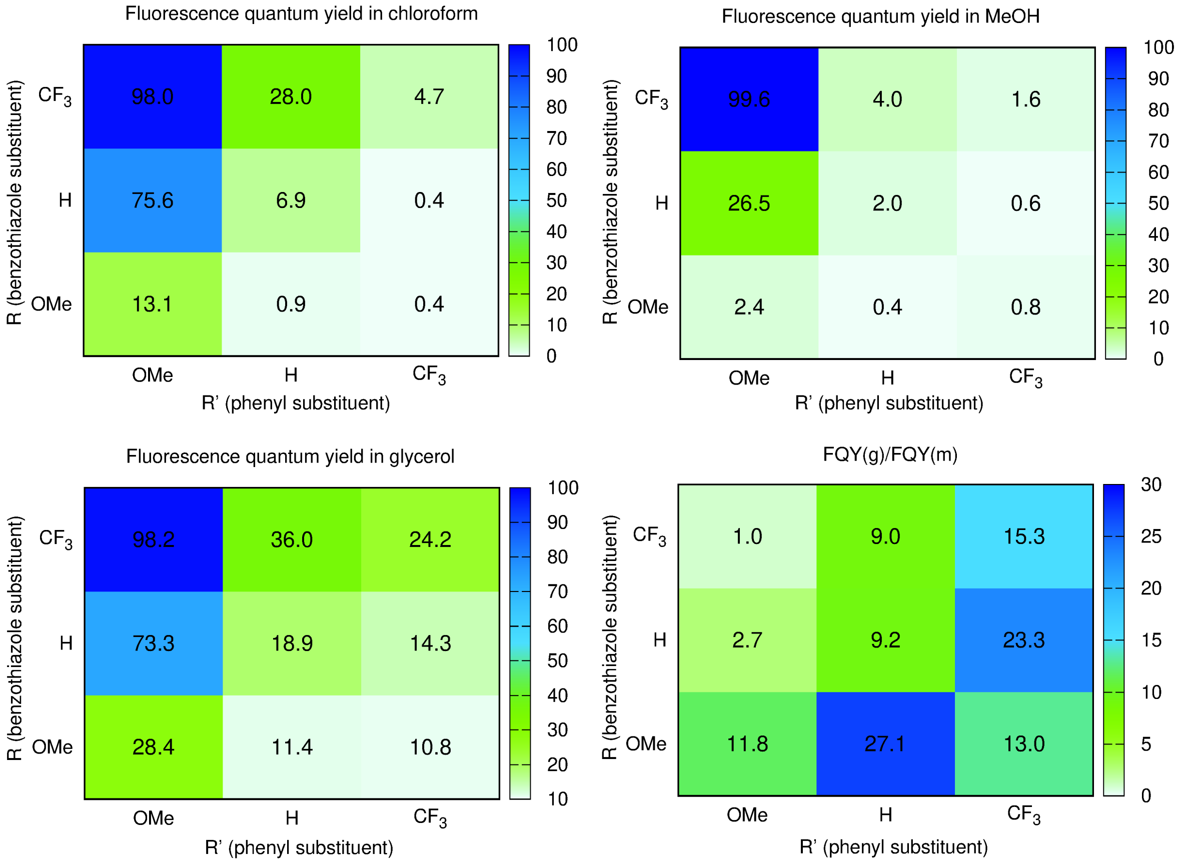

Figure S4: Measured fluorescence quantum yield in chloroform, methanol and glycerol (%), and the ratio of the fluorescence quantum yields in the two latter media, $\frac{\Phi_F(g.)}{\Phi_F(m.)}$

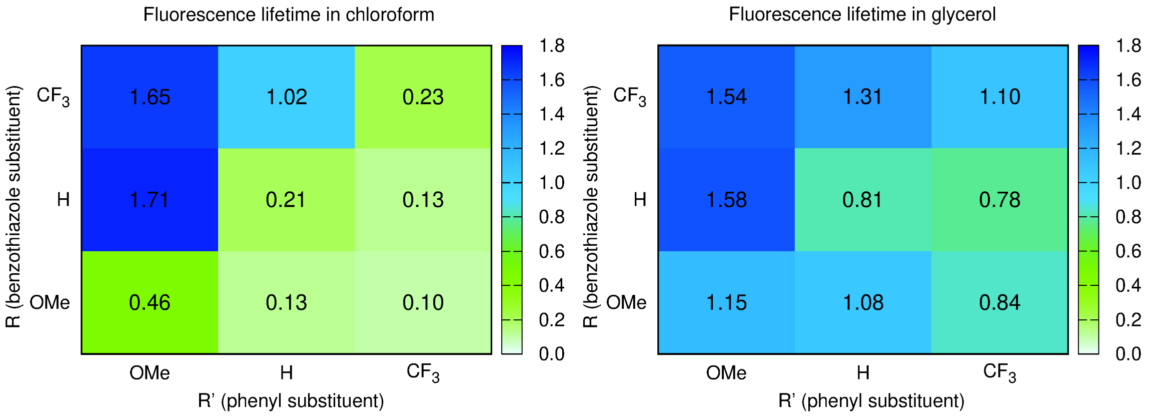

Figure S5: Experimental fluorescence lifetime (ns) measured in chloroform and glycerol.

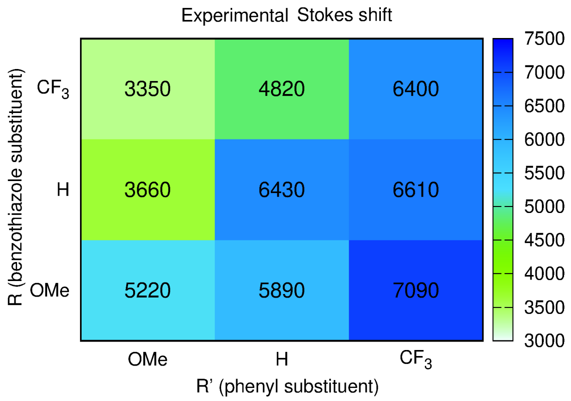

Figure S6: Experimental Stokes shift (cm^{-1}) in chloroform.

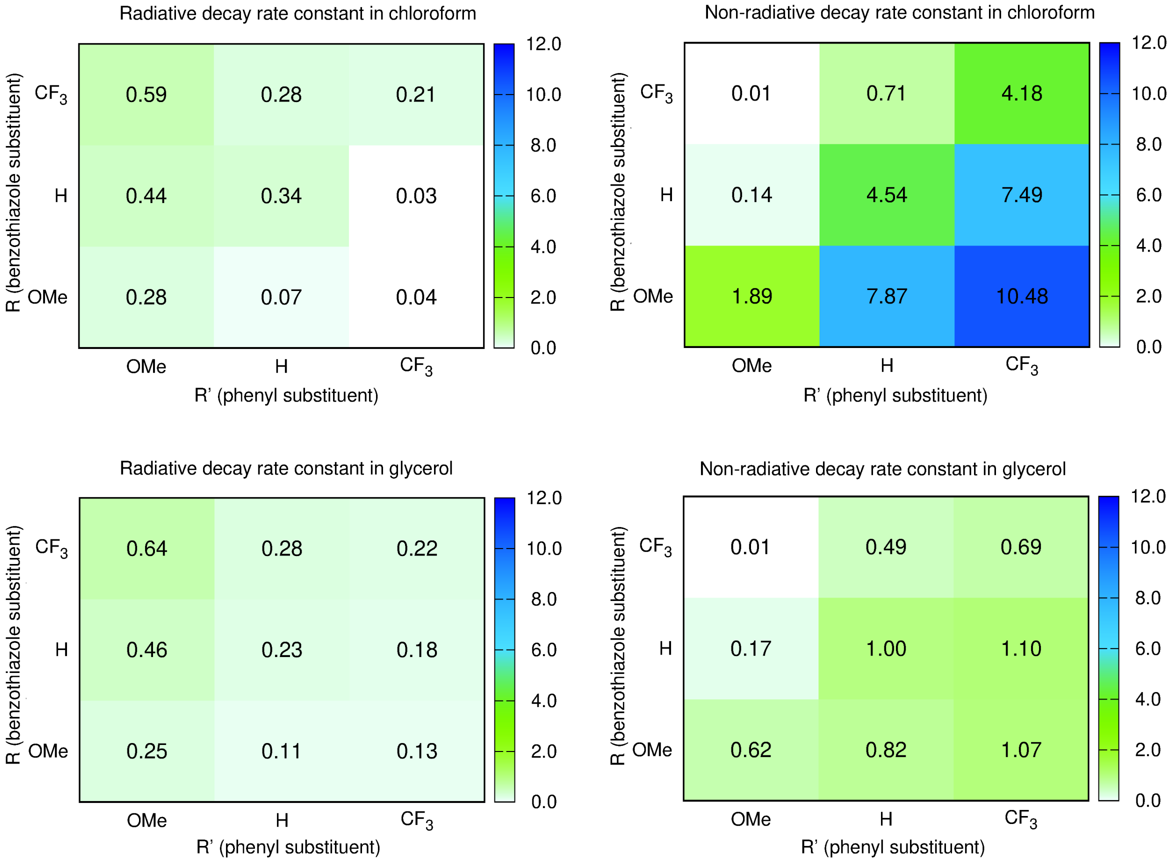

Figure S7: Rate constants for radiative and non-radiative (10^9 s^{-1}) decay in chloroform and in glycerol.

NMR and mass spectra

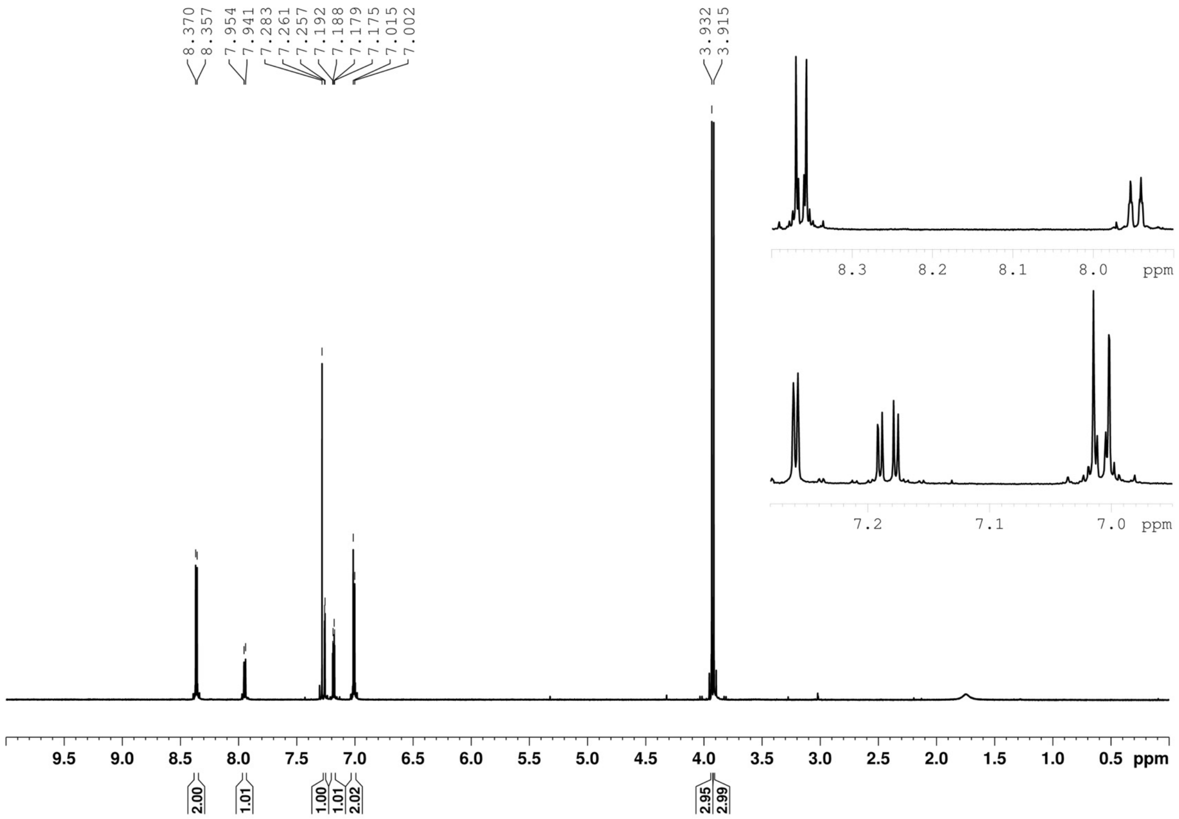

Figure S8: The ^{1}NMR spectrum of compound **1**.

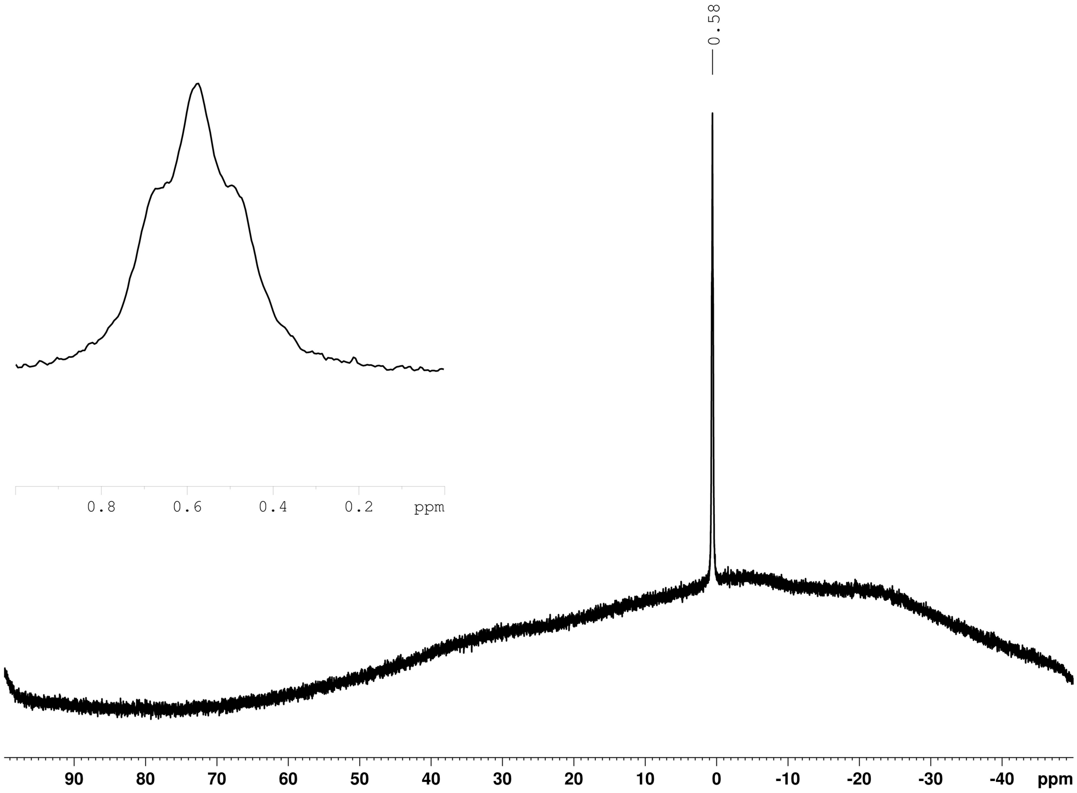

Figure S9: The ^{11}B NMR spectrum of compound **1**.

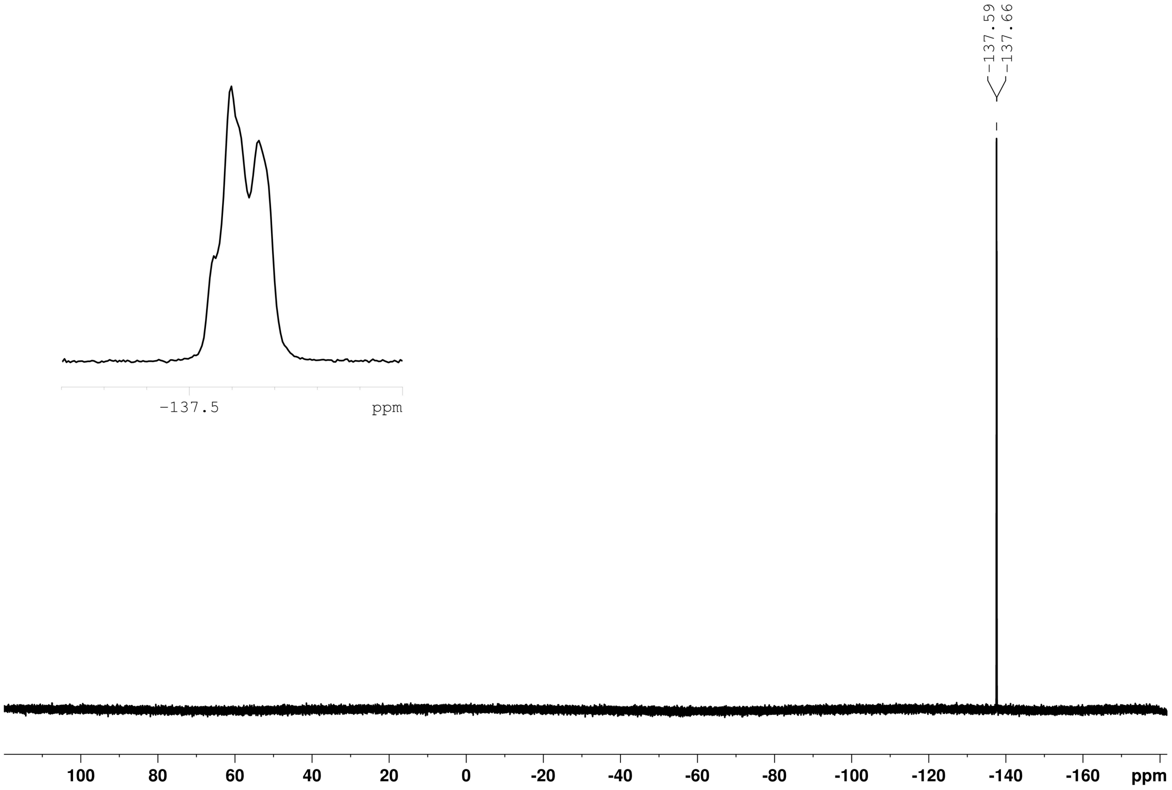

Figure S10: The ^{19}F NMR spectrum of compound **1**.

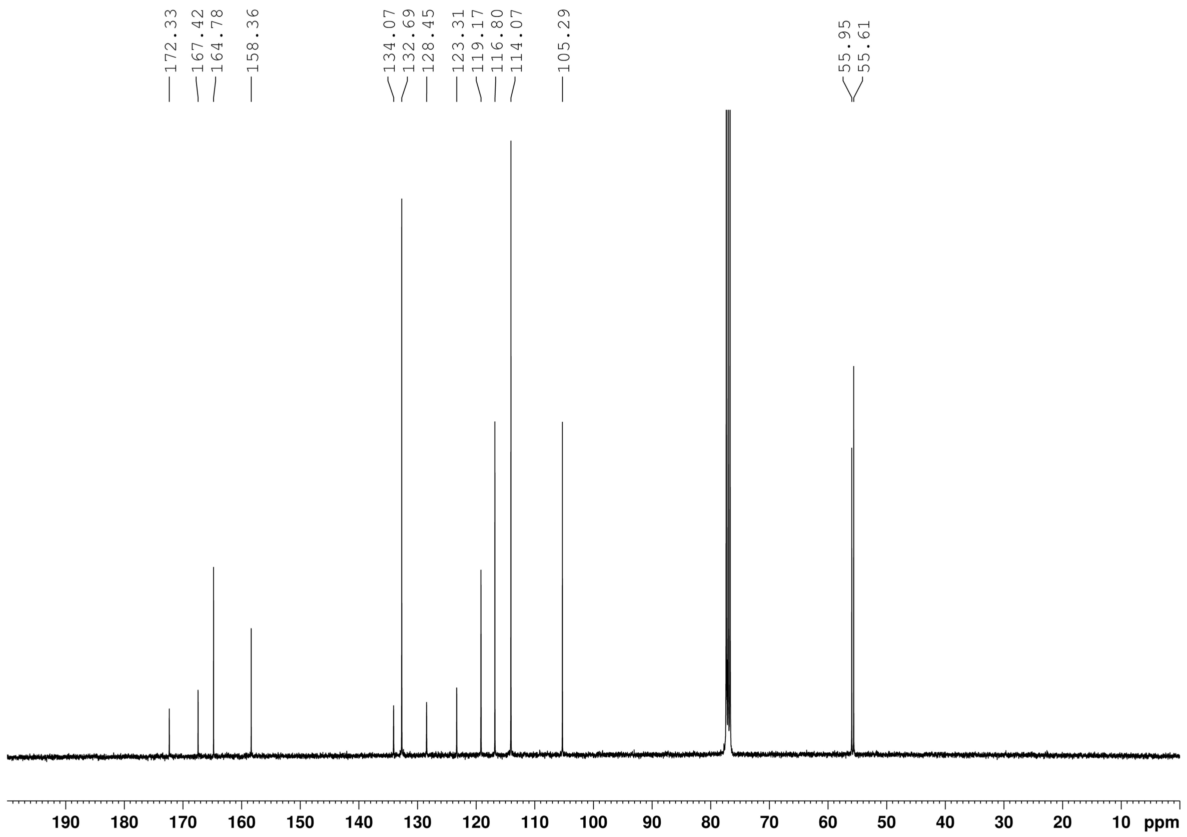

Figure S11: The ^{13}C NMR spectrum of compound **1**.

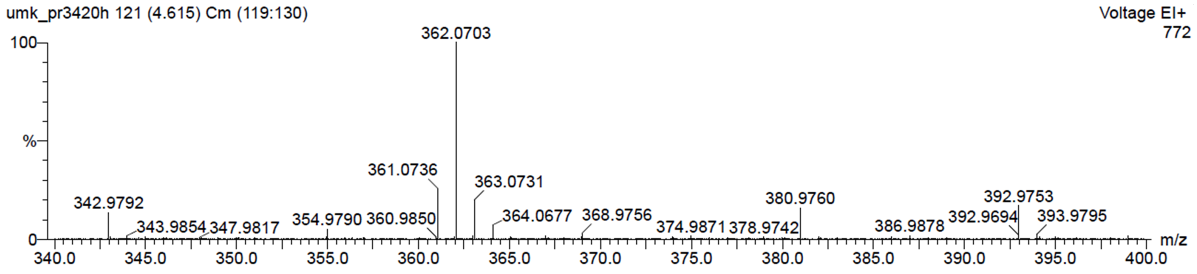

Figure S12: The MS spectrum of compound **1**.

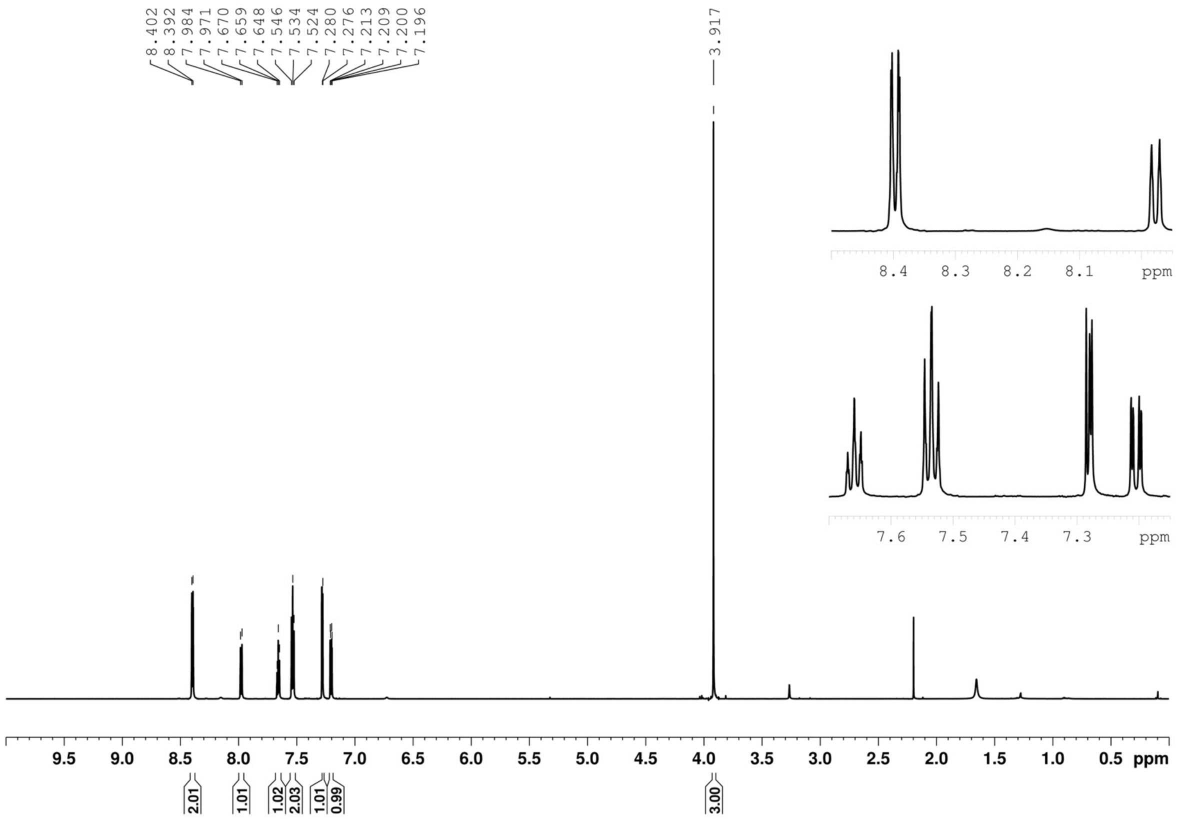

Figure S13: The [1]NMR spectrum of compound **2**.

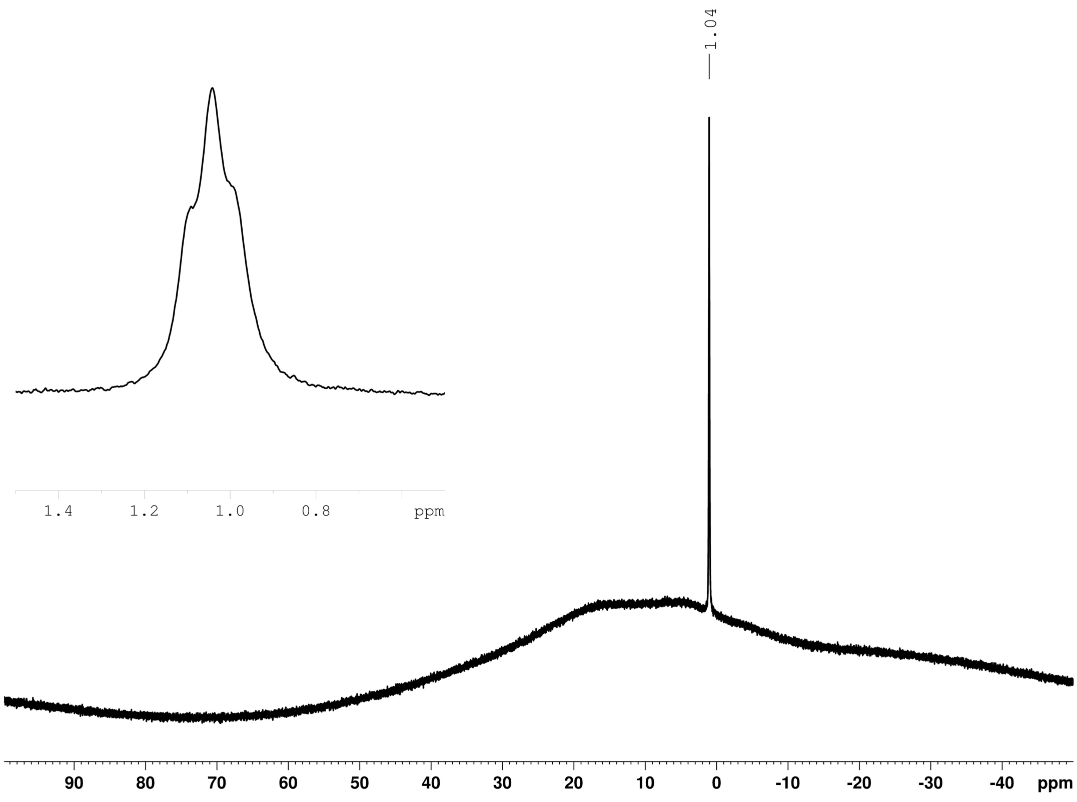

Figure S14: The ^{11}B spectrum of compound **2**.

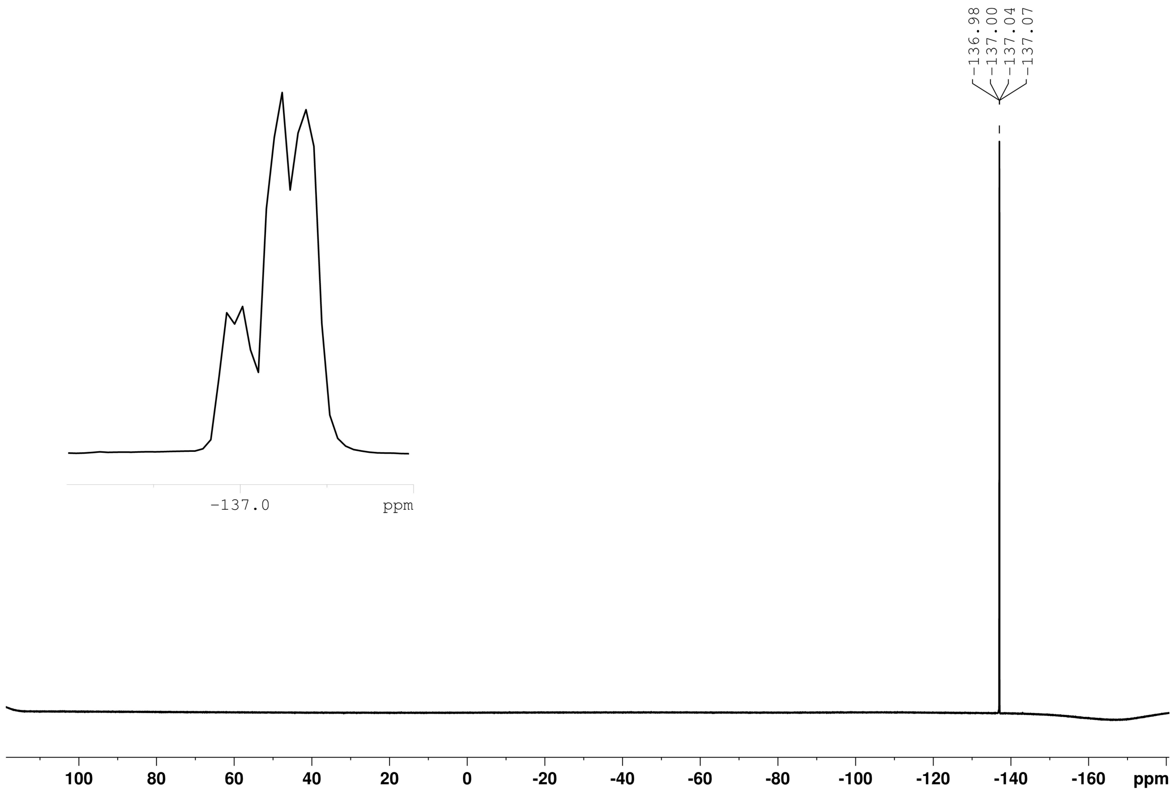

Figure S15: The ^{19}F NMR spectrum of compound **2**.

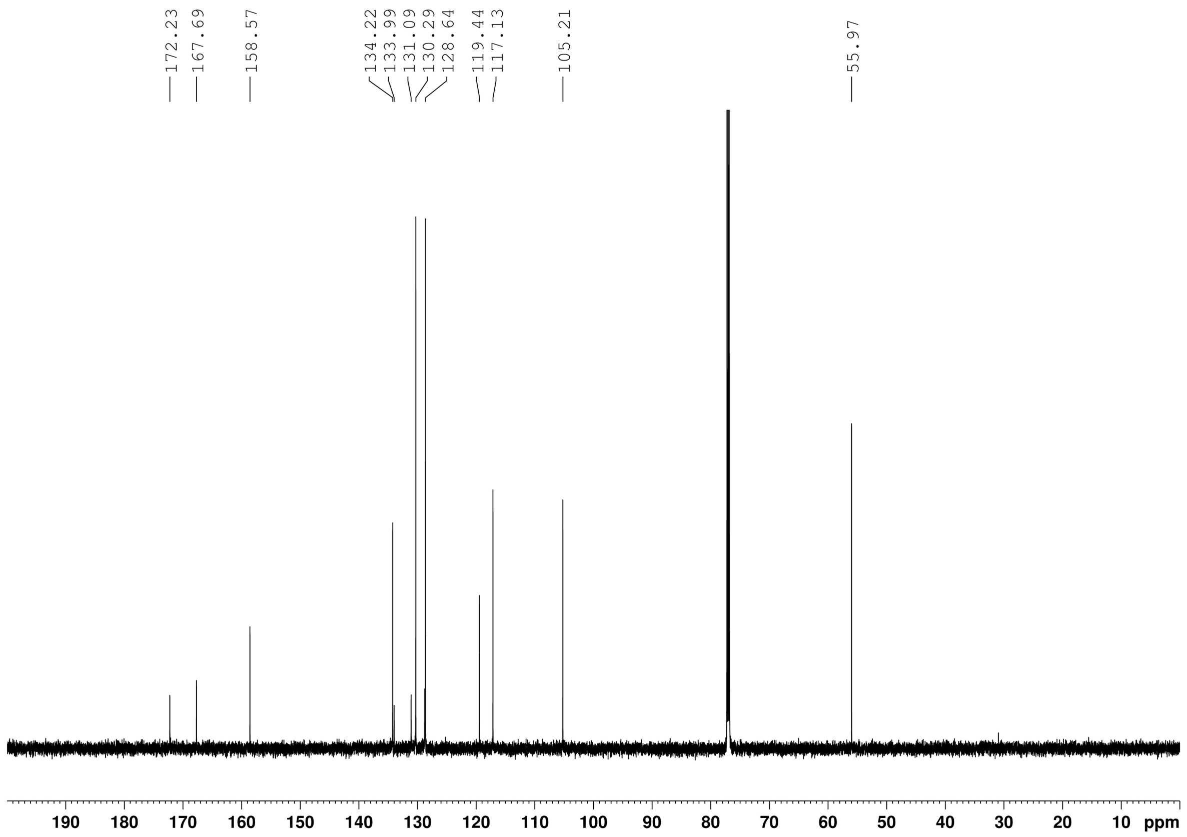

Figure S16: The ^{13}C NMR spectrum of compound **2**.

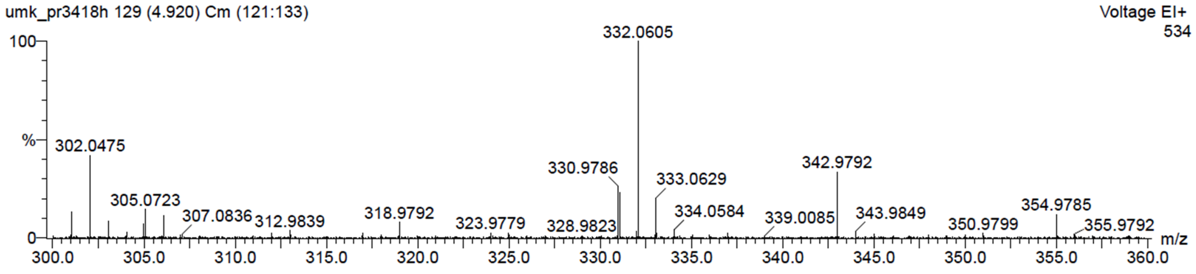

Figure S17: The MS spectrum of compound **2**.

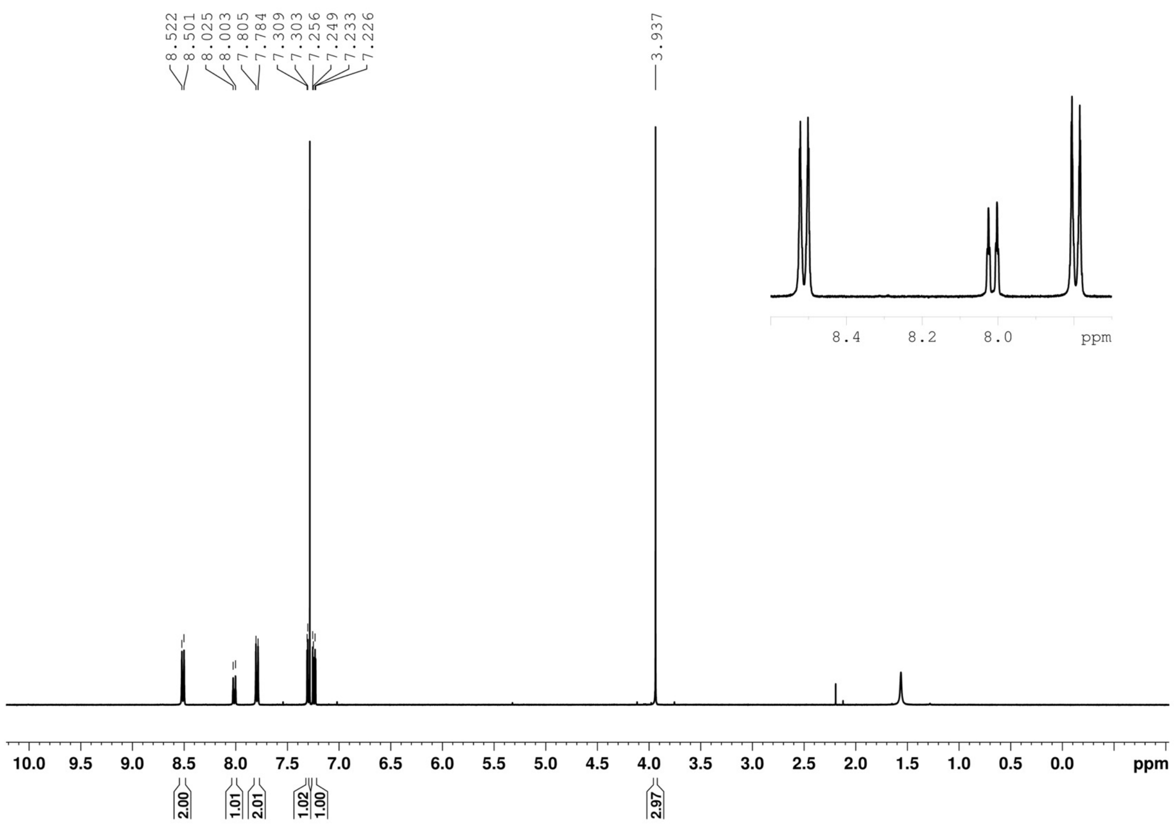

Figure S18: The [1]NMR spectrum of compound **3**.

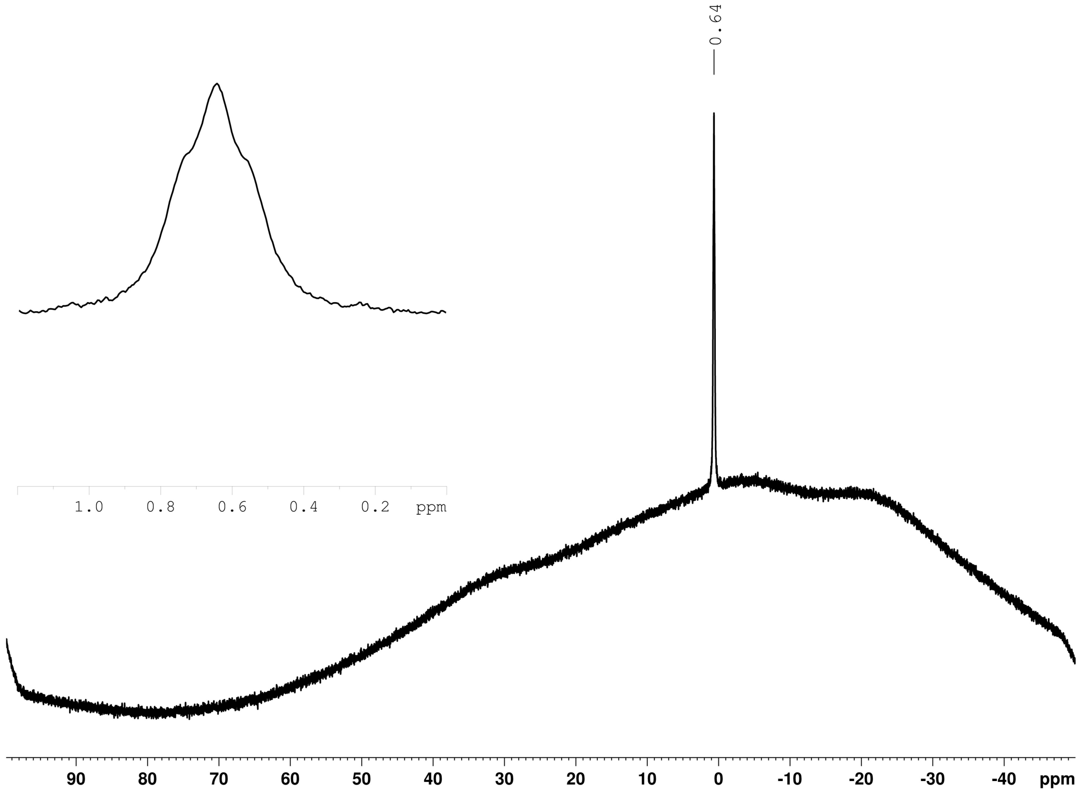

Figure S19: The ^{11}B spectrum of compound **3**.

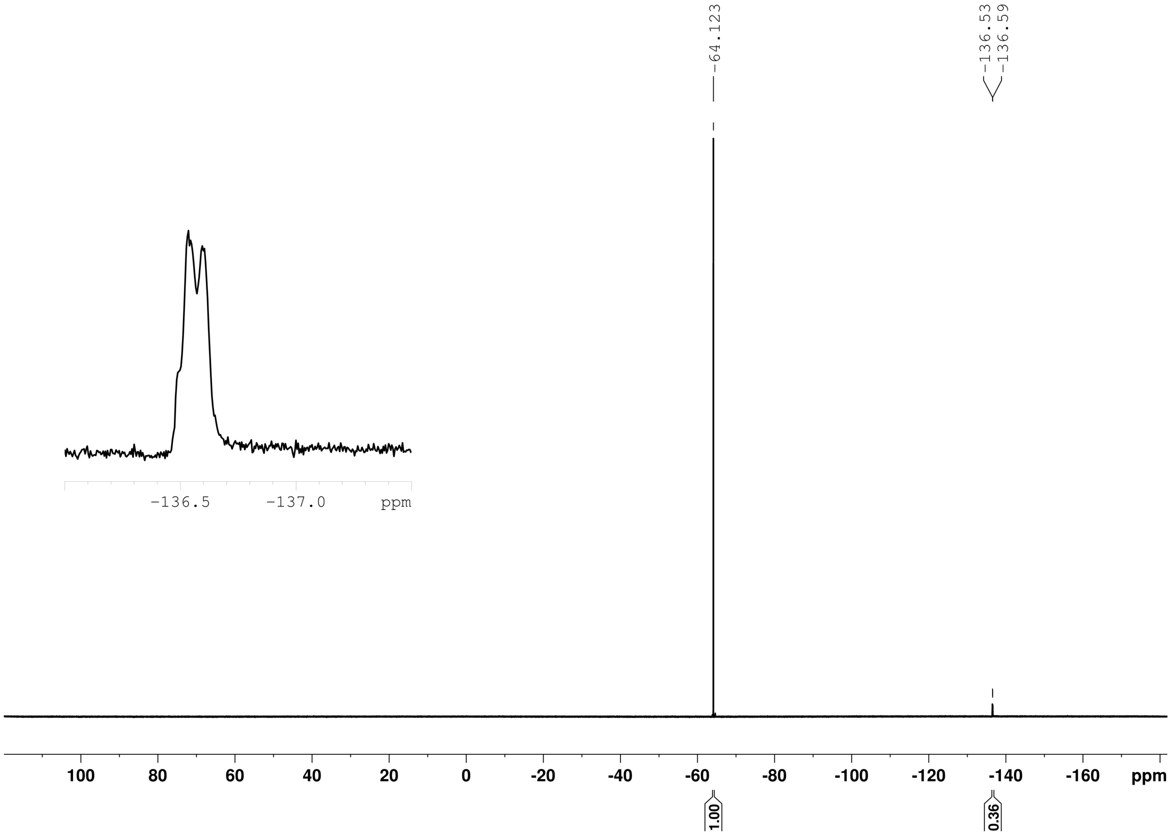

Figure S20: The ^{19}F NMR spectrum of compound **3**.

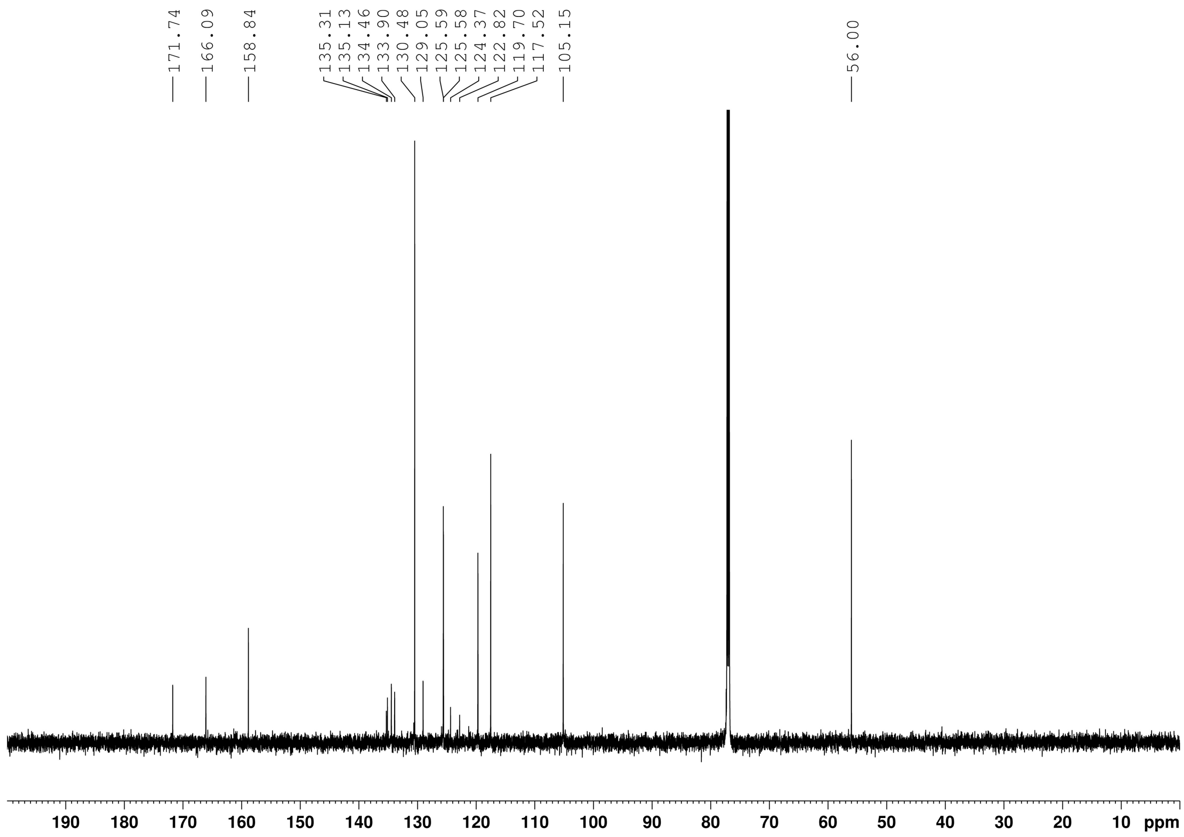

Figure S21: The ^{13}C NMR spectrum of compound **3**.

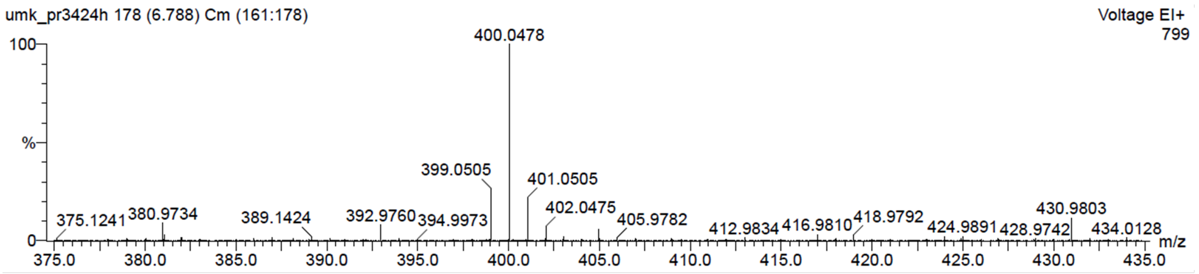

Figure S22: The MS spectrum of compound **3**.

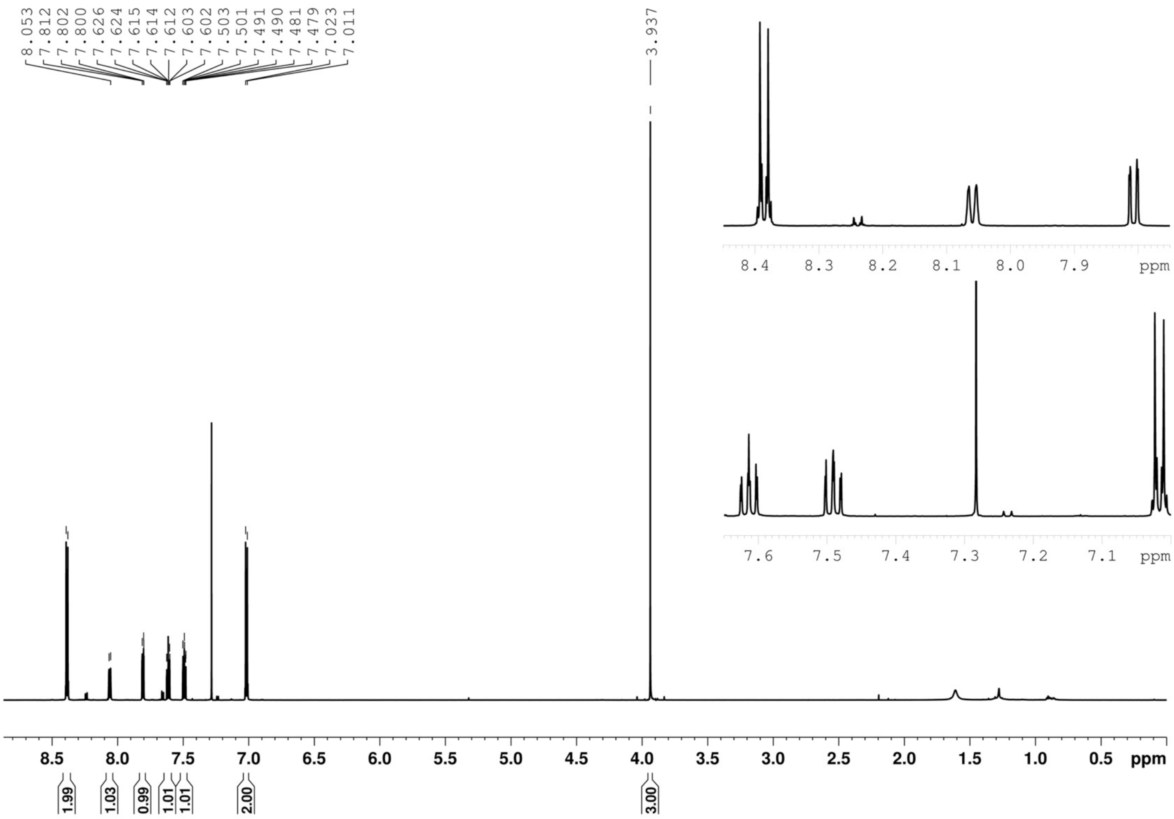

Figure S23: The ^{1}NMR spectrum of compound **4**.

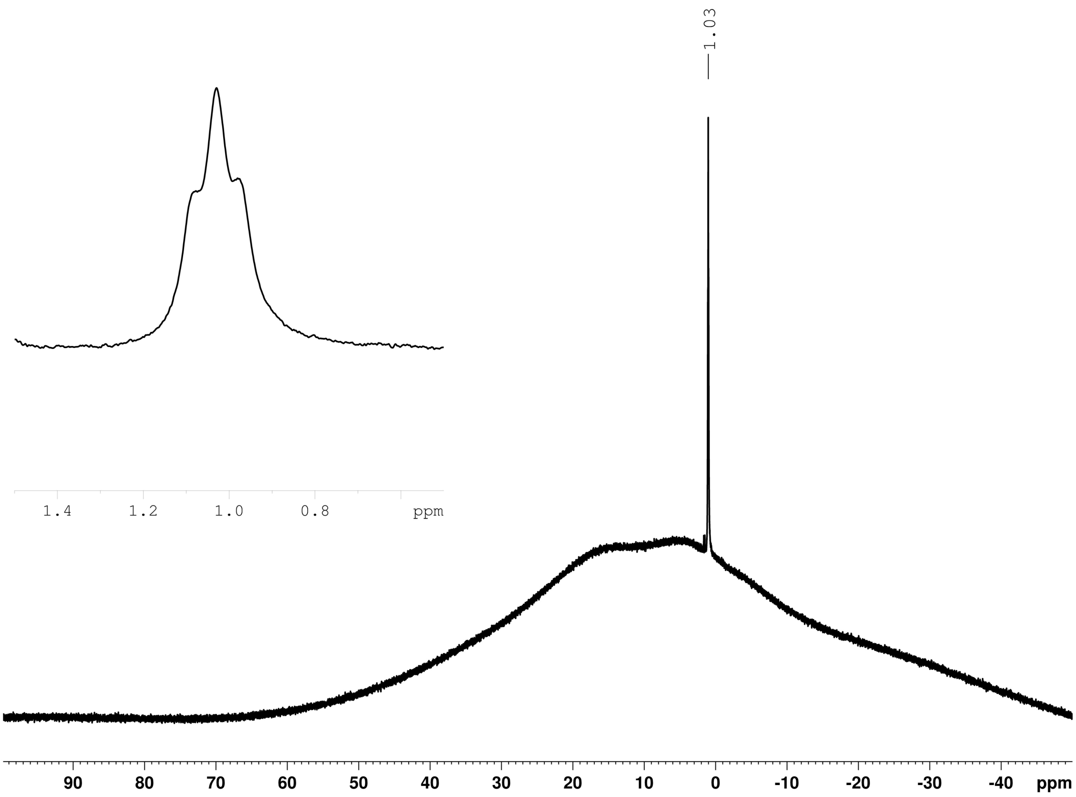

Figure S24: The ^{11}B NMR spectrum of compound **4**.

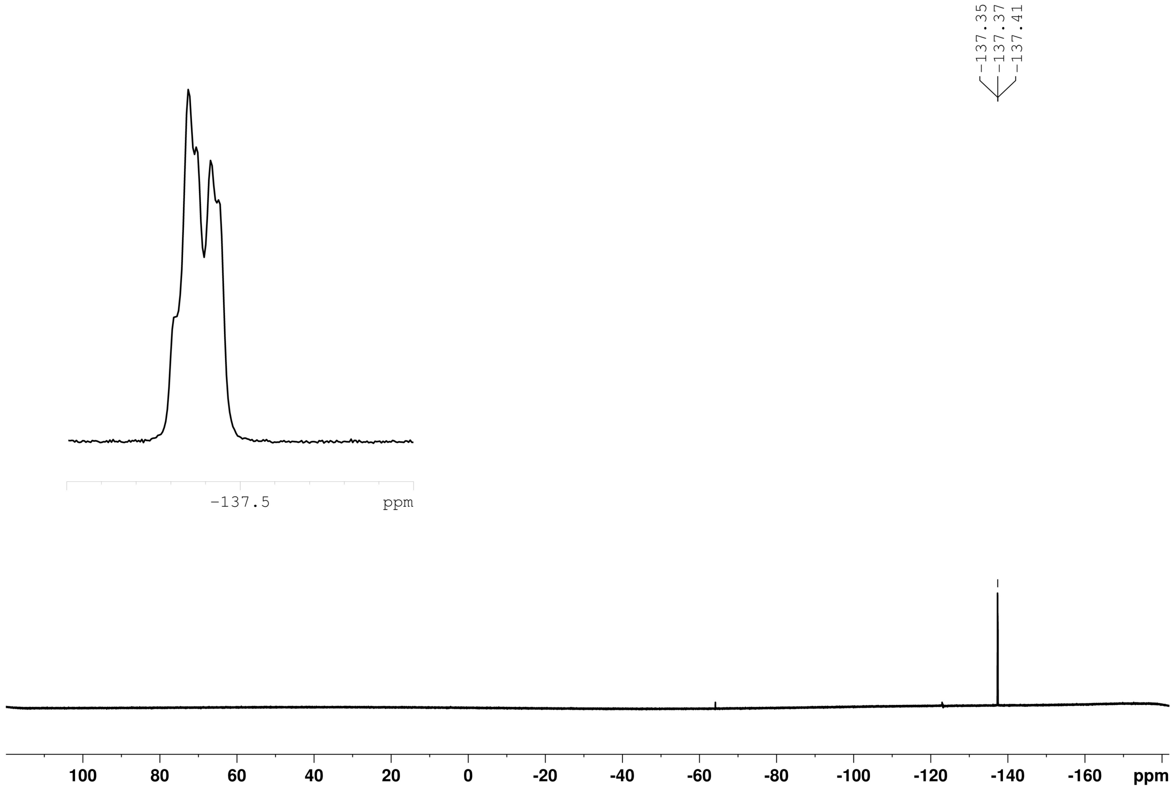

Figure S25: The ^{19}F NMR spectrum of compound **4**.

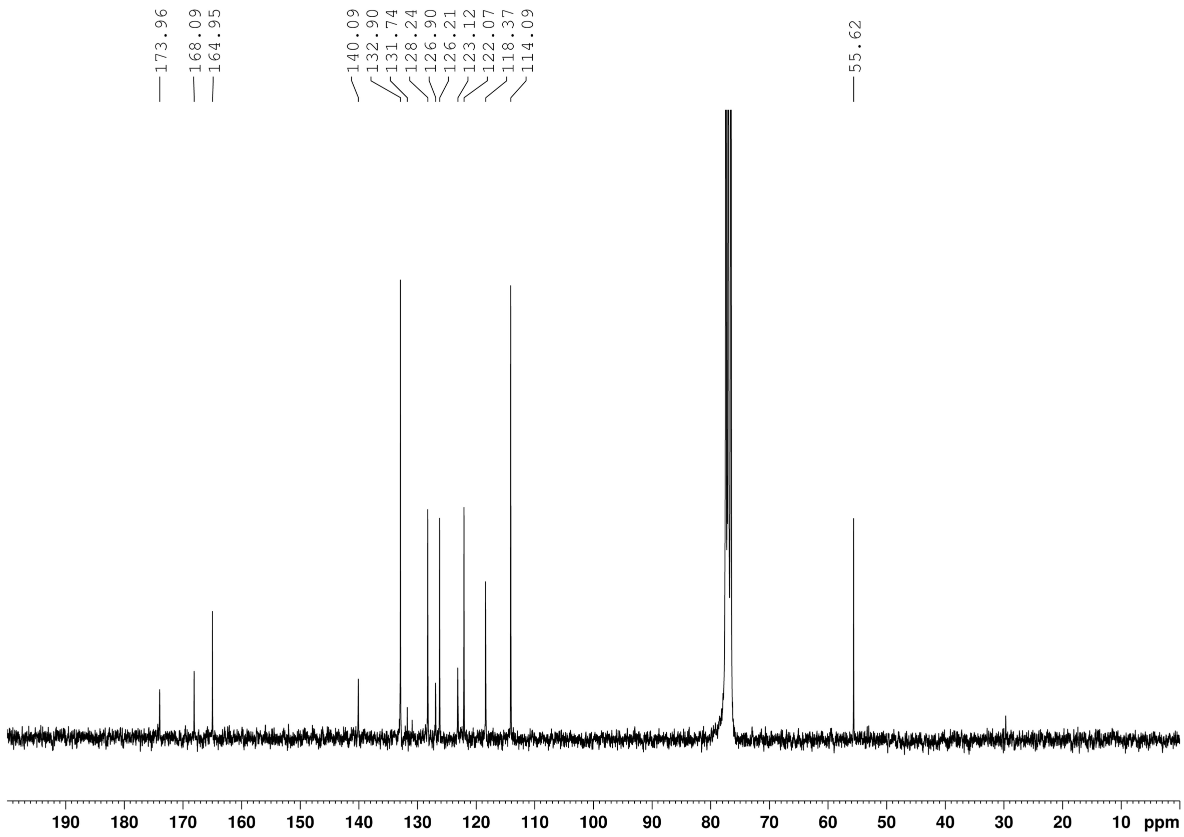

Figure S26: The ^{13}C NMR spectrum of compound **4**.

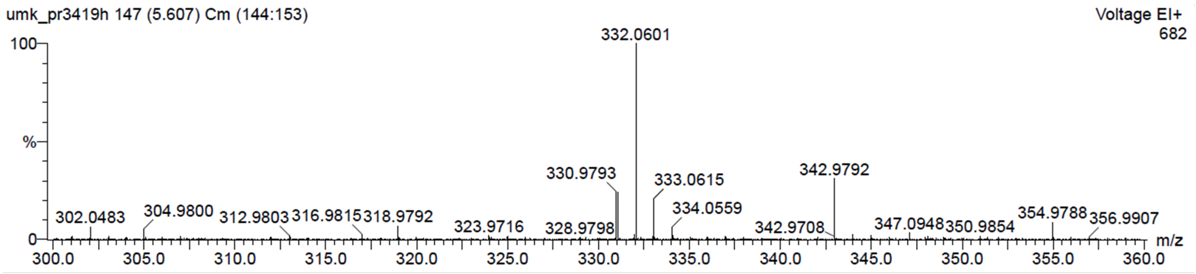

Figure S27: The MS spectrum of compound **4**.

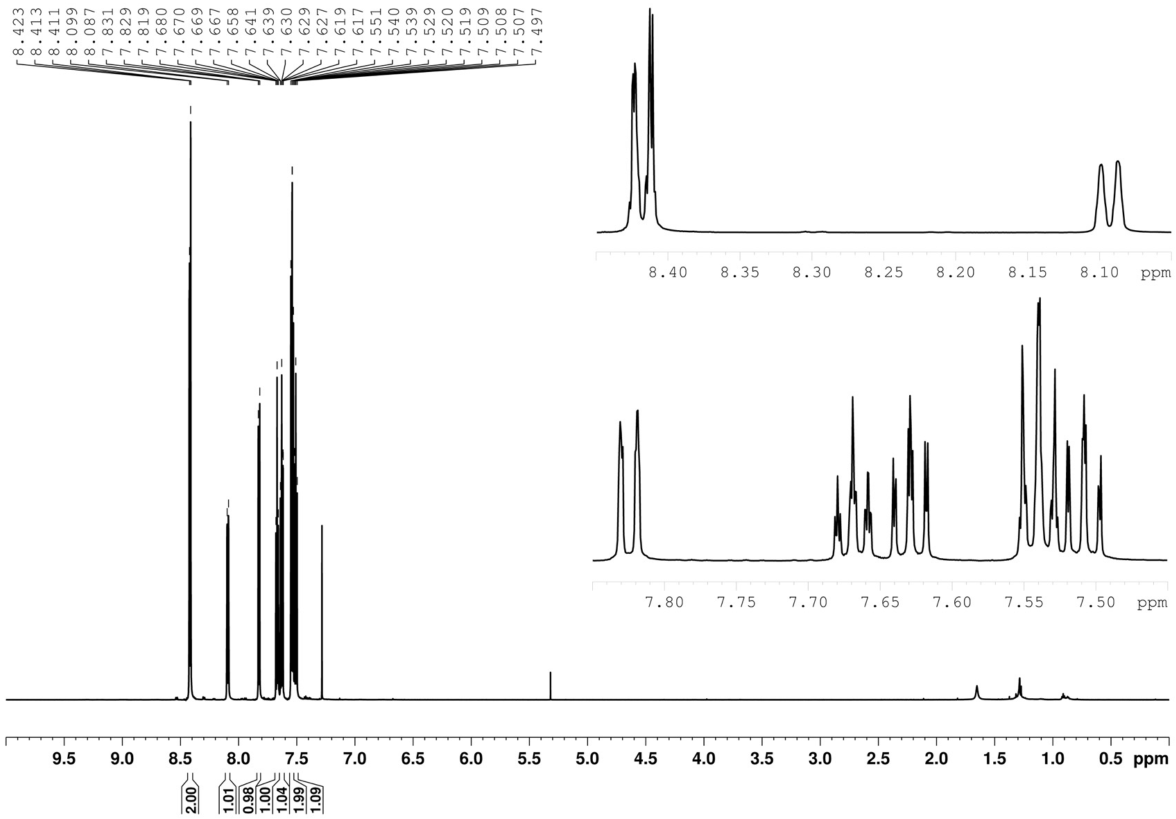

Figure S28: The ^{1}NMR spectrum of compound **5**.

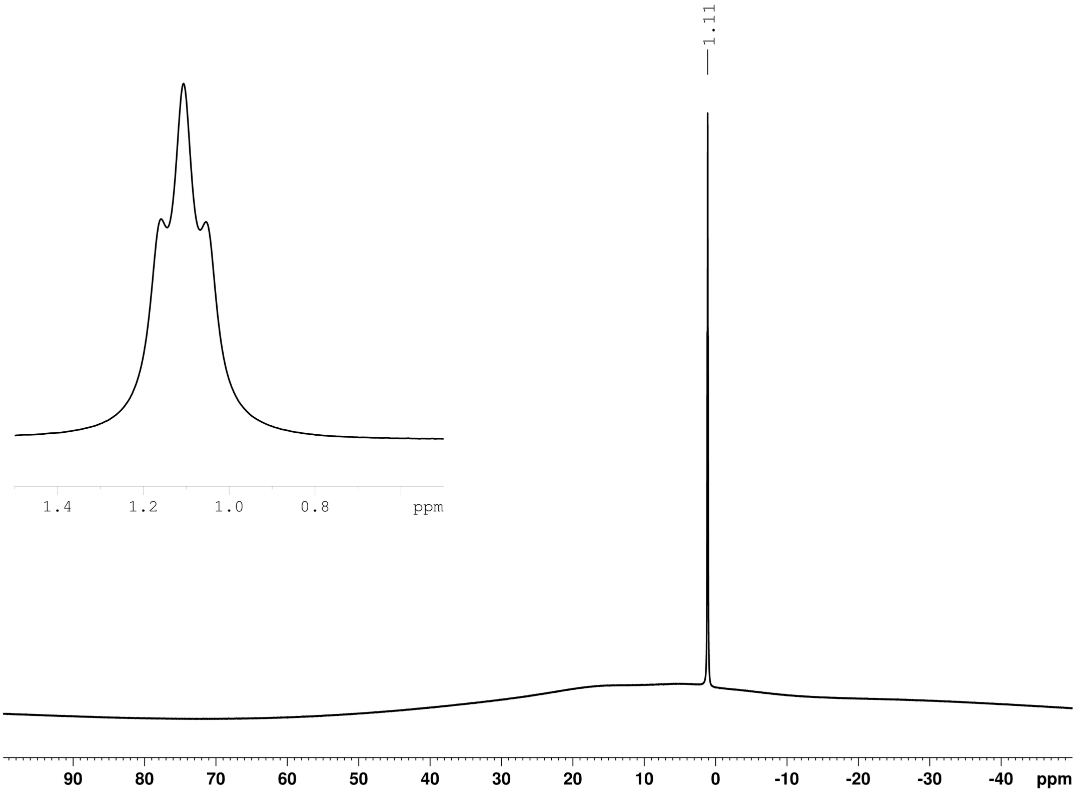

Figure S29: The ^{11}B NMR spectrum of compound **5**.

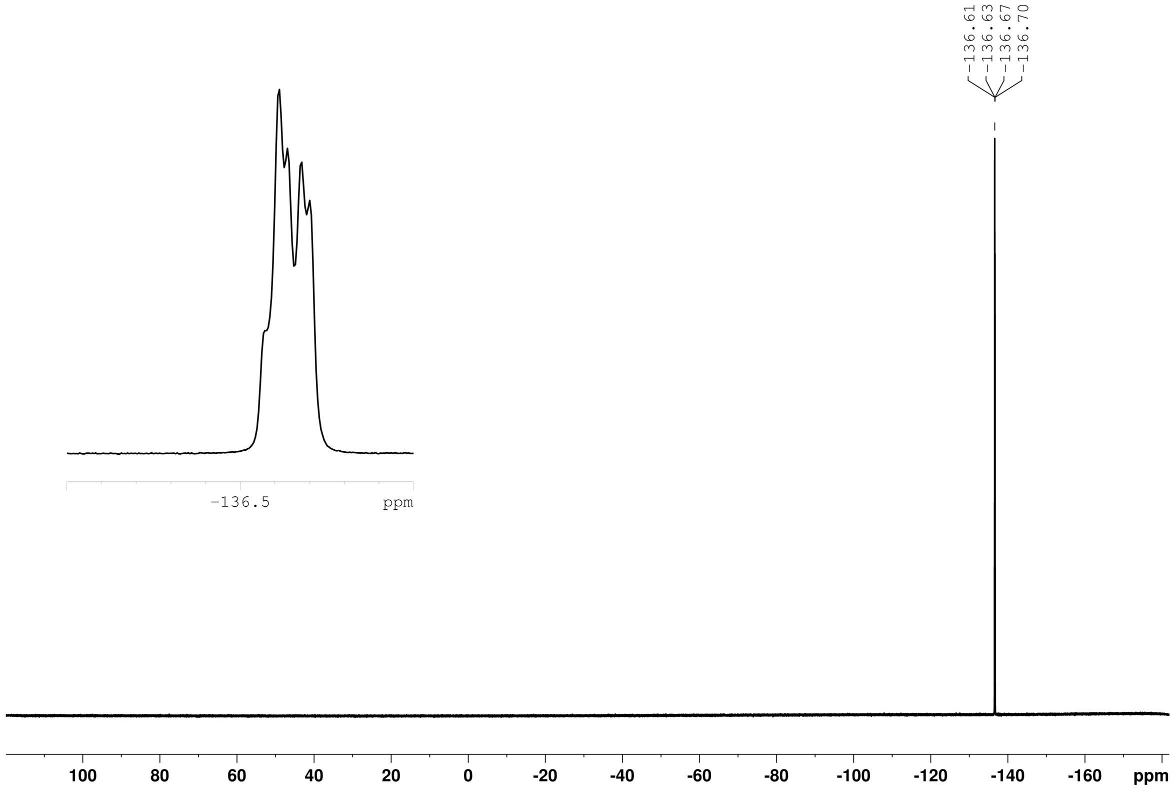

Figure S30: The ^{19}F NMR spectrum of compound **5**.

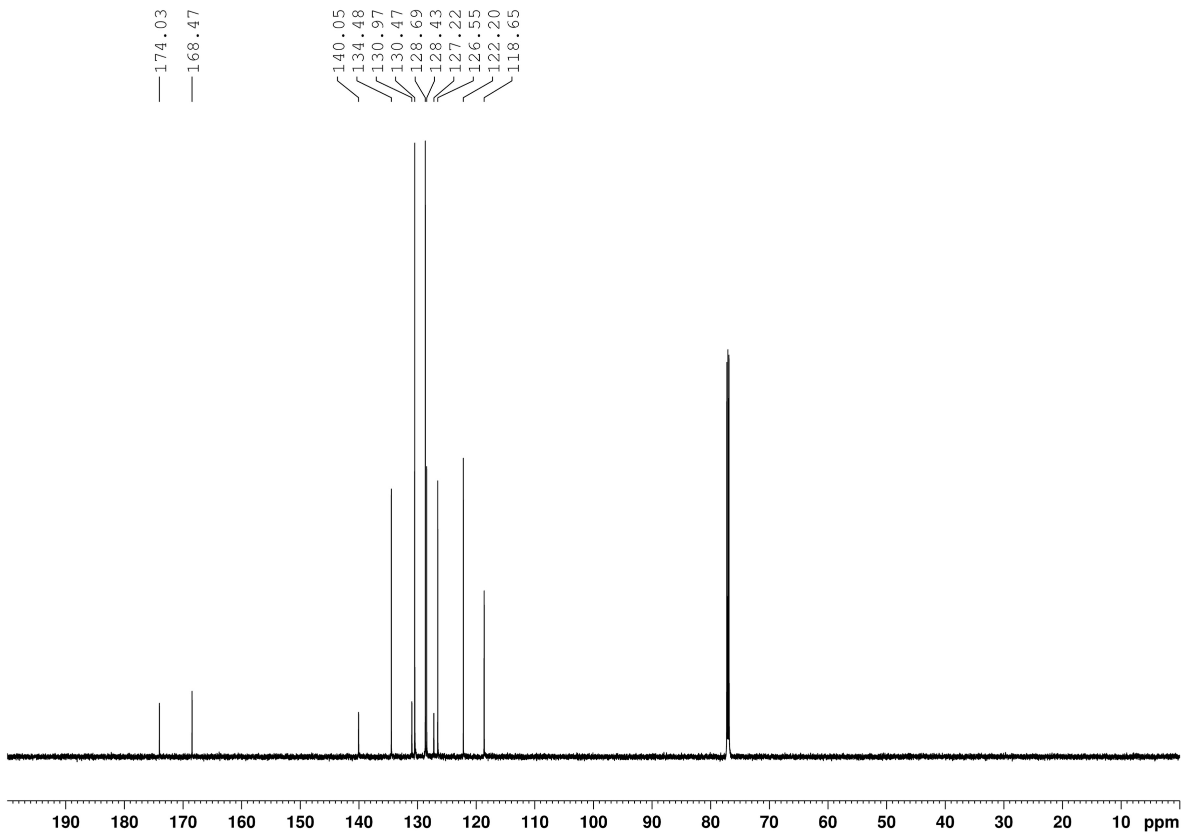

Figure S31: The ^{13}C NMR spectrum of compound **5**.

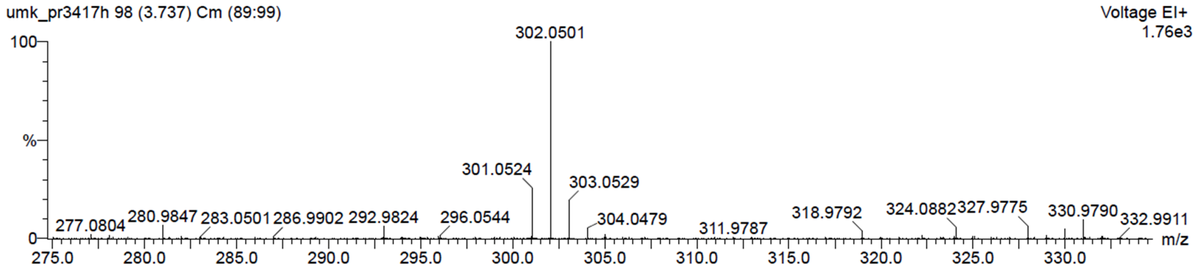

Figure S32: The MS spectrum of compound **5**.

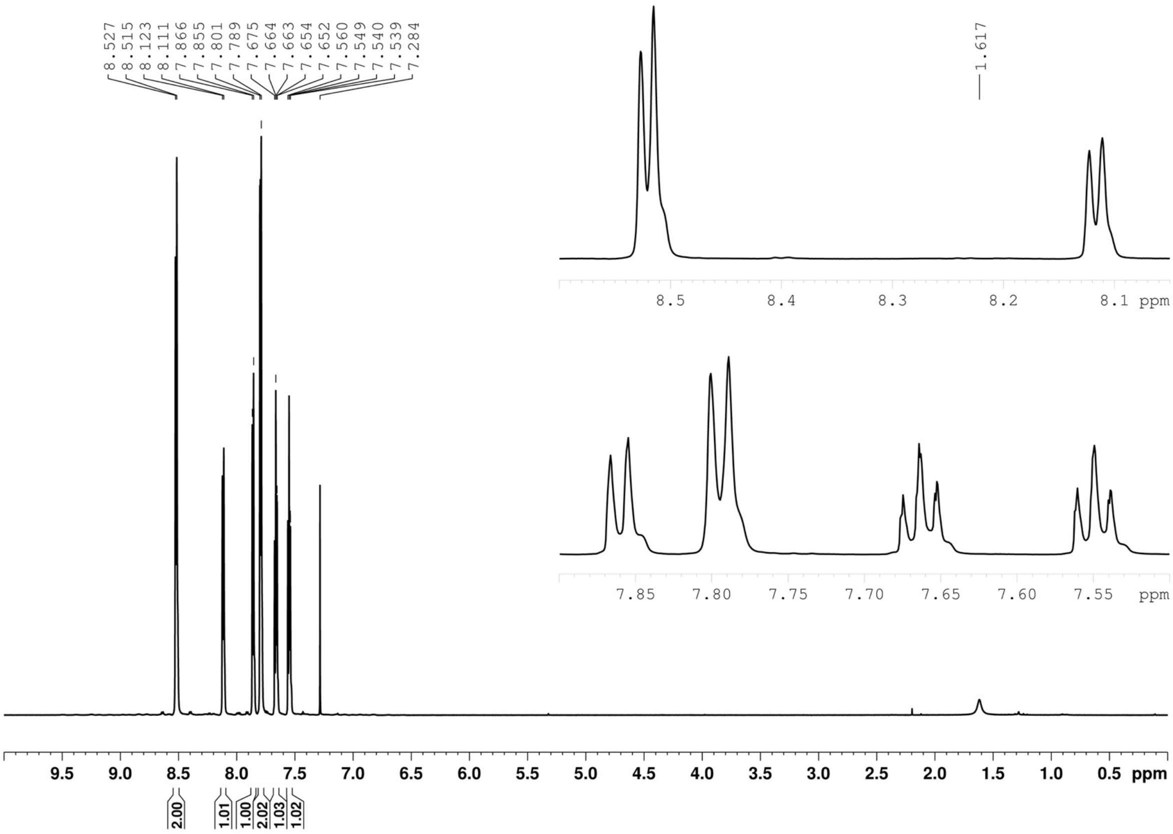

Figure S33: The [1]NMR spectrum of compound **6**.

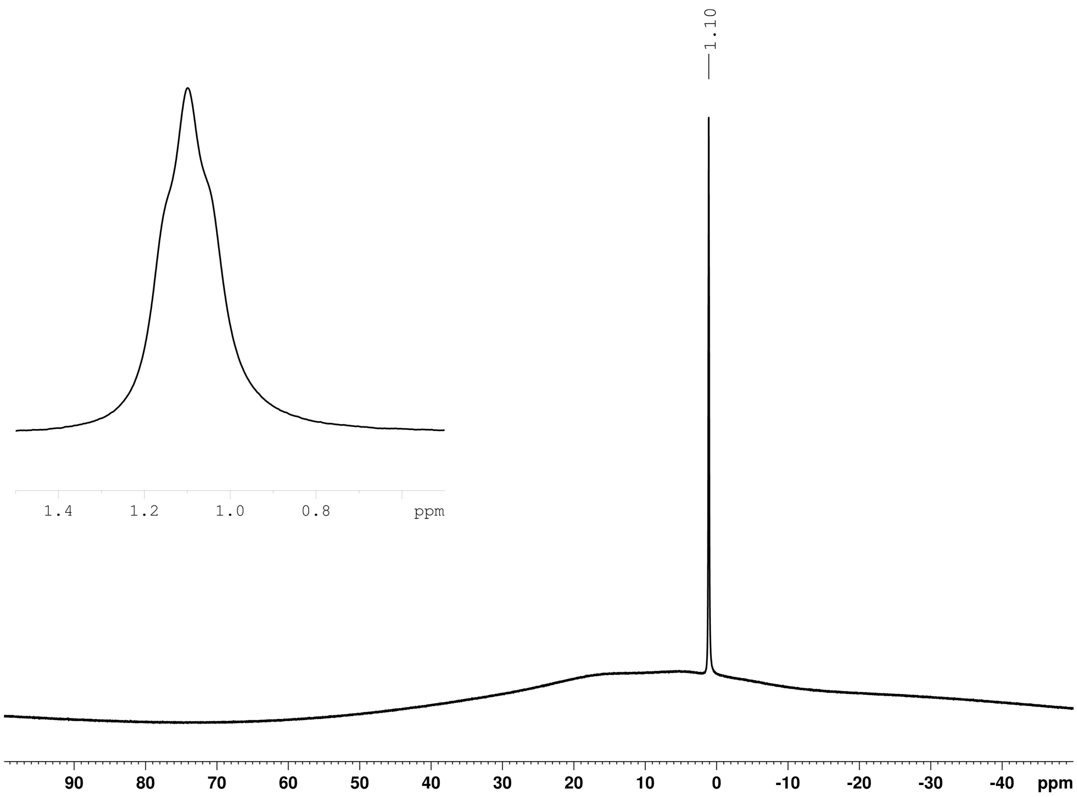

Figure S34: The ^{11}B NMR spectrum of compound **6**.

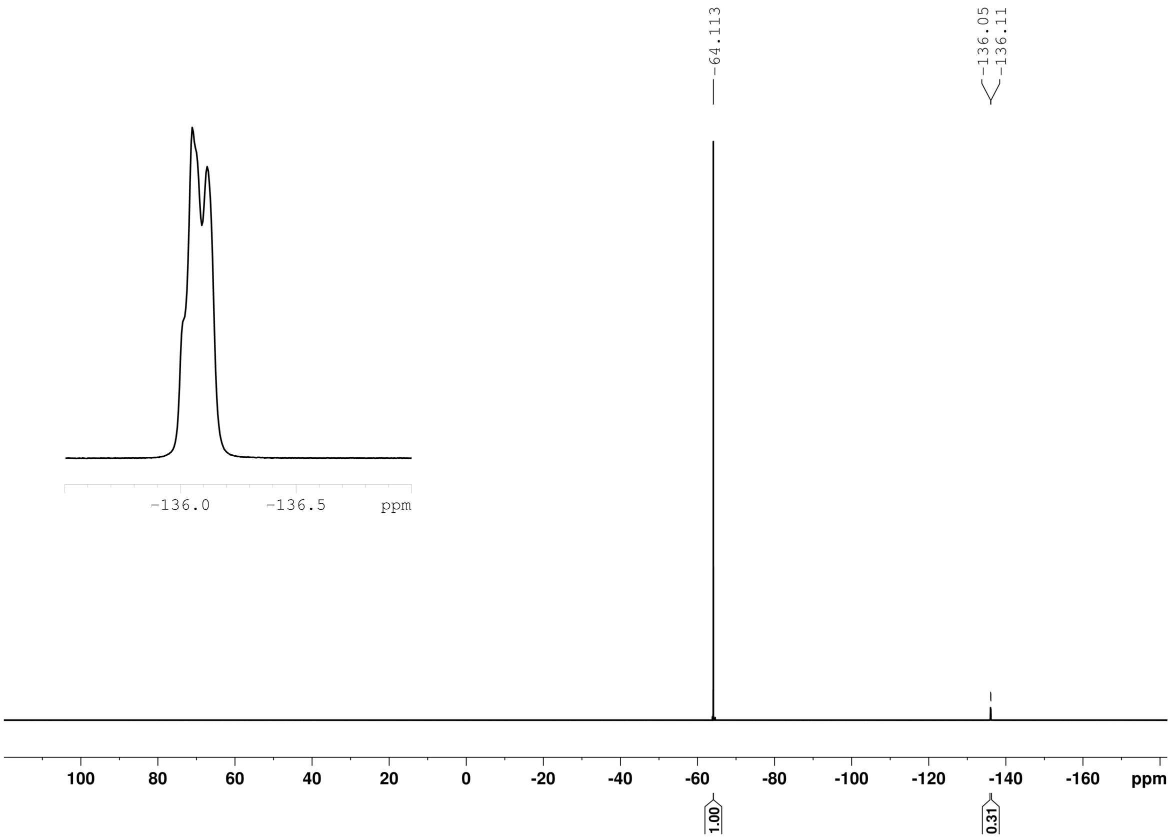

Figure S35: The ^{19}F NMR spectrum of compound **6**.

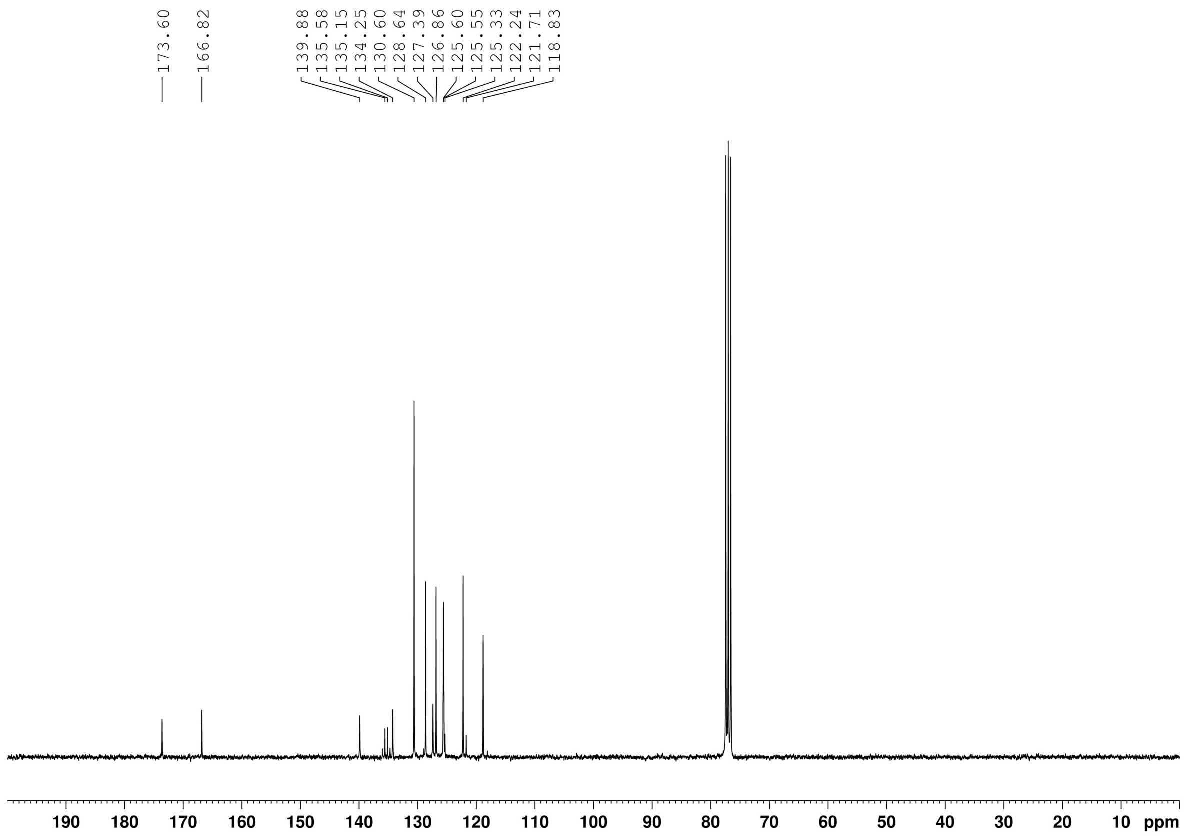

Figure S36: The ^{13}C NMR spectrum of compound **6**.

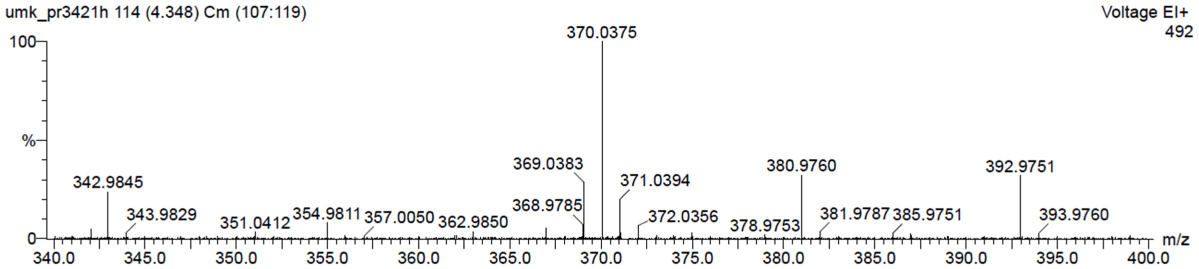

Figure S37: The MS spectrum of compound **6**.

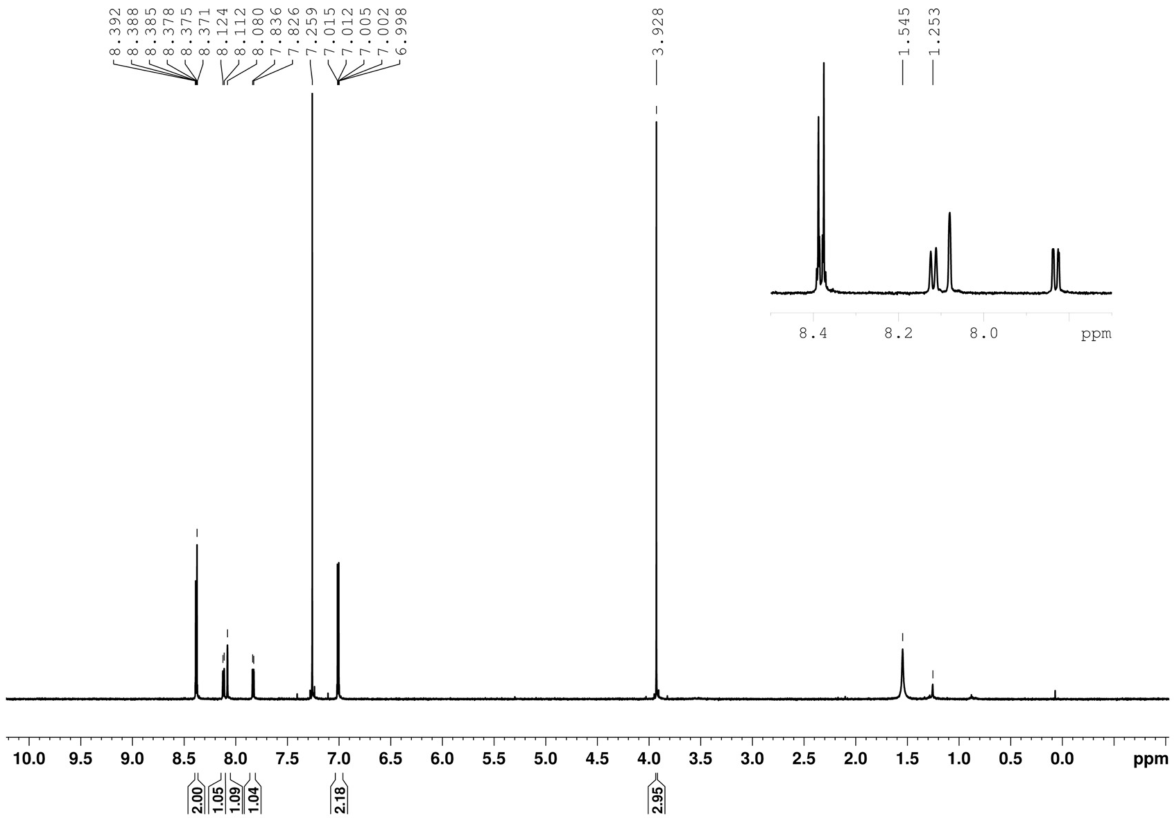

Figure S38: The ^{1}NMR spectrum of compound **7**.

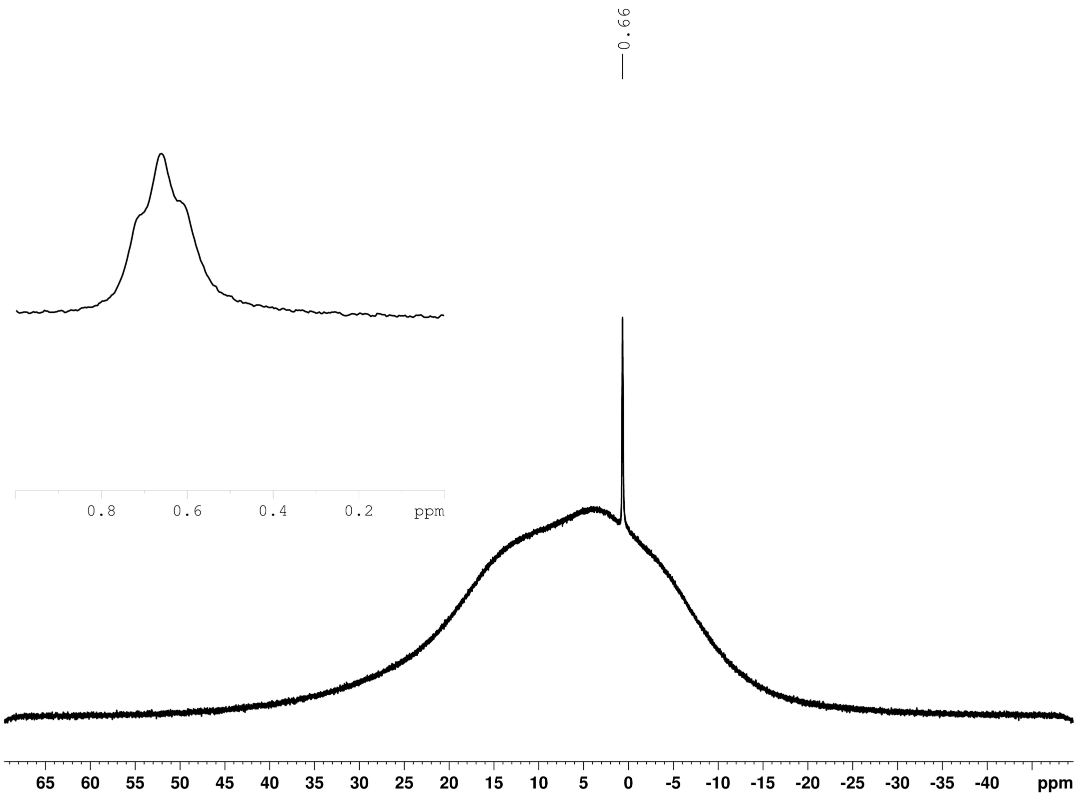

Figure S39: The ^{11}B NMR spectrum of compound **7**.

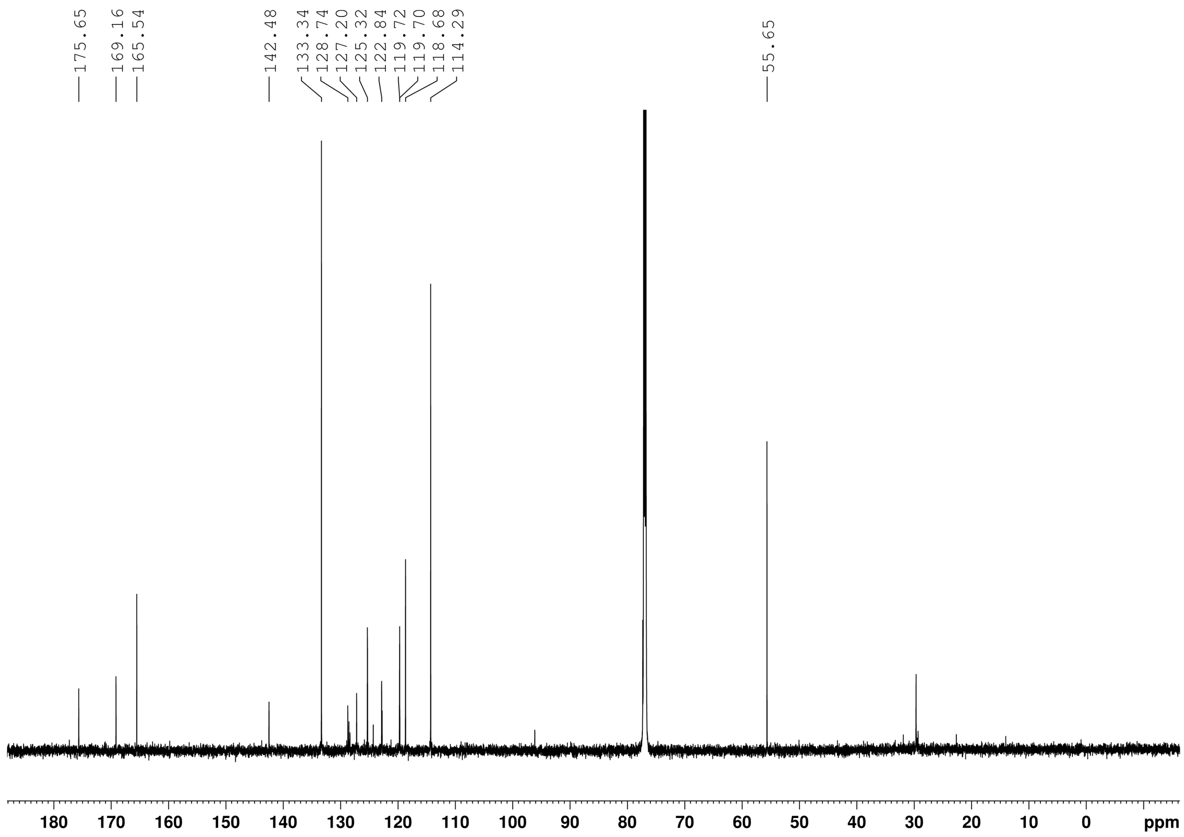

Figure S40: The ^{13}C NMR spectrum of compound **7**.

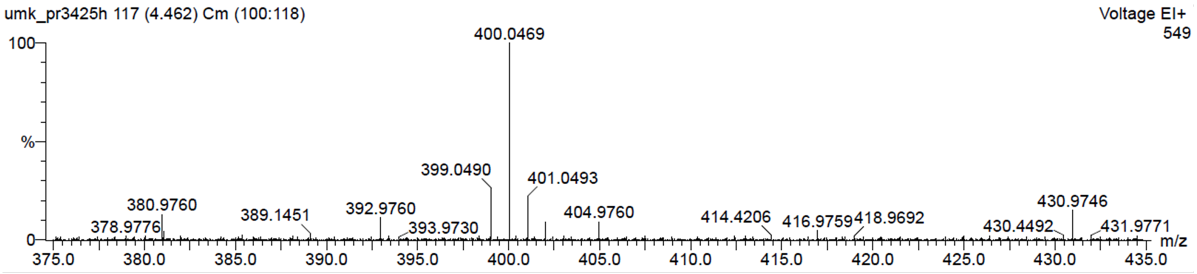

Figure S41: The MS spectrum of compound **7**.

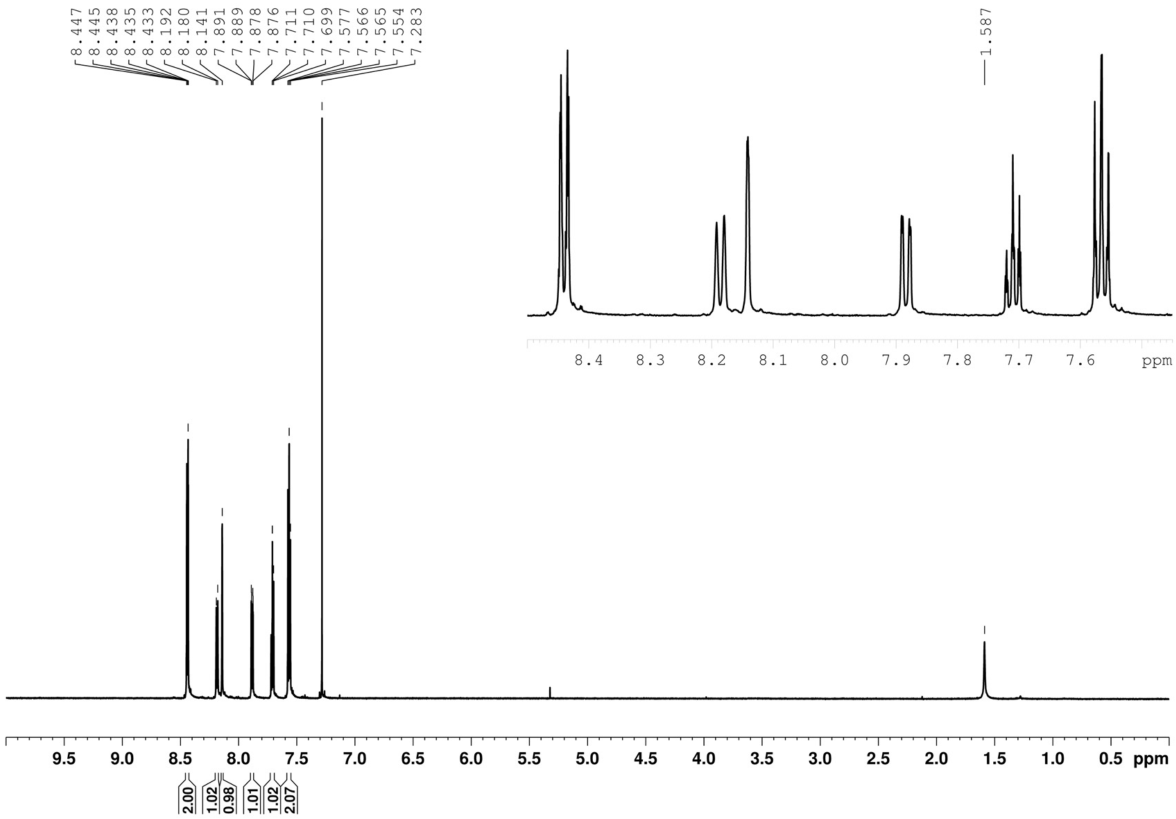

Figure S42: The ^{1}NMR spectrum of compound **8**.

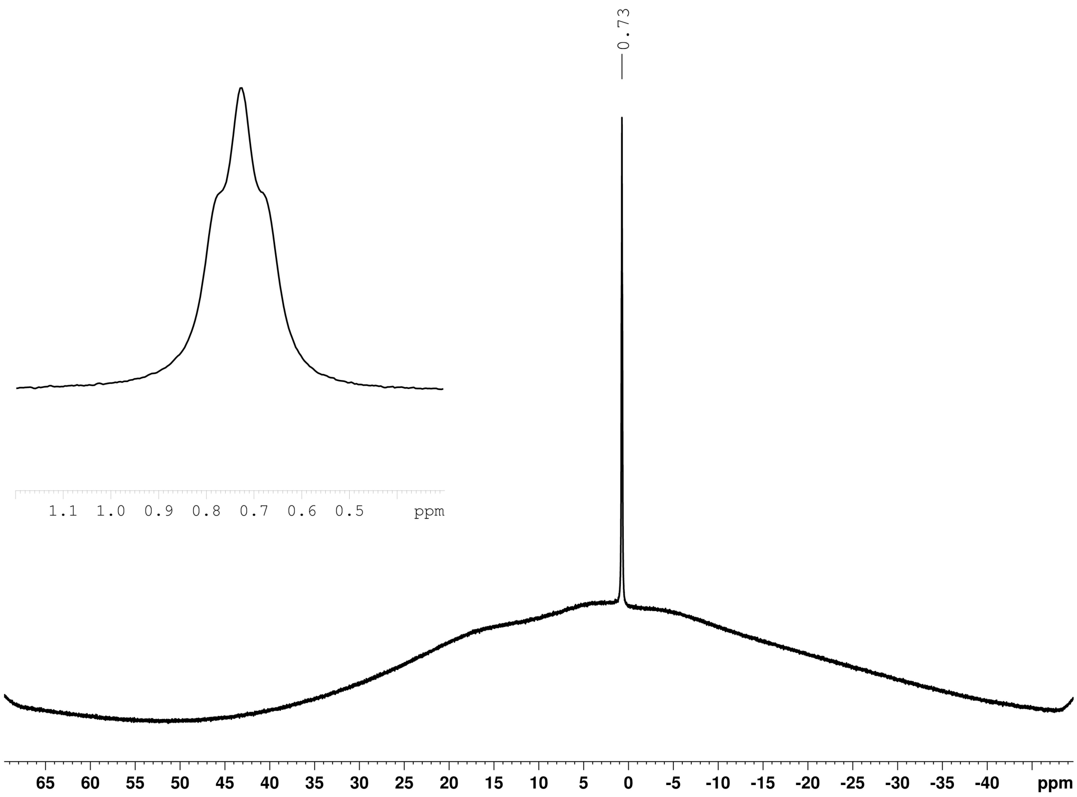

Figure S43: The ^{11}B NMR spectrum of compound **8**.

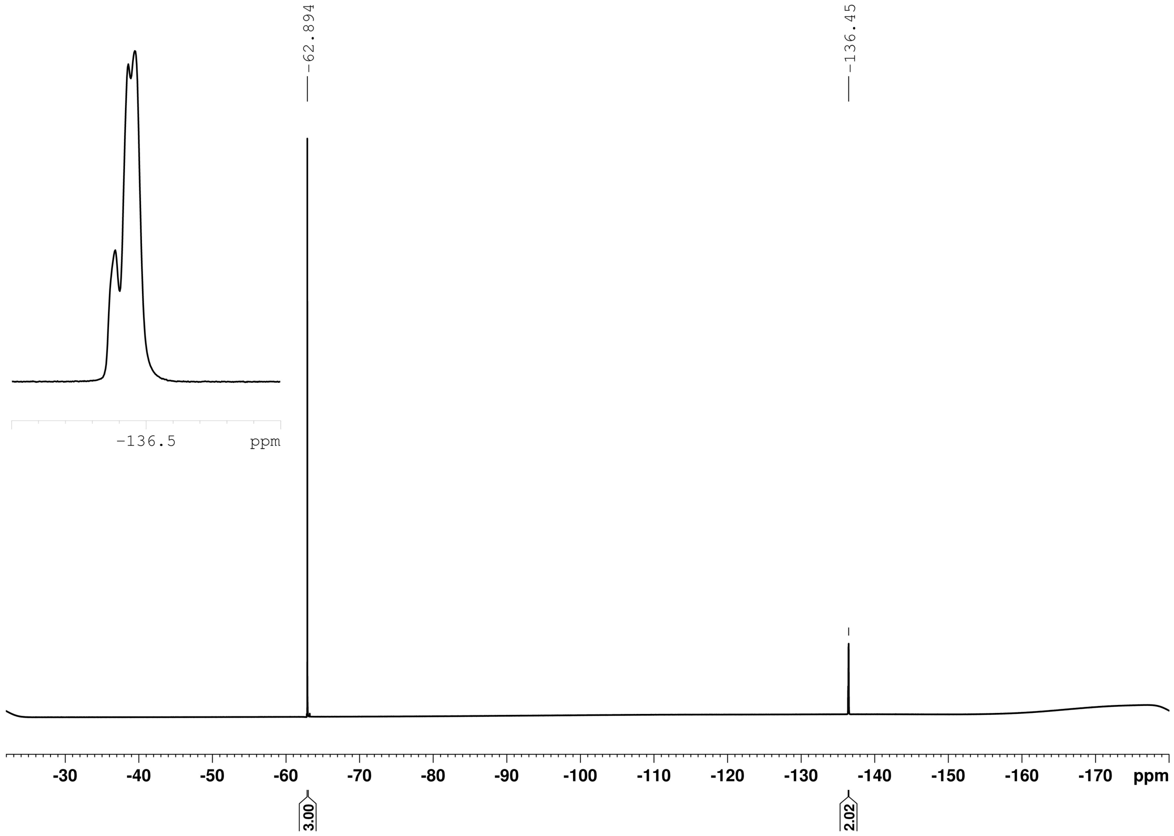

Figure S44: The ^{19}F NMR spectrum of compound **8**.

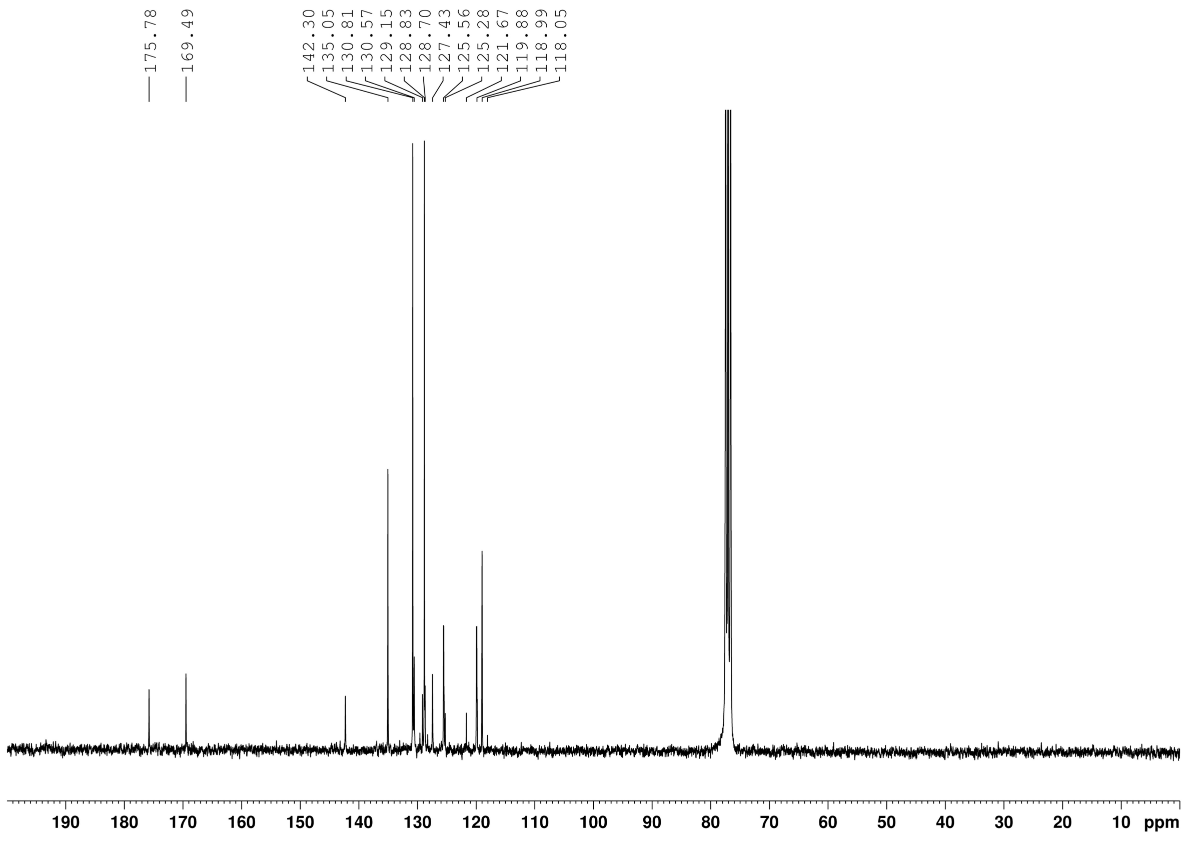

Figure S45: The ^{13}C NMR spectrum of compound **8**.

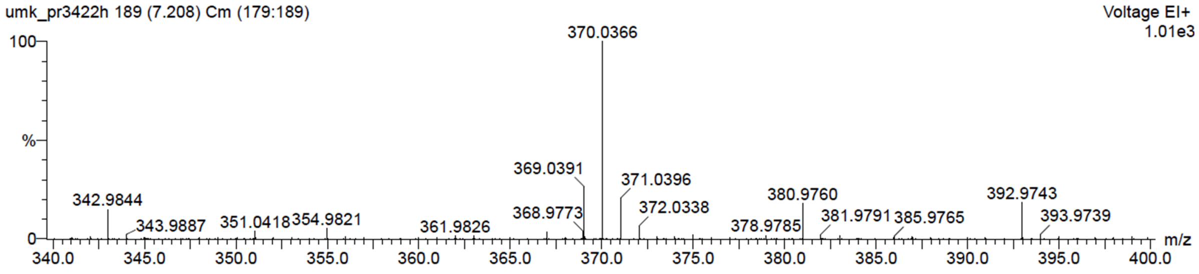

Figure S46: The MS spectrum of compound **8**.

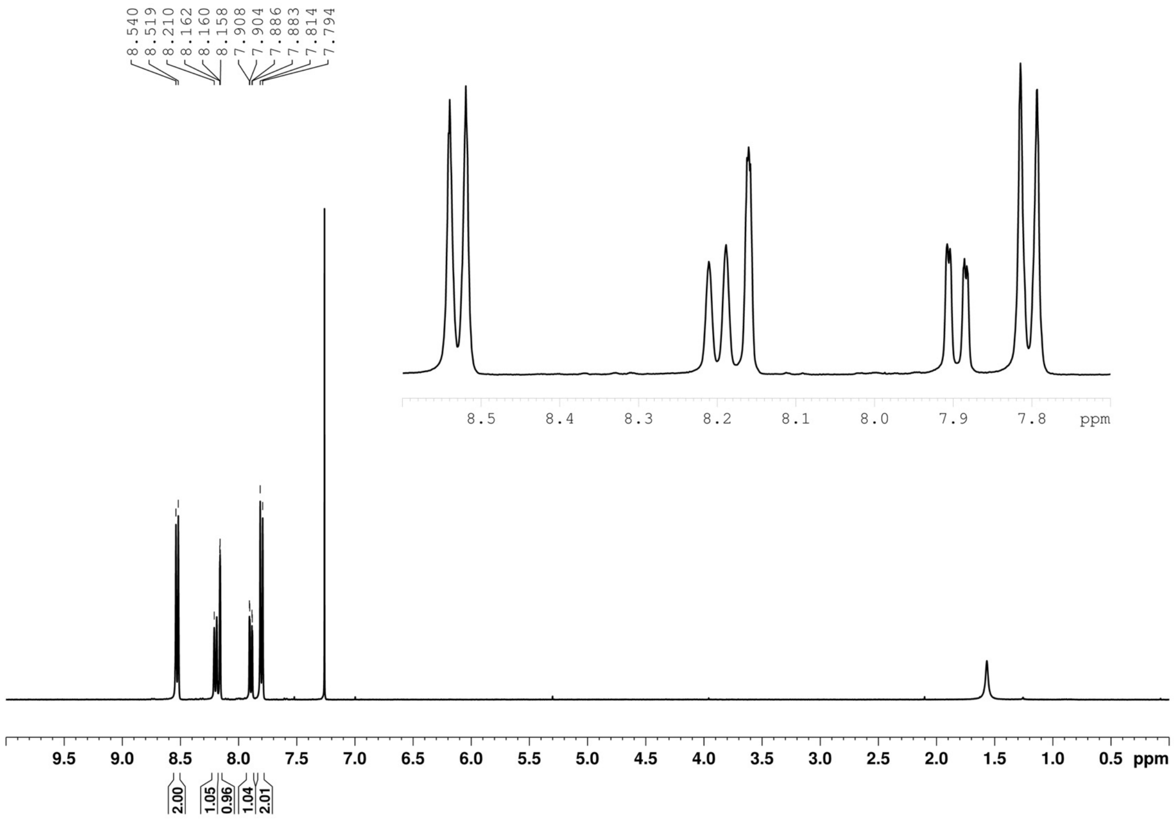

Figure S47: The ^{1}NMR spectrum of compound **9**.

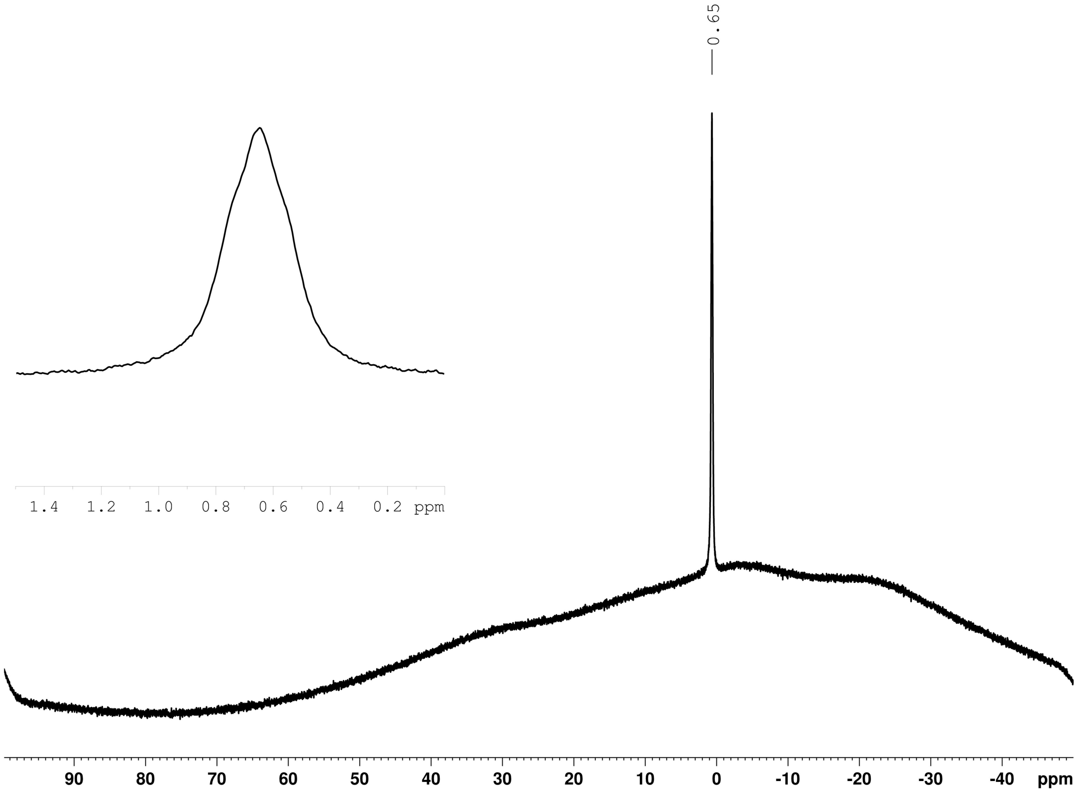

Figure S48: The ^{11}B NMR spectrum of compound **9**.

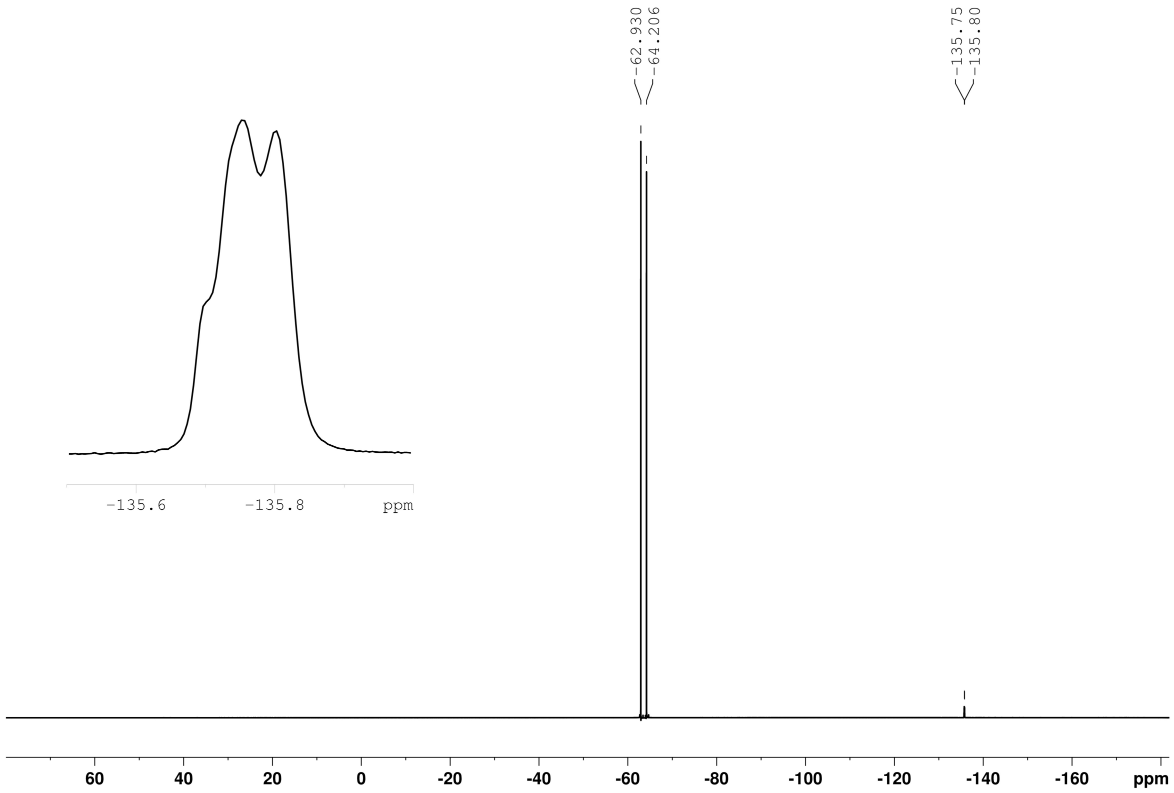

Figure S49: The ^{19}F NMR spectrum of compound **9**.

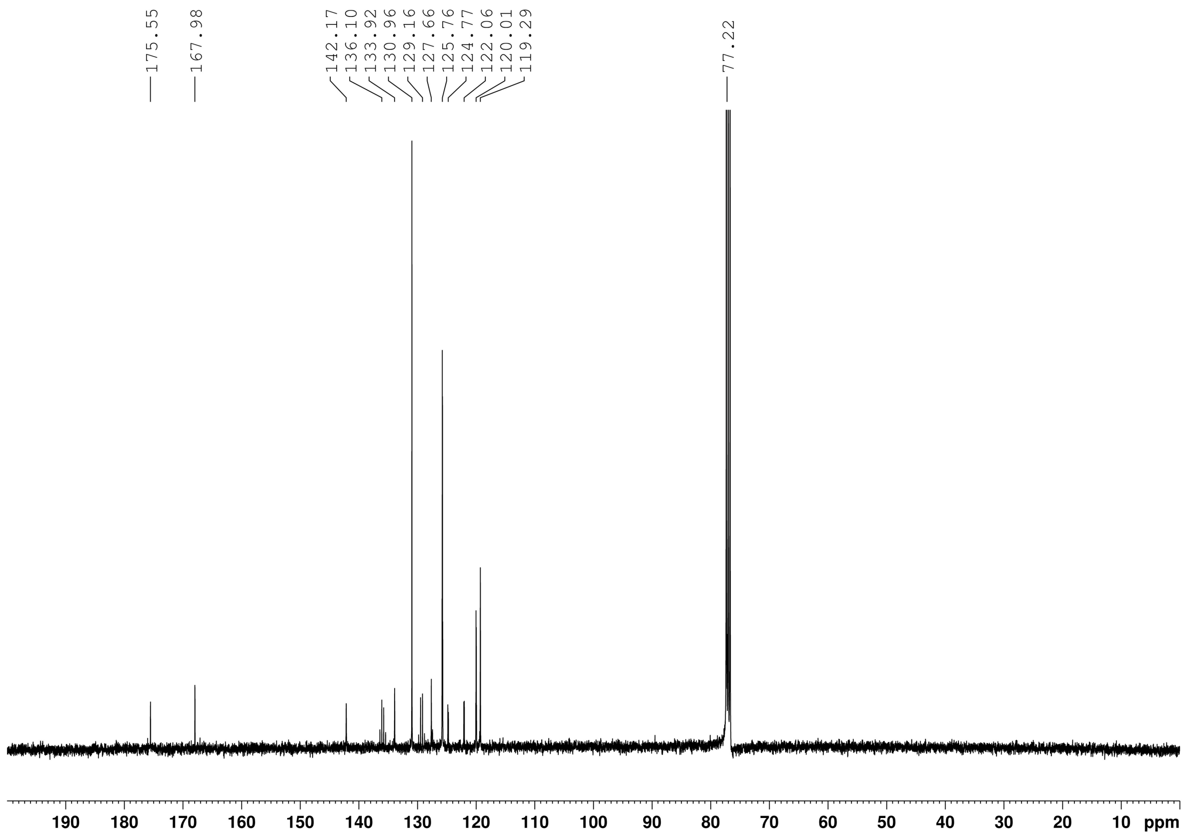

Figure S50: The ^{13}C NMR spectrum of compound **9**.

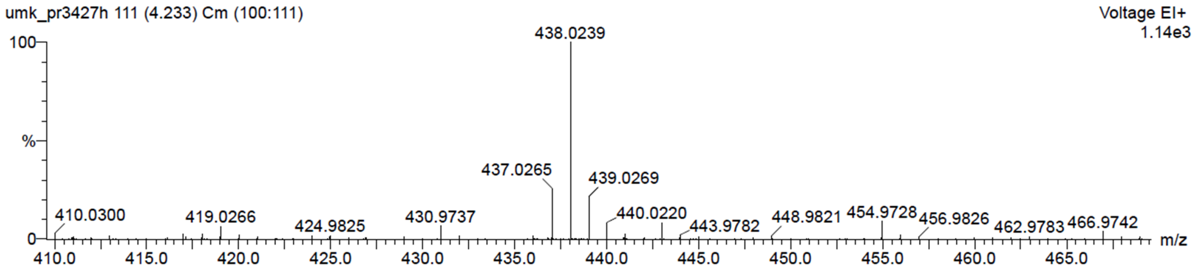

Figure S51: The MS spectrum of compound **9**.

Additional theoretical data

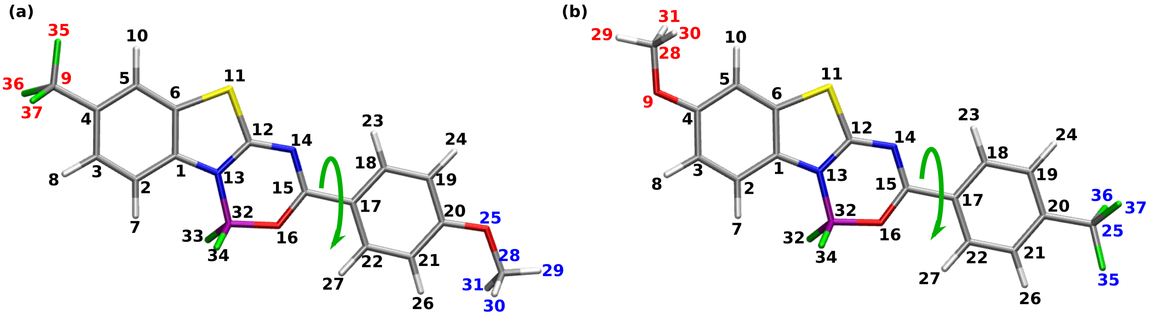

Figure S52: Atom numbering for two sample molecules, the green arrow indicating the investigated rotation between the benzothiazole core and the phenyl ring.

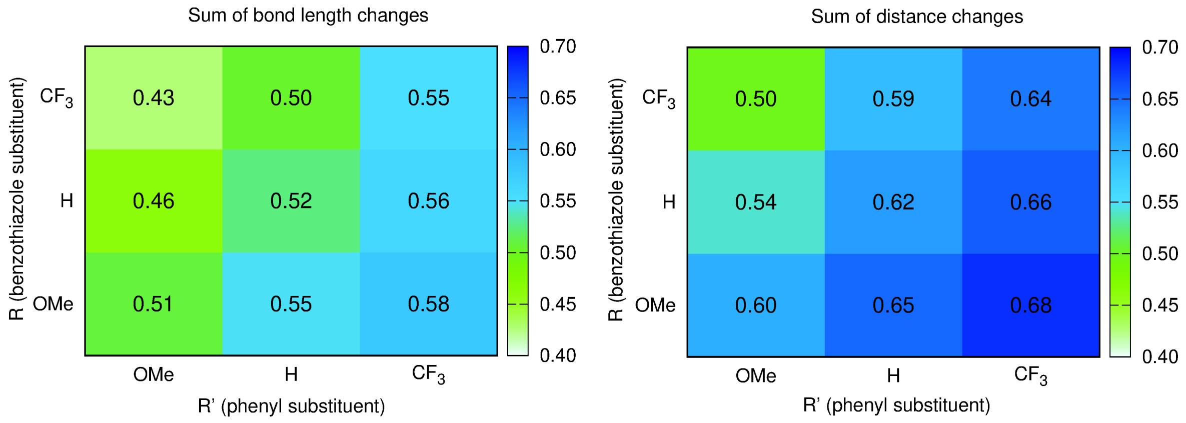

Figure S53: Modification of the bond length in the molecular scaffold (left panel) and interatomic distances (including selected non-bonding interactions in benzothiazole ring - right panel) in Å upon excitation, calculated with the PCM($CHCl_3$)-(TD-)MN15/6-311++G(d,p) approach (summation of the absolute values of S_1-S_0 distance differences, no B-F, C-H and in-substituent bonds included).

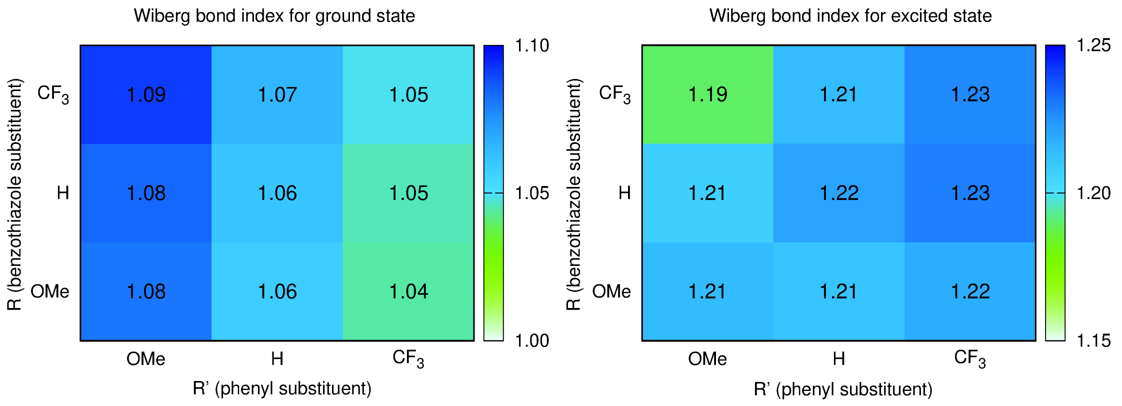

Figure S54: Wiberg bond indexes for the ground and excited states calculated within the PCM($CHCl_3$)-(TD-)MN15/6-311++G(d,p) approach (notice the shift of the scale between the two states).

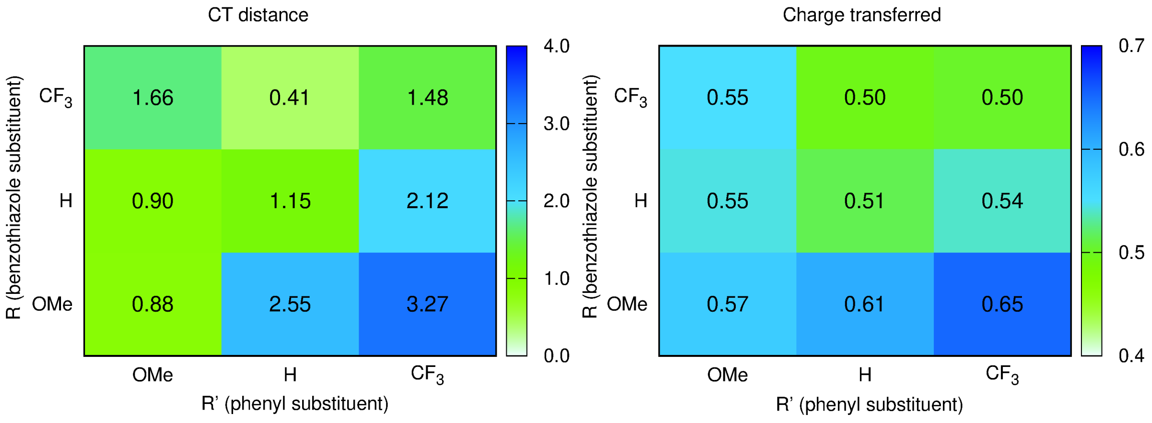

Figure S55: Charge transfer indexes according to Le Bahers' metric[S1] upon vertical excitation, calculated at the PCM($CHCl_3$)-(TD-)MN15/6-311++G(d,p) level: distance (Å) on the left panel and the amount of charge transferred (a.u.) on the right panel.

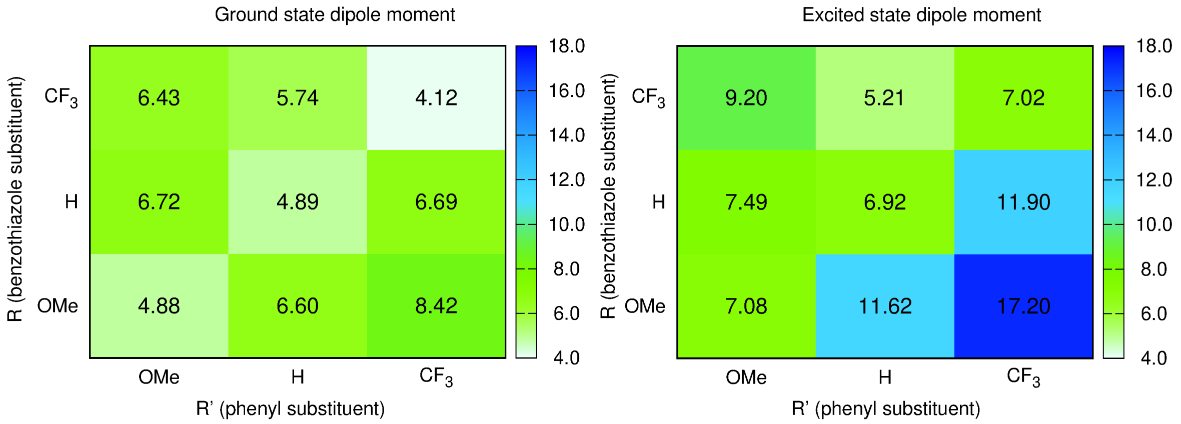

Figure S56: Dipole moment for the fully optimized ground and excited state structures calculated at the PCM($CHCl_3$)-(TD-)MN15/6-311++G(d,p) level.

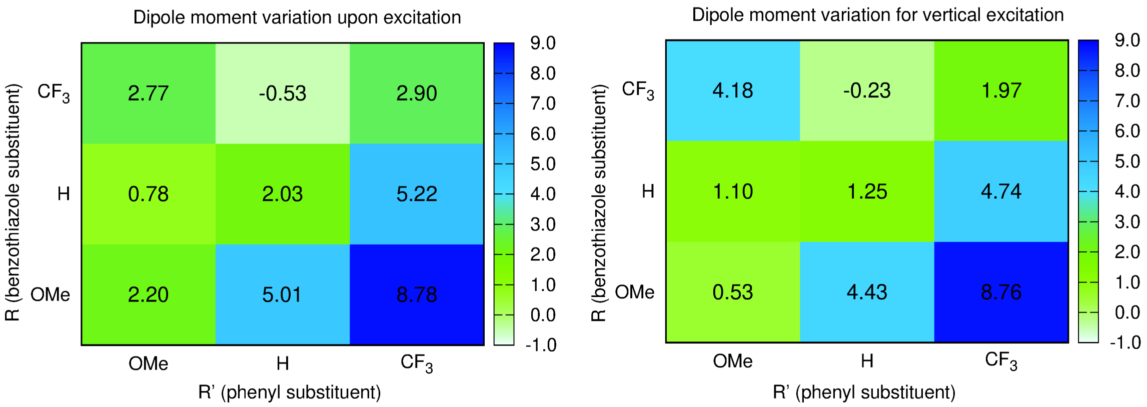

Figure S57: Dipole moment modification upon excitation, calculatedPCM($CHCl_3$)-TD-MN15/6-311++G(d,p) level considering (left panel) or not (right panel) excited-state geometrical relaxation.

Table S3: Barycenters (red for R_+ and blue for R_-) calculated at the PCM($CHCl_3$)-TD-MN15/6-311++G(d,p) level using Le Bahers' metric,[S1] on the ground state optimized geometry.

Compound (R/R')	Barycenters
**1** (OMe/OMe)	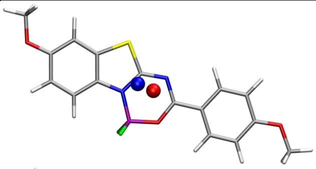
**2** (OMe/H)	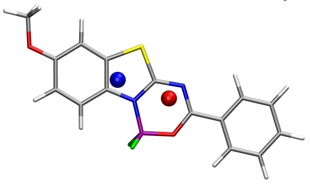
**3** (OMe/CF_3)	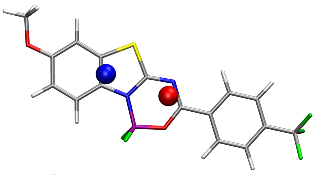
**4** (H/OMe)	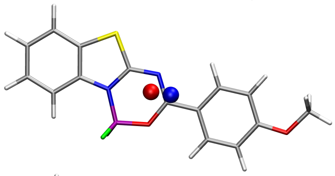
**5** (H/H)	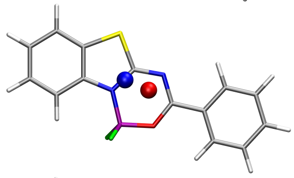
**6** (H/CF_3)	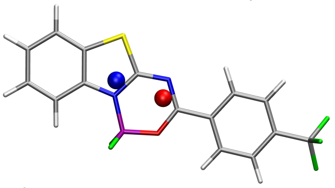
**7** (CF_3/OMe)	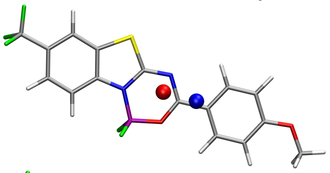
**8** (CF_3/H)	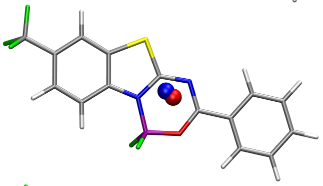
**9**(CF_3/CF_3)	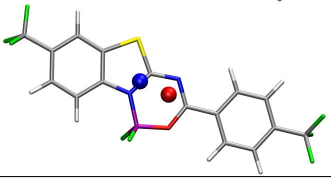

Table S4: Wiberg bond index for C15-C17 bond in ground state (S_0) and first singlet excited state (S_1), estimated at the PCM($CHCl_3$)-(TD-)MN15/6-311++G(d,p) level. For the excited state two approaches are reported: one for the relaxed first excited state density at the ground state geometry (FC regime) and the other at the optimized geometry of the first excited state).

Compound (R/R')	Ground state	Excited state	
	S_0 geometry	S_0 geometry	S_1 geometry
**1** (OMe/OMe)	1.0812	1.1428	1.2144
**2** (OMe/H)	1.0582	1.1333	1.2115
**3** (OMe/CF_3)	1.0444	1.1319	1.2183
**4** (H/OMe)	1.0839	1.1381	1.2073
**5** (H/H)	1.0610	1.1408	1.2209
**6** (H/CF_3)	1.0455	1.1396	1.2292
**7** (CF_3/OMe)	1.0911	1.1319	1.1898
**8** (CF_3/H)	1.0656	1.1391	1.2140
**9** (CF_3/CF_3)	1.0489	1.1391	1.2269

Table S5: Dipole moment in ground state (S_0) and lowest singlet excited state (S_1) determined at the PCM($CHCl_3$)-(TD-)MN15/6-311++G(d,p) level of theory. For comparison the excited state dipole moment for the vertical excitation (S_0 geometry) μ_{ES}^{vert}, $\Delta\mu$ and $\Delta\mu^{vert}$ are given as well. All data in D.

Compound (R/R')	μ_{S_0}	μ_{S_1}	$\mu_{S_1}^{vert}$	$\Delta\mu$	$\Delta\mu^{vert}$
**1** (OMe/OMe)	4.881	7.083	5.411	2.202	0.530
**2** (OMe/H)	6.605	11.632	11.036	5.027	4.432
**3** (OMe/CF_3)	8.376	17.202	17.181	8.826	8.806
**4** (H/OMe)	6.729	7.507	7.816	0.778	1.088
**5** (H/H)	4.887	6.922	6.147	2.035	1.259
**6** (H/CF_3)	6.644	11.912	11.427	5.268	4.783
**7** (CF_3/OMe)	6.427	9.201	10.609	2.773	4.181
**8** (CF_3/H)	5.736	5.213	5.501	-0.523	-0.235
**9** (CF_3/CF_3)	4.056	7.029	6.091	2.973	2.036

In the publication by Humeniuk *et al.*[S2] the various ways of obtaining k_{ic} are discussed and its dependency on the broadening function is especially stressed out The authors concluded that the use of Lorentzian profiles can greatly impact the k_{ic} rate but an 'accepted' broadening value of *ca.* 10 cm^{-1} remains admitted among the literature.[S2,S3] In Tables S6 and S7, we compare the rates obtained from the internal Vertical Hessian and Cartesian Adiabatic Hessian vibronic models and observe significant modifications of those k_{ic} rates for some compounds whereas comparable k_{r} rates are found. We also show in Figs. S58 and S59 the spectra comparing the two vibronic models. Likely here, the k_{ic} values are too strongly dependent on the calculation model, nevertheless no model is apparently superior to the other in what concerns reproducing experimental trends. Therefore, we pursued with the AH model in the body of the text since: i) almost no improvement of the quantum yield is observed with the VH model, ii) the accessibility of the MECP governs the predicted quantum yield, and iii) the final correlation between the experimental and theoretical ϕ_{f} is not significantly modified when using one or the other vibronic model. In addition in Fig. S61 the interested reader may found a comparison of the vibronic models is Cartesian coordinates only.

Table S6: Comparison of radiative and internal conversion rates obtained from vibronic calculations only, these calculations being made with the cAH model. All values are obtained from the Time-Dependent Formalism.

Compound (R/R')	ϕ_f^{expt}	k_r^{expt} (10^7 [s^{-1}])	model	k_{ic} (10^7 [s^{-1}])	k_r (10^7 [s^{-1}])	ϕ_f
**1** (OMe/OMe)	13.1	28.50	cAH	8.35	30.85	78.7
**2** (OMe/H)	0.9	7.14	cAH	78.70	21.75	21.7
**3** (OMe/CF_3)	0.4	4.21	cAH	21.58	20.17	48.3
**4** (H/OMe)	75.6	44.20	cAH	3.57	47.64	93.0
**5** (H/H)	6.9	33.70	cAH	5.65	32.13	85.0
**6** (H/CF_3)	0.4	3.01	cAH	14.99	26.98	64.3
**7** (CF_3/OMe)	98.0	59.30	cAH	2.61	56.14	95.6
**8** (CF_3/H)	28.0	27.60	cAH	2.78	39.19	93.4
**9** (CF_3/CF_3)	4.7	20.60	cAH	21.40	27.77	56.5
**RMS**					0.87	0.44

Table S7: Comparison of radiative and internal conversion rates obtained from vibronic calculations only using the iVH model. All values are obtained from the Time-Dependent Formalism.

Compound (R/R')	ϕ_f^{expt}	k_r^{expt} (10^7 [s^{-1}])	model	k_{ic} (10^7 [s^{-1}])	k_r (10^7 [s^{-1}])	ϕ_f
**1** (OMe/OMe)	13.1	28.50	iVH	4.13	36.85	89.9
**2** (OMe/H)	0.9	7.14	iVH	9.40	26.75	74.0
**3** (OMe/CF_3)	0.4	4.21	iVH	4.19	26.25	86.2
**4** (H/OMe)	75.6	44.20	iVH	2.59	55.32	95.5
**5** (H/H)	6.9	33.70	iVH	2.16	38.31	94.7
**6** (H/CF_3)	0.4	3.01	iVH	2.81	34.06	92.4
**7** (CF_3/OMe)	98.0	59.30	iVH	1.78	57.47	97.0
**8** (CF_3/H)	28.0	27.60	iVH	1.87	45.26	96.0
**9** (CF_3/CF_3)	4.7	20.60	iVH	2.65	35.63	93.1
**RMS**					0.84	0.27

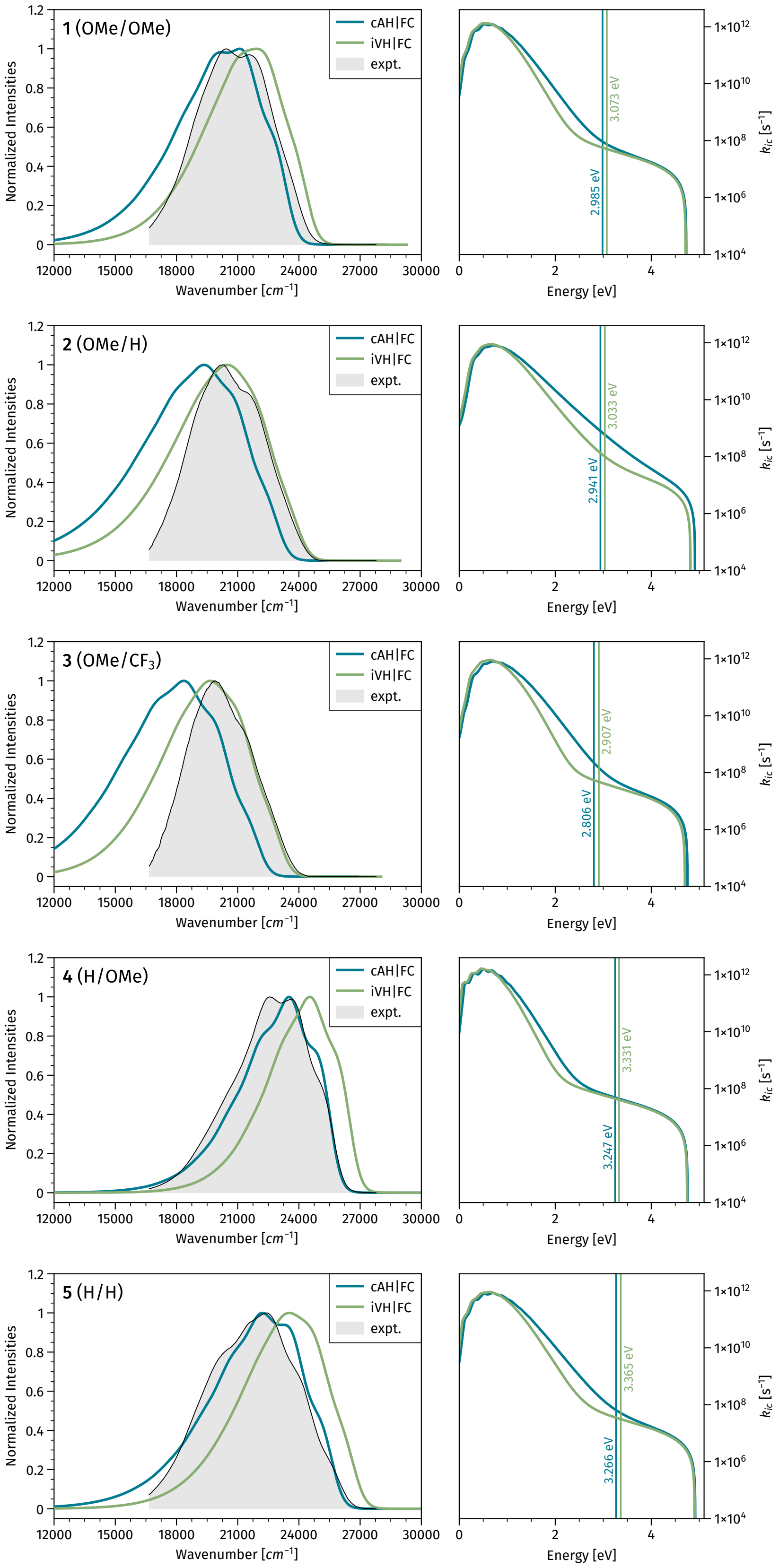

Figure S58: Comparisons of two vibronic schemes approaches, namely Vertical Hessian using internal coordinate systems (iVH) and Adiabatic Hessian with Cartesian coordinates (cAH). (left) Lineshapes of theoretical and experimental emission spectra, normalized. (right) $k_{\rm ic}$ rate *versus* energy plots. The emission spectra are convoluted with a Gaussian broadening of 450 cm^{-1}, internal conversion plot were convoluted with a Lorentzian 10 cm^{-1} broadening. All spectra are obtained from the Time-Dependent Formalism.

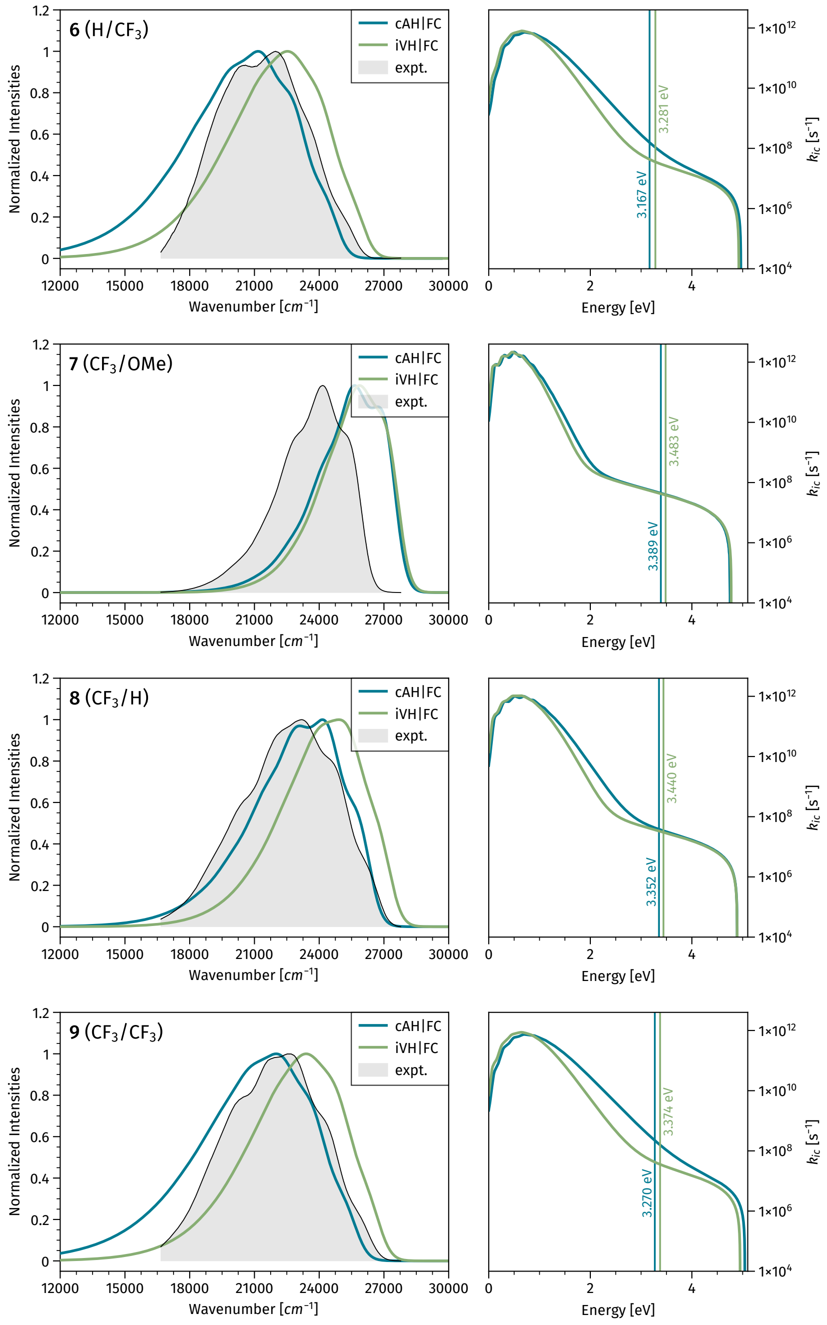

Figure S59: Comparisons of two vibronic schemes approaches, namely Vertical Hessian using internal coordinate systems (iVH) and Adiabatic Hessian with Cartesian coordinates (cAH). (left) Lineshapes of theoretical and experimental emission spectra, normalized. (right) k_{ic} rate *versus* energy plots. The emission spectra are convoluted with a Gaussian broadening of 450 cm^{-1}, internal conversion plot were convoluted with a Lorentzian 10 cm^{-1} broadening. All spectra are obtained from the Time-Dependent Formalism.

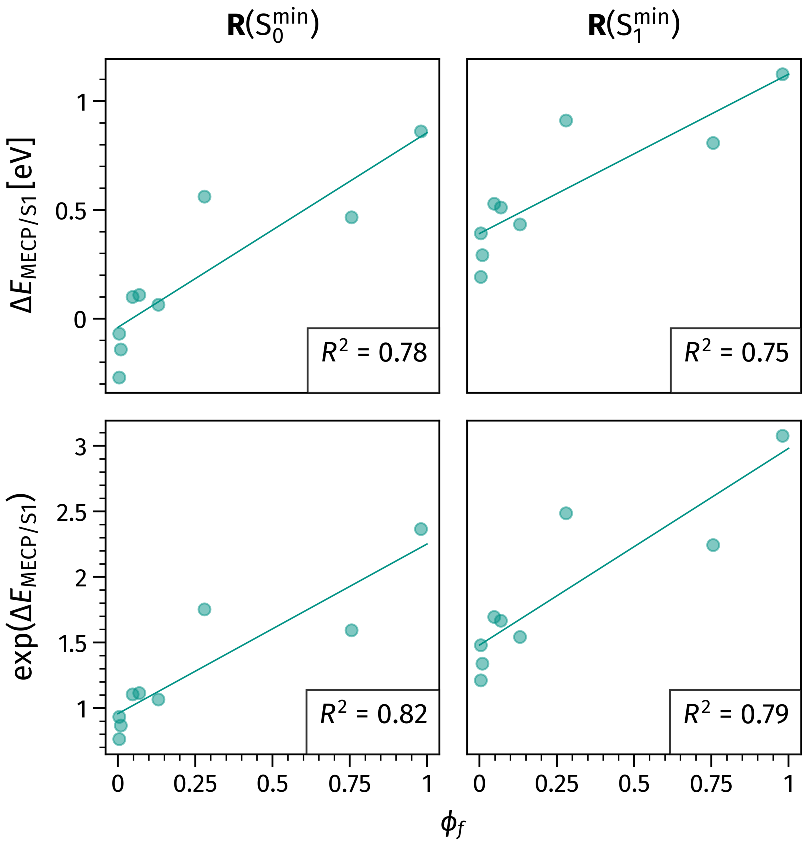

Figure S60: Comparison of linear correlation between the energy difference between the MECP and S_1 at the ground-state minimum (FC, left) and the MECP and the S_1 at the excited-state minimum (right).

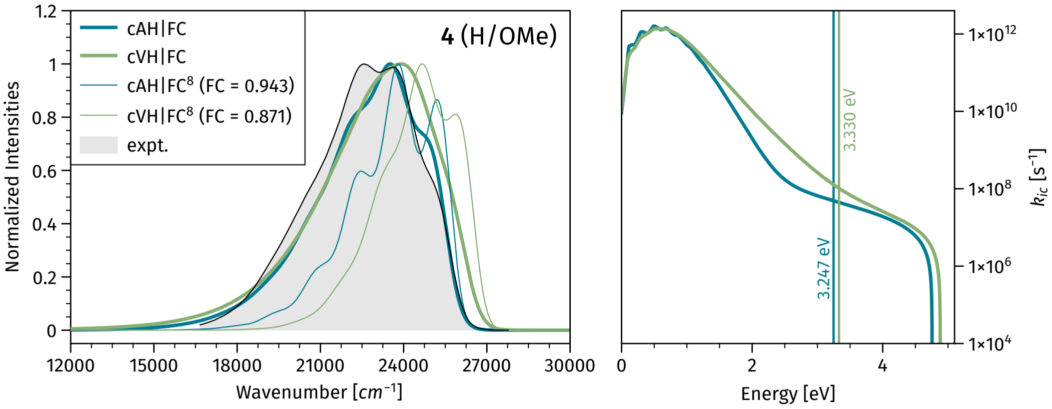

Figure S61: Comparisons of various vibronic schemes approaches for dye **4** in Cartesian coordinates. Lineshapes of theoretical and experimental emission spectra, normalized. (left) k_{ic} rate *versus* energy plots. The emission spectra are convoluted with a Gaussian broadening of $450\,cm^{-1}$, internal conversion plot were convoluted with a Lorentzian $10\,cm^{-1}$ broadening.

Below are the Cartesian coordinates of the S_0 and S_1 minima (no imaginary frequencies) as computed at the PCM($CHCl_3$)-TD-MN15/6-311+G(d,p) level. We report coordinates (in Å) and total (ground-state) energies (in Hartree).

Dye 1

$\mathbf{S}_0$, E= -1574.60522470

C	3.0819510	-0.8511010	-0.0000030
C	2.5656930	0.4444310	-0.0000050
C	3.4240840	1.5473140	-0.0000120
C	4.7841430	1.3162560	-0.0000170
C	5.3041270	0.0062770	-0.0000150
C	4.4558710	-1.0973200	-0.0000090
C	0.6177980	-0.7351230	0.0000120
H	3.0221660	2.5528600	-0.0000160
H	5.4880290	2.1392170	-0.0000230
H	4.8326700	-2.1106520	-0.0000070
S	1.7835380	-2.0134500	0.0000110
N	-0.6947390	-1.0036820	0.0000230
C	-1.5113570	0.0313090	-0.0000040
C	-2.9568840	-0.1868310	-0.0000030
C	-3.4806480	-1.4907910	0.0000150
C	-3.8341220	0.9006030	-0.0000190
C	-4.8444400	-1.6935320	0.0000170
H	-2.7994080	-2.3324700	0.0000280
C	-5.2093800	0.7070560	-0.0000180
H	-3.4321980	1.9059600	-0.0000320
C	-5.7198470	-0.5959370	-0.0000010
H	-5.2680730	-2.6903910	0.0000300
H	-5.8683010	1.5648530	-0.0000280
O	-1.1185440	1.2746000	-0.0000370
B	0.2811330	1.7483210	0.0000210
F	0.5288540	2.4949350	1.1427570
F	0.5289160	2.4950170	-1.1426470
N	1.1721470	0.4711110	0.0000020
O	-7.0346710	-0.8884240	0.0000030
C	-7.9682760	0.1801390	-0.0000430
H	-7.8533860	0.7999800	-0.8934490
H	-8.9549840	-0.2756860	-0.0000600
H	-7.8534340	0.8000160	0.8933440
O	6.6544020	-0.0853430	-0.0000220

C	7.2360180	-1.3765230	-0.0000180
H	8.3131200	-1.2301830	-0.0000190
H	6.9459280	-1.9377470	0.8934830
H	6.9459250	-1.9377540	-0.8935120

$\mathbf{S}_1$, E= -1574.59378927

C	3.0690190	-0.8852720	-0.0000120
C	2.5329360	0.4364550	-0.0000110
C	3.4042780	1.5504200	0.0000020
C	4.7562020	1.3236240	0.0000130
C	5.2811270	0.0029330	0.0000120
C	4.4321860	-1.1137180	-0.0000010
C	0.5903510	-0.7748130	-0.0000240
H	2.9916550	2.5508840	0.0000030
H	5.4642370	2.1428530	0.0000240
H	4.8226180	-2.1218800	-0.0000020
S	1.7939250	-2.0650370	-0.0000260
N	-0.6813400	-0.9977450	-0.0000190
C	-1.5333600	0.0702680	-0.0000220
C	-2.9350990	-0.1593370	-0.0000100
C	-3.4680390	-1.4807320	0.0000040
C	-3.8465820	0.9274640	-0.0000130
C	-4.8250100	-1.6888870	0.0000150
H	-2.7831120	-2.3189870	0.0000060
C	-5.2124470	0.7152710	-0.0000020
H	-3.4547730	1.9366160	-0.0000240
C	-5.7159670	-0.5969700	0.0000120
H	-5.2416540	-2.6892490	0.0000260
H	-5.8827430	1.5650690	-0.0000050
O	-1.1041270	1.3290470	-0.0000440
B	0.2881030	1.7582800	-0.0000030
F	0.5747170	2.5083970	1.1420710
F	0.5747560	2.5084630	-1.1420230
N	1.1833940	0.4769910	-0.0000210
O	-7.0274780	-0.9021650	0.0000230
C	-7.9745280	0.1538930	0.0000140
H	-7.8663240	0.7753680	-0.8933400
H	-8.9554690	-0.3142670	0.0000210
H	-7.8663200	0.7753860	0.8933560
O	6.6143370	-0.0806210	0.0000260
C	7.2295560	-1.3630480	0.0000330
H	8.3010470	-1.1856930	0.0000480
H	6.9491220	-1.9243870	0.8947690

H	6.9491470	-1.9243860	-0.8947110

Dye 2

$\mathbf{S}_0$, E= -1460.16531679

C	2.3765630	-0.8396250	-0.0000150
C	1.8420300	0.4486530	-0.0000200
C	2.6845360	1.5641190	-0.0000210
C	4.0472290	1.3520020	-0.0000250
C	4.5859150	0.0490310	-0.0000310
C	3.7538910	-1.0666490	-0.0000220
C	-0.0865340	-0.7590540	0.0000130
H	2.2686290	2.5639640	-0.0000220
H	4.7396750	2.1845910	-0.0000240
H	4.1450180	-2.0745510	-0.0000210
S	1.0952590	-2.0198130	0.0000160
N	-1.3981550	-1.0460510	0.0000330
C	-2.2252980	-0.0243180	-0.0000270
C	-3.6775040	-0.2641600	-0.0000280
C	-4.1705650	-1.5742380	0.0000360
C	-4.5631450	0.8189030	-0.0000910
C	-5.5412850	-1.7961520	0.0000390
H	-3.4712360	-2.4008870	0.0000840
C	-5.9339490	0.5904320	-0.0000870
H	-4.1699840	1.8275120	-0.0001400
C	-6.4231910	-0.7150060	-0.0000220
H	-5.9244310	-2.8095410	0.0000890
H	-6.6207130	1.4282770	-0.0001340
O	-1.8548310	1.2237270	-0.0001050
B	-0.4613200	1.7203640	0.0000460
F	-0.2257500	2.4681280	1.1430150
F	-0.2255930	2.4683580	-1.1427330
N	0.4484100	0.4546810	-0.0000070
O	5.9367140	-0.0226780	-0.0000330
C	6.5378000	-1.3051510	-0.0000100
H	7.6125100	-1.1424740	-0.0000000
H	6.2559920	-1.8703530	0.8935570
H	6.2560210	-1.8703650	-0.8935850
H	-7.4927550	-0.8908910	-0.0000190

$\mathbf{S}_1$, E= -1460.15160234

C	2.3654280	-0.8752190	-0.0000400
C	1.8088210	0.4405360	-0.0000310
C	2.6618710	1.5703600	0.0000050
C	4.0157950	1.3658570	0.0000350
C	4.5613650	0.0512370	0.0000300
C	3.7293600	-1.0816870	-0.0000110
C	-0.1146590	-0.8018530	-0.0000690
H	2.2319990	2.5634750	0.0000090
H	4.7118590	2.1950590	0.0000650
H	4.1374650	-2.0829130	-0.0000180
S	1.1080410	-2.0749710	-0.0000860
N	-1.3792420	-1.0451050	-0.0000580
C	-2.2517420	0.0125210	-0.0000540
C	-3.6515390	-0.2407020	-0.0000110
C	-4.1533970	-1.5699270	0.0000300
C	-4.5764400	0.8360690	-0.0000050
C	-5.5165950	-1.8000040	0.0000770
H	-3.4504390	-2.3932310	0.0000250
C	-5.9362670	0.5857690	0.0000400
H	-4.1987100	1.8506500	-0.0000360
C	-6.4192170	-0.7292710	0.0000820
H	-5.8881550	-2.8184420	0.0001090
H	-6.6341460	1.4153740	0.0000430
O	-1.8418560	1.2720350	-0.0001080
B	-0.4549120	1.7280780	-0.0000130
F	-0.1844180	2.4788760	1.1425440
F	-0.1843270	2.4790340	-1.1424410
N	0.4604730	0.4607090	-0.0000560
O	5.8912450	-0.0105640	0.0000700
C	6.5347110	-1.2810660	0.0000930
H	7.6015920	-1.0792550	0.0001390
H	6.2654810	-1.8462840	0.8952920
H	6.2655590	-1.8462780	-0.8951330
H	-7.4866080	-0.9169240	0.0001190

Dye 3

$\mathbf{S}_0$, E= -1797.04556212

C	-3.6454220	-0.8958910	-0.0009440
C	-3.1892690	0.4224230	0.0020840
C	-4.0969150	1.4858910	0.0093300
C	-5.4436750	1.1918460	0.0131520

C	-5.9036120	-0.1416520	0.0098280
C	-5.0067380	-1.2057850	0.0027760
C	-1.1943750	-0.6677320	-0.0085460
H	-3.7416420	2.5087870	0.0118930
H	-6.1852540	1.9808980	0.0188420
H	-5.3365860	-2.2354380	0.0001450
S	-2.2956690	-1.9962420	-0.0091140
N	0.1354410	-0.8735400	-0.0141560
C	0.8937560	0.1957160	-0.0147490
C	2.3623660	0.0408360	-0.0205570
C	2.9266900	-1.2383380	-0.0232330
C	3.1809120	1.1736620	-0.0261150
C	4.3062480	-1.3851610	-0.0291310
H	2.2769350	-2.1038690	-0.0220880
C	4.5617500	1.0281680	-0.0327780
H	2.7315040	2.1579650	-0.0269870
C	5.1146800	-0.2499670	-0.0360020
H	4.7532610	-2.3713510	-0.0356750
H	5.2045740	1.8995740	-0.0420450
O	0.4550840	1.4199000	-0.0103390
B	-0.9683690	1.8325600	-0.0010510
F	-1.2536980	2.5672540	-1.1393290
F	-1.2418500	2.5569560	1.1467110
N	-1.7988990	0.5117460	-0.0026030
O	-7.2470170	-0.2924690	0.0139830
C	-7.7734300	-1.6076710	0.0112020
H	-8.8556340	-1.5066650	0.0152280
H	-7.4629840	-2.1523550	-0.8855250
H	-7.4569180	-2.1585280	0.9020060
C	6.6085740	-0.4047450	0.0125910
F	7.2398240	0.5985280	-0.6145850
F	7.0167730	-1.5504430	-0.5518790
F	7.0629210	-0.4143420	1.2801920

$\mathbf{S}_1$, E= -1797.03070112

C	-3.6287540	-0.9334520	0.0002830
C	-3.1572820	0.4148810	0.0013460
C	-4.0776770	1.4903000	0.0057130
C	-5.4154520	1.2020430	0.0083510
C	-5.8773610	-0.1457600	0.0068420
C	-4.9754470	-1.2257110	0.0027900
C	-1.1600090	-0.7023070	-0.0053700
H	-3.7105750	2.5082850	0.0067670

H	-6.1628290	1.9851510	0.0116510
H	-5.3207480	-2.2500700	0.0016940
S	-2.2966090	-2.0499970	-0.0040330
N	0.1167350	-0.8697180	-0.0092740
C	0.9209640	0.2424670	-0.0106510
C	2.3318190	0.0750200	-0.0183550
C	2.9110430	-1.2222760	-0.0252270
C	3.1897790	1.2069760	-0.0228360
C	4.2824120	-1.3736200	-0.0341810
H	2.2606170	-2.0870030	-0.0249510
C	4.5594370	1.0428450	-0.0321170
H	2.7543040	2.1976230	-0.0209090
C	5.1123190	-0.2448910	-0.0415580
H	4.7203580	-2.3645840	-0.0431980
H	5.2110760	1.9085090	-0.0394280
O	0.4365190	1.4695940	-0.0051630
B	-0.9790140	1.8431560	0.0000940
F	-1.2941880	2.5760150	-1.1393850
F	-1.2879410	2.5680280	1.1463630
N	-1.8112070	0.5190010	-0.0021360
O	-7.1976210	-0.2905130	0.0096300
C	-7.7663870	-1.5976250	0.0089450
H	-8.8428700	-1.4570150	0.0115670
H	-7.4667540	-2.1438340	-0.8881600
H	-7.4627440	-2.1464390	0.9031020
C	6.5949920	-0.4138010	0.0101800
F	7.2458590	0.5950400	-0.5944120
F	7.0002800	-1.5552180	-0.5729340
F	7.0631810	-0.4543780	1.2787160

Dye 4

$\mathbf{S}_0$, E= -1460.16916447

C	-3.6867930	-1.1318510	0.0000050
C	-3.2432330	0.1957630	0.0000010
C	-4.1487330	1.2556000	0.0000010
C	-5.5023740	0.9512560	0.0000070
C	-5.9473840	-0.3781420	0.0000120
C	-5.0461580	-1.4349460	0.0000110
C	-1.2349380	-0.8820540	-0.0000020
H	-3.7912790	2.2777530	-0.0000020
H	-6.2276180	1.7557100	0.0000070

H	-7.0102780	-0.5863820	0.0000150
H	-5.3882900	-2.4622080	0.0000140
S	-2.3261350	-2.2219920	-0.0000020
N	0.0882260	-1.0782050	-0.0000060
C	0.8501370	-0.0001800	0.0000000
C	2.3038100	-0.1457160	0.0000000
C	2.8900980	-1.4229650	-0.0000090
C	3.1270630	0.9835010	0.0000090
C	4.2618680	-1.5592960	-0.0000090
H	2.2503300	-2.2965350	-0.0000140
C	4.5098710	0.8567700	0.0000080
H	2.6766340	1.9681450	0.0000140
C	5.0828600	-0.4202840	0.0000010
H	4.7336120	-2.5342480	-0.0000150
H	5.1265090	1.7454460	0.0000140
O	0.3928790	1.2194080	0.0000100
B	-1.0314300	1.6185310	-0.0000080
F	-1.3165030	2.3505930	-1.1428490
F	-1.3165230	2.3506140	1.1428140
N	-1.8532540	0.2954900	-0.0000030
O	6.4097740	-0.6487070	-0.0000090
C	7.2913380	0.4634160	0.0000190
H	7.1468180	1.0768310	0.8934760
H	8.2986290	0.0551620	0.0000290
H	7.1468470	1.0768530	-0.8934290

$\mathbf{S}_1$, E= -1460.16022245

C	3.6666180	-1.1584130	0.0000020
C	3.2100330	0.1935930	-0.0000000
C	4.1367210	1.2554740	0.0000020
C	5.4804000	0.9444860	0.0000040
C	5.9265440	-0.3954560	0.0000050
C	5.0252980	-1.4532050	0.0000040
C	1.2050290	-0.9074620	-0.0000020
H	3.7796270	2.2771610	0.0000010
H	6.2122730	1.7429920	0.0000060
H	6.9897810	-0.6016740	0.0000070
H	5.3696150	-2.4795280	0.0000050
S	2.3312620	-2.2597730	0.0000000
N	-0.0821260	-1.0646100	-0.0000020
C	-0.8732250	0.0461610	-0.0000040
C	-2.2850680	-0.1172230	-0.0000020
C	-2.8774140	-1.4164430	0.0000030

C	-3.1456410	1.0149960	-0.0000040
C	-4.2401040	-1.5615530	0.0000040
H	-2.2295320	-2.2833760	0.0000050
C	-4.5167320	0.8662940	-0.0000030
H	-2.7045990	2.0035160	-0.0000070
C	-5.0801680	-0.4259910	0.0000010
H	-4.7049600	-2.5401610	0.0000070
H	-5.1492980	1.7444140	-0.0000040
O	-0.3808830	1.2799570	-0.0000110
B	1.0344430	1.6356330	-0.0000010
F	1.3570740	2.3675230	1.1424930
F	1.3570850	2.3675340	-1.1424860
N	1.8605600	0.3056790	-0.0000030
O	-6.3978740	-0.6693040	0.0000020
C	-7.3051740	0.4241730	-0.0000030
H	-7.1710770	1.0388070	-0.8939250
H	-8.3022670	-0.0075520	-0.0000030
H	-7.1710800	1.0388140	0.8939140

Dye 5

$\mathbf{S}_0$, E= -1345.72917914

C	-2.9570550	-1.1440570	0.0000010
C	-2.5205560	0.1858960	-0.0000010
C	-3.4311540	1.2416130	-0.0000030
C	-4.7827820	0.9300670	-0.0000030
C	-5.2209540	-0.4019920	-0.0000020
C	-4.3150880	-1.4543530	0.0000000
C	-0.5090190	-0.8821970	0.0000020
H	-3.0789850	2.2655840	-0.0000040
H	-5.5123430	1.7305510	-0.0000050
H	-6.2828200	-0.6153010	-0.0000020
H	-4.6520740	-2.4832720	0.0000020
S	-1.5912210	-2.2267300	0.0000030
N	0.8183530	-1.0711190	0.0000010
C	1.5704430	0.0089960	0.0000010
C	3.0352090	-0.1290240	-0.0000000
C	3.6162550	-1.4025720	-0.0000040
C	3.8444980	1.0124140	0.0000030
C	4.9988420	-1.5304310	-0.0000050
H	2.9748350	-2.2748770	-0.0000060
C	5.2275880	0.8778000	0.0000030

H	3.3832490	1.9918130	0.0000060
C	5.8045660	-0.3914120	-0.0000020
H	5.4504050	-2.5151950	-0.0000080
H	5.8557500	1.7603880	0.0000050
H	6.8836500	-0.4936980	-0.0000020
O	1.1116600	1.2257690	-0.0000000
B	-0.3156850	1.6206200	0.0000010
F	-0.6029640	2.3490020	-1.1430090
F	-0.6029630	2.3490050	1.1430090
N	-1.1309820	0.2919590	0.0000020

$\mathbf{S}_1$, E= -1345.71647499

C	2.9377890	-1.1699980	0.0000000
C	2.4836090	0.1894610	-0.0000010
C	3.4138530	1.2519130	-0.0000020
C	4.7536100	0.9369460	-0.0000040
C	5.1962860	-0.4083520	-0.0000030
C	4.2954090	-1.4657970	-0.0000010
C	0.4764990	-0.9089370	0.0000020
H	3.0577240	2.2737870	-0.0000030
H	5.4894770	1.7314180	-0.0000050
H	6.2592530	-0.6153420	-0.0000040
H	4.6396080	-2.4918690	0.0000010
S	1.6041200	-2.2679330	0.0000030
N	-0.8029070	-1.0629240	0.0000020
C	-1.6012060	0.0575800	0.0000010
C	-3.0114970	-0.1066560	-0.0000000
C	-3.5939720	-1.4058700	0.0000010
C	-3.8684100	1.0275290	-0.0000030
C	-4.9677650	-1.5510790	-0.0000010
H	-2.9419290	-2.2698390	0.0000030
C	-5.2391150	0.8603990	-0.0000040
H	-3.4276620	2.0163190	-0.0000030
C	-5.8010260	-0.4251710	-0.0000040
H	-5.4027420	-2.5437990	-0.0000000
H	-5.8864340	1.7297430	-0.0000060
H	-6.8780360	-0.5460730	-0.0000050
O	-1.1018630	1.2811480	0.0000020
B	0.3162950	1.6393130	0.0000020
F	0.6362310	2.3646080	1.1432160
F	0.6362310	2.3646100	-1.1432120
N	1.1402960	0.3060750	0.0000000

Dye 6

$\mathbf{S}_0$, E= -1682.60932250

C	-4.2391190	-1.2159420	0.0037470
C	-3.8555360	0.1302680	0.0058310
C	-4.8066410	1.1499750	0.0147280
C	-6.1444830	0.7853940	0.0213850
C	-6.5298860	-0.5632750	0.0191360
C	-5.5842010	-1.5794370	0.0103360
C	-1.8060400	-0.8588940	-0.0081740
H	-4.4953410	2.1870900	0.0164530
H	-6.9050320	1.5563760	0.0284920
H	-7.5825760	-0.8177950	0.0244420
H	-5.8808880	-2.6205980	0.0087230
S	-2.8321990	-2.2437370	-0.0068120
N	-0.4686480	-0.9936710	-0.0155800
C	0.2339420	0.1146600	-0.0173080
C	1.7082370	0.0347060	-0.0241890
C	2.3374550	-1.2138760	-0.0242040
C	2.4670280	1.2083930	-0.0327010
C	3.7227730	-1.2892490	-0.0292260
H	1.7331380	-2.1116570	-0.0211520
C	3.8534320	1.1342680	-0.0385190
H	1.9674160	2.1681730	-0.0359400
C	4.4713810	-0.1137800	-0.0381290
H	4.2201270	-2.2510270	-0.0332290
H	4.4506040	2.0374590	-0.0499010
O	-0.2664330	1.3132810	-0.0128820
B	-1.7102960	1.6525440	-0.0014970
F	-2.0324580	2.3707370	-1.1397100
F	-2.0167470	2.3617900	1.1466750
N	-2.4711070	0.2901650	-0.0014370
C	5.9714250	-0.1920360	0.0135290
F	6.4387730	-1.3121610	-0.5562960
F	6.5519390	0.8457780	-0.6060450
F	6.4218690	-0.1861110	1.2824100

$\mathbf{S}_1$, E= -1682.59467280

C	-4.2114720	-1.2510410	0.0043800
C	-3.8188880	0.1290870	0.0056400
C	-4.7940200	1.1501480	0.0153900

C	-6.1174010	0.7759660	0.0233490
C	-6.4996890	-0.5896460	0.0216840
C	-5.5545040	-1.6069000	0.0122780
C	-1.7653600	-0.8778100	-0.0093340
H	-4.4833830	2.1867170	0.0164630
H	-6.8884660	1.5360350	0.0310370
H	-7.5525700	-0.8425210	0.0279600
H	-5.8539110	-2.6467960	0.0112840
S	-2.8308000	-2.2866060	-0.0068190
N	-0.4829590	-0.9790610	-0.0160150
C	0.2668950	0.1795230	-0.0190640
C	1.6804820	0.0763780	-0.0251500
C	2.3181180	-1.1955320	-0.0286220
C	2.4856780	1.2486090	-0.0307260
C	3.6950490	-1.2833860	-0.0339780
H	1.7075510	-2.0886570	-0.0279150
C	3.8597410	1.1461560	-0.0370360
H	2.0039240	2.2175690	-0.0314320
C	4.4700050	-0.1171920	-0.0416060
H	4.1786920	-2.2526070	-0.0395310
H	4.4727570	2.0395510	-0.0452450
O	-0.2835840	1.3745780	-0.0183600
B	-1.7195460	1.6751620	-0.0024660
F	-2.0760740	2.3884640	-1.1385780
F	-2.0544640	2.3753510	1.1484900
N	-2.4817740	0.3051160	-0.0027280
C	5.9610620	-0.2118800	0.0139420
F	6.4202680	-1.3573950	-0.5151860
F	6.5588040	0.7985350	-0.6400070
F	6.4263070	-0.1664310	1.2819470

Dye 7

$\mathbf{S}_0$, E= -1797.04956410

C	-2.4771650	-0.9344820	-0.0000030
C	-1.9842830	0.3755150	0.0000020
C	-2.8468170	1.4728820	0.0000090
C	-4.2076750	1.2260360	0.0000120
C	-4.6945300	-0.0895900	0.0000080
C	-3.8457530	-1.1859250	0.0000000
C	-0.0207120	-0.7784380	-0.0000070
H	-2.4501540	2.4799900	0.0000110

H	-4.9079880	2.0530980	0.0000180
H	-4.2360210	-2.1948840	-0.0000060
S	-1.1627330	-2.0771290	-0.0000070
N	1.2904420	-1.0223740	-0.0000090
C	2.0943080	0.0286410	-0.0000120
C	3.5391970	-0.1708990	-0.0000070
C	4.0790480	-1.4690120	-0.0000150
C	4.4026720	0.9287630	0.0000080
C	5.4447160	-1.6541070	-0.0000090
H	3.4086170	-2.3192180	-0.0000270
C	5.7792210	0.7526460	0.0000150
H	3.9879380	1.9289010	0.0000140
C	6.3059850	-0.5447710	0.0000060
H	5.8815370	-2.6451470	-0.0000160
H	6.4276130	1.6183460	0.0000270
O	1.6815750	1.2628220	-0.0000210
B	0.2747840	1.7176600	-0.0000140
F	0.0148040	2.4564760	-1.1427850
F	0.0148200	2.4564920	1.1427490
N	-0.5962920	0.4237150	-0.0000010
O	7.6227210	-0.8201170	0.0000140
C	8.5448720	0.2592330	0.0000260
H	8.4228430	0.8771770	0.8936050
H	9.5362480	-0.1861030	0.0000290
H	8.4228520	0.8771900	-0.8935450
C	-6.1824880	-0.2823740	0.0000100
F	-6.7555080	0.2863110	1.0761590
F	-6.7555060	0.2862680	-1.0761640
F	-6.5342400	-1.5743630	0.0000350

$\mathbf{S}_1$, E= -1797.04253289

C	2.4636380	-0.9551660	0.0000040
C	1.9560750	0.3744670	-0.0000020
C	2.8379750	1.4747560	-0.0000060
C	4.1907770	1.2222070	-0.0000020
C	4.6819180	-0.1007970	0.0000040
C	3.8332250	-1.1984300	0.0000080
C	-0.0011320	-0.8024410	-0.0000010
H	2.4411610	2.4813520	-0.0000120
H	4.8953850	2.0460110	-0.0000050
H	4.2250360	-2.2068190	0.0000130
S	1.1737430	-2.1084060	0.0000060
N	-1.2864220	-1.0085620	-0.0000020

C	-2.1130240	0.0677650	-0.0000050
C	-3.5221480	-0.1472950	-0.0000040
C	-4.0672960	-1.4676820	-0.0000050
C	-4.4199170	0.9572660	-0.0000020
C	-5.4228490	-1.6596380	-0.0000040
H	-3.3884890	-2.3104950	-0.0000070
C	-5.7834780	0.7627430	-0.0000000
H	-4.0106250	1.9591670	-0.0000010
C	-6.3019360	-0.5507520	-0.0000010
H	-5.8555710	-2.6526900	-0.0000040
H	-6.4472350	1.6172930	0.0000020
O	-1.6720100	1.3205420	-0.0000080
B	-0.2727150	1.7333740	-0.0000120
F	0.0243690	2.4739810	1.1421990
F	0.0243660	2.4739660	-1.1422350
N	0.6042410	0.4327830	-0.0000050
O	-7.6058510	-0.8381480	-0.0000000
C	-8.5572430	0.2196950	0.0000010
H	-8.4463320	0.8377730	-0.8943660
H	-9.5353090	-0.2529050	0.0000010
H	-8.4463310	0.8377720	0.8943680
C	6.1690450	-0.2883830	0.0000130
F	6.7400850	0.2837680	-1.0760900
F	6.7400680	0.2837280	1.0761460
F	6.5253760	-1.5784890	-0.0000080

Dye 8

$\mathbf{S}_0$, E= -1682.60925726

C	1.2762820	0.3713170	0.0000030
C	2.1300630	1.4756660	0.0000110
C	3.4924000	1.2388570	0.0000120
C	3.9891450	-0.0734690	0.0000050
C	3.1494820	-1.1762490	-0.0000040
C	-0.6761040	-0.7979720	-0.0000020
H	1.7259090	2.4797890	0.0000190
H	4.1866010	2.0710160	0.0000200
H	3.5473710	-2.1822020	-0.0000090
S	0.4733950	-2.0870060	-0.0000110
N	-1.9888870	-1.0518280	-0.0000030
C	-2.7960870	-0.0087630	-0.0000020
C	-4.2504620	-0.2196080	0.0000010

C	-4.7685490	-1.5203270	0.0000090
C	-5.1141150	0.8817630	-0.0000030
C	-6.1432400	-1.7147450	0.0000130
H	-4.0858670	-2.3606640	0.0000120
C	-6.4885900	0.6802880	0.0000000
H	-4.7011400	1.8824010	-0.0000090
C	-7.0028510	-0.6158330	0.0000080
H	-6.5468260	-2.7200430	0.0000190
H	-7.1590050	1.5311360	-0.0000030
O	-2.3978740	1.2277800	-0.0000040
B	-0.9937110	1.6967620	-0.0000060
F	-0.7410740	2.4351560	1.1429410
F	-0.7410690	2.4351370	-1.1429640
N	-0.1123840	0.4080720	0.0000050
C	5.4789180	-0.2546440	0.0000030
F	6.0466700	0.3185610	-1.0762240
F	6.0466730	0.3185930	1.0762120
F	5.8404360	-1.5437610	0.0000210
H	-8.0756580	-0.7704390	0.0000110

$\mathbf{S}_1$, E= -1682.59796635

C	-1.7641650	-0.9604150	-0.0000090
C	-1.2443100	0.3730270	-0.0000080
C	-2.1194610	1.4828960	-0.0000030
C	-3.4704810	1.2392110	0.0000020
C	-3.9724780	-0.0852100	0.0000030
C	-3.1372650	-1.1902800	-0.0000030
C	0.7019330	-0.8227300	-0.0000150
H	-1.7137190	2.4857850	-0.0000020
H	-4.1710560	2.0662280	0.0000080
H	-3.5387360	-2.1946970	-0.0000010
S	-0.4919470	-2.1232620	-0.0000170
N	1.9743270	-1.0400250	-0.0000140
C	2.8255930	0.0382900	-0.0000090
C	4.2267130	-0.1970460	-0.0000020
C	4.7431070	-1.5251370	-0.0000080
C	5.1375940	0.8944590	0.0000130
C	6.1074520	-1.7380320	0.0000020
H	4.0483430	-2.3550610	-0.0000180
C	6.4975240	0.6597740	0.0000210
H	4.7450970	1.9032570	0.0000170
C	6.9940920	-0.6534760	0.0000160
H	6.4938430	-2.7504380	-0.0000020

H	7.1881270	1.4949150	0.0000320
O	2.3882550	1.2847620	-0.0000100
B	0.9916050	1.7167240	-0.0000130
F	0.7047720	2.4526740	-1.1430950
F	0.7047680	2.4526780	1.1430660
N	0.1003250	0.4222990	-0.0000110
C	-5.4635580	-0.2573050	0.0000190
F	-6.0225440	0.3221400	1.0765770
F	-6.0225710	0.3221750	-1.0765050
F	-5.8316400	-1.5425810	0.0000030
H	8.0638350	-0.8273600	0.0000230

Dye 9

$\mathbf{S}_0$, E= -2019.48900783

C	3.0449630	-0.9407420	-0.0061900
C	2.6030320	0.3871730	-0.0021790
C	3.5063080	1.4516390	0.0058770
C	4.8558950	1.1519630	0.0098400
C	5.2919060	-0.1820820	0.0055850
C	4.4035250	-1.2453880	-0.0023340
C	0.6021500	-0.6927290	-0.0135850
H	3.1489930	2.4733480	0.0088990
H	5.5878610	1.9510280	0.0159160
H	4.7552200	-2.2685200	-0.0055830
S	1.6888590	-2.0315880	-0.0152390
N	-0.7243710	-0.8857000	-0.0188640
C	-1.4776320	0.1919460	-0.0180310
C	-2.9453550	0.0441250	-0.0225480
C	-3.5150090	-1.2327110	-0.0260510
C	-3.7578540	1.1818030	-0.0255680
C	-4.8954020	-1.3726600	-0.0298960
H	-2.8697110	-2.1014910	-0.0268840
C	-5.1388900	1.0427130	-0.0303130
H	-3.3037730	2.1639480	-0.0258010
C	-5.6974950	-0.2332880	-0.0339500
H	-5.3473910	-2.3564620	-0.0367910
H	-5.7778590	1.9169410	-0.0375460
O	-1.0306380	1.4099840	-0.0129490
B	0.3959750	1.8162470	-0.0044720
F	0.6750400	2.5342700	1.1434030
F	0.6854790	2.5450490	-1.1429090

N	1.2171050	0.4865130	-0.0069010
C	6.7723310	-0.4308510	0.0132120
F	7.3554830	0.0954330	1.1048190
F	7.0744050	-1.7347370	-0.0088420
F	7.3735350	0.1366650	-1.0472380
C	-7.1929050	-0.3786800	0.0170990
F	-7.8169890	0.6186130	-0.6261950
F	-7.6454480	-0.3642080	1.2848440
F	-7.6077700	-1.5303710	-0.5287590

$\mathbf{S}_1$, E= -2019.47533744

C	3.0241000	-0.9676050	-0.0061900
C	2.5708860	0.3921810	-0.0020730
C	3.4988900	1.4578110	0.0069360
C	4.8351210	1.1468390	0.0111360
C	5.2710700	-0.2027830	0.0062770
C	4.3848470	-1.2653080	-0.0023220
C	0.5685100	-0.7059780	-0.0140020
H	3.1436730	2.4796500	0.0103050
H	5.5766020	1.9371690	0.0179960
H	4.7369880	-2.2880730	-0.0058530
S	1.6987990	-2.0650530	-0.0150090
N	-0.7084430	-0.8653430	-0.0186980
C	-1.5108520	0.2576740	-0.0187360
C	-2.9180140	0.0861380	-0.0238980
C	-3.4919040	-1.2161780	-0.0286910
C	-3.7783090	1.2186570	-0.0267980
C	-4.8631240	-1.3712280	-0.0324180
H	-2.8382340	-2.0782090	-0.0297600
C	-5.1451210	1.0489460	-0.0320840
H	-3.3437020	2.2096150	-0.0264590
C	-5.6921270	-0.2437110	-0.0371530
H	-5.2993890	-2.3624590	-0.0384720
H	-5.8020140	1.9106280	-0.0386700
O	-1.0150770	1.4757490	-0.0149510
B	0.4044870	1.8448890	-0.0040850
F	0.7137420	2.5552830	1.1457680
F	0.7270030	2.5693720	-1.1413280
N	1.2303980	0.5074500	-0.0072820
C	6.7534760	-0.4467440	0.0132510
F	7.3284240	0.0898010	1.1024180
F	7.0579410	-1.7475790	-0.0036900
F	7.3440210	0.1219720	-1.0507570

C	-7.1789330	-0.4051400	0.0173930
F	-7.8160850	0.5472250	-0.6842500
F	-7.6490550	-0.3211550	1.2803900
F	-7.5816930	-1.5920370	-0.4608670

References

(S1) Le Bahers, T.; Adamo, C.; Ciofini, I. A Qualitative Index of Spatial Extent in Charge-Transfer Excitations. *J. Chem. Theory Comput.* **2011**, *7*, 2498–2506.

(S2) Humeniuk, A.; Bužančić, M.; Hoche, J.; Cerezo, J.; Mitrić, R.; Santoro, F.; Bonačić-Koutecký, V. Predicting fluorescence quantum yields for molecules in solution: A critical assessment of the harmonic approximation and the choice of the lineshape function. *J. Chem. Phys.* **2020**, *152*, 054107.

(S3) Ou, Q.; Peng, Q.; Shuai, Z. Toward Quantitative Prediction of Fluorescence Quantum Efficiency by Combining Direct Vibrational Conversion and Surface Crossing: BODIPYs as an Example. *J. Phys. Chem. Lett.* **2020**, *11*, 7790–7797.
